# Computational Methods Reveal a Series of Cyclic and Linear Lichenysins and Surfactins from the Vietnamese Marine Sediment-Derived *Streptomyces* Strain G222

**DOI:** 10.3390/molecules29071458

**Published:** 2024-03-24

**Authors:** Andrea Castaldi, Bich Ngan Truong, Quyen Thi Vu, Thi Hong Minh Le, Arul Marie, Gaël Le Pennec, Florent Rouvier, Jean-Michel Brunel, Arlette Longeon, Van Cuong Pham, Thi Mai Huong Doan, Marie-Lise Bourguet-Kondracki

**Affiliations:** 1 Molécules de Communication et Adaptation des Microorganismes, UMR 7245 CNRS, Muséum National d’Histoire Naturelle, 57 rue Cuvier (CP54), 75005 Paris, France; andrea.castaldi1@mnhn.fr (A.C.); arul.marie@mnhn.fr (A.M.); arlette.longeon@mnhn.fr (A.L.); 2 Institute of Marine Biochemistry (IMBC), Vietnam Academy of Science and Technology (VAST), 18 Hoang Quoc Viet, Caugiay, Hanoi 100000, Vietnam; ngantb77@gmail.com (B.N.T.); svuquyen@yahoo.com (Q.T.V.); lhminhbk@gmail.com (T.H.M.L.); phamvc@imbc.vast.vn (V.C.P.); 3 Laboratoire de Biotechnologie et Chimie Marines, Université Bretagne Sud, EMR CNRS 6076, IUEM, 56100 Lorient, France; gael.le-pennec@univ-ubs.fr; 4 UMR MD1 “Membranes et Cibles Thérapeutiques”, U1261 INSERM, Aix-Marseille Université, Faculté de Pharmacie, 27 Bd Jean Moulin, CEDEX 5, 13385 Marseille, France; florent.rouvier@univ-amu.fr (F.R.); jean-michel.brunel@inserm.fr (J.-M.B.)

**Keywords:** *Streptomyces* sp., linear and cyclic lipopeptide, lichenysin, surfactin, molecular networking, computational methods, NMR spectroscopy

## Abstract

The *Streptomyces* strain G222, isolated from a Vietnamese marine sediment, was confidently identified by 16*S* rRNA gene sequencing. Its AcOEt crude extract was successfully analyzed using non-targeted LC-MS/MS analysis, and molecular networking, leading to a putative annotation of its chemical diversity thanks to spectral libraries from GNPS and *in silico* metabolite structure prediction obtained from SIRIUS combined with the bioinformatics tool conCISE (Consensus Annotation Propagation of *in silico* Elucidations). This dereplication strategy allowed the identification of an interesting cluster of a series of putative cyclic and linear lipopeptides of the lichenysin and surfactin families. Lichenysins (**3**–**7**) were isolated from the sub-fraction, which showed significant anti-biofilm activity against *Pseudomonas aeruginosa* MUC-N1. Their structures were confirmed by detailed 1D and 2D NMR spectroscopy (COSY, HSQC, HMBC, TOCSY, ROESY) recorded in CD_3_OH, and their absolute configurations were determined using the modified Marfey’s method. The isolated lichenysins showed anti-biofilm activity at a minimum concentration of 100 µM. When evaluated for antibacterial activity against a panel of Gram-positive and Gram-negative strains, two isolated lichenysins exhibited selective activity against the MRSA strain without affecting its growth curve and without membranotropic activity. This study highlights the power of the MS/MS spectral similarity strategy using computational methods to obtain a cross-validation of the annotated molecules from the complex metabolic profile of a marine sediment-derived *Streptomyces* extract. This work provides the first report from a *Streptomyces* strain of combined cyclic and linear lichenysins and surfactins, known to be characteristic compounds of the genus *Bacillus*.

## 1. Introduction

The Gram-positive bacteria of the genus *Streptomyces* are the largest genus in the phylum Actinobacteria (class Actinomycetes, order Actinomycetales, family Actinomycetacae) [1]. *Streptomyces* strains have received particular attention as a prolific source of bioactive metabolites with a wide range of pharmacological activities, from anticancer and immunosuppressive to antibiotic activities. *Streptomyces* are responsible for the production of more than two-thirds of clinically used antibiotics of natural origin [2]. *Streptomyces* strains are ubiquitous in nature and have been found in soil, plants, insects, animals, and even marine organisms such as corals or marine sponges [3]. Recently, from the culture broth of *Streptomyces* sp. G248, obtained from the Vietnamese sponge *Halichondria panicea* collected from the coast of Da Nang, three new remarkable antimicrobial lavandulylated flavonoids were identified by our group [4]. The literature on *Streptomyces* from terrestrial sediment is also abundant, but in contrast, their occurrence from marine sediments has been poorly studied. However, one recent exciting study reported the isolation of 92 antibacterial *Streptomyces* strains from 2212 marine sediment-derived actinomycete strains from 11 sites in the Philippines archipelago. These strains were found to be effectively active against multidrug-resistant *Staphylococcus aureus*, *Pseudomonas aeruginosa*, and *Escherichia coli* [5,6]. These findings stimulated the interest of investigating marine sediments as a valuable niche for antibiotic drug discovery.

Recently, we have isolated several actinomycetes from sediments collected in the Quang Tri Sea of Vietnam. Quang Tri Province in the North Central Vietnam is a coastal province with 75 km of coastline. With potential in terms of geographical location and marine resources, the marine biodiversity within the Quang Tri Sea is considered to be extensive, but remains poorly understood and explored. The culture broths of each isolated actinomycetes were then extracted with AcOEt for antimicrobial biological screening. G222, identified using a 16*S* rRNA gene sequence analysis, showed antimicrobial activity. Therefore, strain G222 was selected for fermentation in order to analyze potentially bioactive secondary metabolites.

Investigations of the *Streptomyces* G222 AcOEt extract using LC-ESI-MS/MS analyses and molecular networking strategy [7,8], with spectral libraries of GNPS and *in silico* metabolite structure prediction combined with the bioinformatics tool conCISE (Consensus Annotation Propagation of *in silico* Elucidations) [9], led to the identification of a series of putative lipopetides belonging to the lichenysin and surfactin families, including cyclic and linear analogues (Figures S1–S16, Tables S1–S15). Isoforms of the cyclic lichenysin peptides (**3**–**7**) were also purified and their structures were confirmed using detailed examination of 1D and 2D NMR spectroscopic data and Marfey’s method for determining the absolute configuration of the amino acid residues. Lipopeptides are mainly known as excellent biosurfactants due to their amphiphilic nature. Previous studies have also reported their antimicrobial activity, although relatively low growth inhibitory effects were observed in the absence of synergistic metabolites [10]. Furthermore, since bacterial resistance to antibiotics is primarily due to the formation of bacterial biofilms during antibiotic therapy, antibiofilm compounds are of interest and are actively looked for. Biofilms are communities of bacteria that attach and anchor themselves to a surface matrix. They impede the penetration of antimicrobial agents, thereby interfering with their mechanisms of action and leading to persistent infections [11]. This study was therefore a good opportunity to investigate antibacterial potential and antibiofilm efficacy of the isolated lipopeptides. The cyclic lichenysin peptides (**3**–**4**) and (**6**–**7**) were therefore tested against Gram-positive and Gram-negative strains and for their ability to restore the activity of doxocycline and erythromycin. Surprisingly, the only antibacterial activity observed was against the methicillin-resistant *Staphylococcus aureus*, which was similar for both compounds. This result prompts us to perform a real-time growth inhibition curve and to investigate the effect on the ATP efflux pump. Furthermore, a weak anti-biofilm activity was observed in the crude extract and in the isolated compounds from 100 µM.

This paper presents the discovery of lipopeptides from a marine sediment-derived *Streptomyces* strain. Notably, this is the first report of lipopeptides belonging to both lichenysin and surfactin families from this strain, and also the first report of linear lichenysins.

## 2. Results

### 2.1. Phylogenetic Affiliation of the G222 Bacteria Based on 16S rRNA

**Figure 1:**
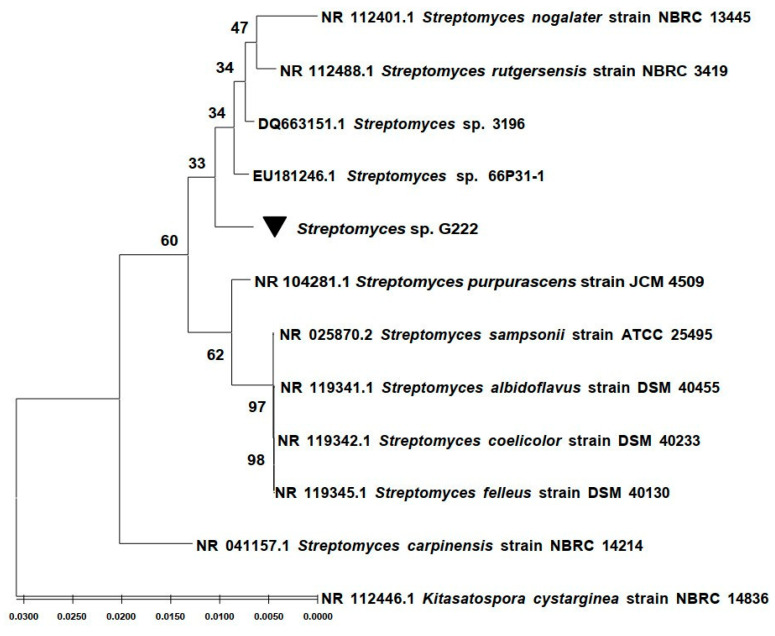
Phylogenetic tree based on the maximum-likelihood analysis of the bacterial 16S rRNA encoding gene.

The taxonomy of the strain G222 was identified using a 16*S* rRNA gene sequence. Gene sequences were handled with BioEdit v.2.7.5. and compared with 16*S* rRNA gene sequences in the GenBank database using the NBCI Blast program. The results showed that strain G222 belongs to the *Streptomyces* genus. It showed 99.58% identity with *Streptomyces* sp. 3196 (DQ663151.1) and *Streptomyces* sp. 66P31-1 (EU181246.1). Sequences were aligned using the ClustalW program, and statistical support was measured with Maximum Likelihood (ML) analysis using the MEGA software (version 11) (Figure 1). The 16*S* rRNA gene sequence of strain G222 was deposited on GenBank under accession number: OP763587.

### 2.2. LC-ESI-MS/MS Analysis of the Streptomyces G222 Derived Marine Sponge Crude Extract and Detection of Lichenysin and Surfactin Analogues

The dereplication approach was used by combining LC-MS/MS data with molecular networking obtained from the Global Natural Products Social Molecular Networking (GNPS) online platform [12]. Spectral library annotations from molecular networking were combined with *in silico* class predictions obtained from SIRIUS [13] and the bioinformatics tool ConCISE [9] (Consensus Classifications of *in silico* Elucidations) in order to propagate the putative classification of chemical families [14,15,16] for all subnetworks. The results were visualized using Cytoscape (ver. 3.9.1) [17] and revealed several clusters of unknown compounds (Figure S17).

**Figure 2:**
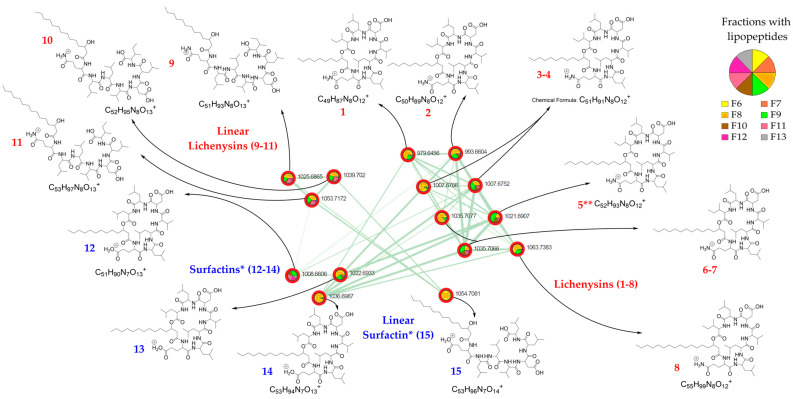
Molecular network of the lipopeptide cluster and annotation of the 15 nodes (**1**–**8**: cyclic lichenysins, **9**–**11** linear lichenysins, **12**–**14**: cyclic surfactins, **15**: linear surfactin) of the AcOEt extract of the marine sediment-derived *Streptomyces* G222 strain. The pie charts indicate the distribution of the lipopeptides in the different fractions of the fractionated crude extract. * In surfactins, Ile/Leu are possible in positions 2 and 7 of the lipopeptide, and in this figure, surfactins are depicted with Leu in position 2 and 7, according to the literature. ** *n-(iso)-C14* lichenysins **5a** and **5b**.

A cluster of 15 nodes (Figure 2) in the class of peptidomimetics, a subclass of lipopeptides, was particularly attractive.

Using GNPS (FBMN workflow version release_28.2), only seven of the fifteen nodes were annotated as lipopeptides, of which two as surfactins, three as lichenysins, and two of them as halobacillin, which is in fact a lichenysin [18]. These compounds are cyclic heptapeptides linked to a fatty acid (FA). Lichenysins are cyclic lipopeptides containing a glutamine (Gln) residue, and in surfactins Gln is replaced with a glutamic acid (Glu) residue. Considering the three possible isoforms in the acyl chain of the fatty acids (*n*, *ante-iso*, and *iso*), putative GNPS annotations were assigned twice to certain molecules. However, these putative annotations have accurately confirmed the correct distinction between lichenysins and surfactins. Using the SIRIUS algorithm, nine molecules in the cluster were putatively annotated as oligolipopeptide-like, of which six of them were proposed as lichenysins and three as surfactins, among which the molecular class prediction proposed some cyclic peptides and linear peptides. ConCISE allowed to propagate the putative classification of chemical families for the cluster of 15 nodes (Figure 2) as the natural product pathway of amino acids and peptides and/or polyketides, class of oligopeptides, and subclass of cyclic peptides and/or depsipeptides and/or lipopeptides. To confirm the putative annotations, all LC-MS-MS raw data were thoroughly analyzed (Figures S2–S16; Tables S1–S15).

Eight out of fifteen lipopeptides were found to be cyclic lipopeptides of the lichenysin family, and three of them differing from 1 Da were found to be cyclic lipopetides of the surfactin family (Table 1 and Table 2). Furthermore, linear lipopeptides were also suggested by the occurrence of peaks in the MS spectra with a difference of 18 Da from the pseudo-molecular mass peak and a shorter elution time than their cyclic counterpart, which was confirmed using mass fragmentation analysis [19]. Therefore, three linear lichenysins and one linear surfactin were also identified (Table 3). They all showed possession of the same amino acid residue composition (Leu/Ile-Leu/Ile-Val-Asp-Leu/Ile-Leu/Ile) linked to a Cx β-hydroxy fatty acid chain through a glutamine for lichenysin or a glutamic acid for surfactin variants (Figure 2).

In addition, a difference of 14 Da between lipopeptide compounds of the same series suggests a difference in the length of the alkyl chain. Therefore, on the basis of the mass fragmentation and in agreement with the literature, the eight cyclic lichenysins were hypothesized to be cyclo-Gln-Leu-Leu-Val-Asp-Leu-Ile-β-hydroxy fatty acids with a β-hydroxy fatty acid chain ranging in size from C11 to C15 and one C17 (Table 1), and the three linear lichenysins were hypothesized to have a C13 to C15 β-hydroxy fatty acid chain (Table 2). Similarly, the three cyclic surfactins were suggested to be cyclo-Glu-Leu-Leu-Val-Asp-Leu-Leu-β-hydroxy fatty acids with a chain size ranging from C13 to C15 β-hydroxy fatty acid chain (Table 3), and one surfactin was proposed to be a linear C15 β-hydroxy fatty acid chain (retention time at 19.3 min).

### 2.3. Characterization of Lichenysin Linear and Cyclic Lipopeptides: 1D and 2D NMR Spectroscopy

The crude extract of *Streptomyces* sp. G222 AcOEt was fractionated using silica gel flash chromatography, yielding 13 fractions (F1–F13). Fractions F8 and F9 containing lipopeptides were further selected for purification. F8 was fractionated on a Sephadex LH20 column, producing six sub-fractions; only F8-1 contained lipopeptides. F8-1 and F9 were subsequently purified using HPLC, as described in Section 4, affording a total of six lichenysin variants, namely *ante*-*iso*- and (*iso*)-C13 (**3** and **4**), *n*- and (*iso*)-C14 (**5a** and **5b**), and *ante*-*iso*- and (*iso*)-C15 lichenysins (**6** and **7**).

Lichenysin **3** showed a pseudo-molecular ion [M + H]^+^ at *m*/*z* 1007.6734, and lichenysin **4** showed a pseudo-molecular ion [M + H]^+^ at *m*/*z* 1007.6733. In the ^1^H NMR spectrum in CD_3_OH, seven NH doublet signals and eight distinct α-H signals were observed (Table S16). However, in the TOCSY spectrum, only seven α-protons correlated with the corresponding amide NH signals, indicating the presence of seven amino acid units (Table S16). The eighth α-H signal found to be involved in the fatty acid chain. Using the combined analysis of the two-dimensional NMR spectra namely, COSY, TOCSY, and HSQC, three leucines, one isoleucine, one valine, one aspartic acid, and one glutamine were confirmed, according to the mass spectra fragmentations. The HMBC data analysis led to the assignment of the CO signal of each amino acid as a cross peak with the corresponding proton at position α and/or β. The HMBC and ROESY spectra (Figures S25 and S26) led to the determination of the inter-residue linkages through the correlations between the seven amide protons with the carbonyl ^13^C signals of the following amino acids (Leu^2^-NH with Gln^1^-CO, Leu^3^-NH with Leu^2^-CO, Val^4^-NH with Leu^3^-CO, Asp^5^-NH with Val^4^-CO, Leu^6^-NH with Asp^5^-CO, Ile^7^-NH with Leu^6^-CO).

**Figure 3:**
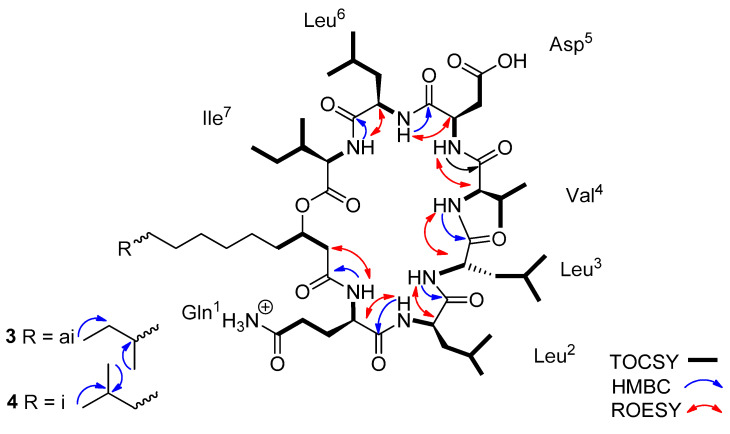
Key HMBC, TOCSY, and ROESY correlations of the lichenysins **3**–**4.** (*m*/*z* 1007.6734 and 1007.6733, respectively).

In the ^1^H NMR spectrum, two broad singlet signals in the aromatic region at δ 7.61 and 6.77 (Gln^1^-ε-NH_2_ a and b) and their HMBC correlations with Gln^1^-δ-C=O suggested the two terminal amide protons and confirmed the glutamine residue. The inter-residue linkage through the amide proton of glutamine with carbonyl ^13^C signal confirmed the presence of a fatty acid chain. In addition, the HMBC correlations between the protons Asp^5^-β and the Asp^5^-γ-C=O proved the aspartic acid residue. All this information led to define the planar structure of lichenysins **3** and **4** as being the cyclic lipo-heptapeptide cyclo-Gln^1^-Leu^2^-Leu^3^-Val^4^-Asp^5-^Leu^6^-Ile^7^-βOH-C13-FA (Figure 3).

Similarly, lichenysins (**5a**–**5b**) and (**6**–**7**) with the respective pseudo-molecular mass ion [M + H]^+^ at *m*/*z* 1021.6896 and 1035.7063, respectively, were found to be the cyclic lipo-heptapeptide cyclo-Gln^1^-Leu^2^-Leu^3^-Val^4^-Asp^5-^Leu^6^-Ile^7^-βOH-C14-FA and the cyclic lipo-heptapeptide cyclo-Gln^1^-Leu^2^-Leu^3^-Val^4^-Asp^5-^Leu^6^-Ile^7^-βOH-C15-FA, respectively (Figures S35 and S43).

Based on the presence of specific carbon signals in their ^13^C spectrum, key HMBC and ROESY correlations, and in agreement with the literature [20], the cyclic lichenysins at *m*/*z* 1007.6734 and *m*/*z* 1007.6733 ([M + H]^+^), and at *m*/*z* 1035.7063 were found to be the *ante-iso* and *iso* variants (0.8:0.2) and the *ante-iso* and *iso* (0.2:0.8) forms, respectively. The cyclic lichenysin at *m*/*z* 1021.6896 was revealed to be the *normal* and *iso*-forms (0.5:0.5).

Based on the given information, the three isolated lipopeptides were identified as being the C13, C14, and C15 variants of lichenysin G, respectively, as previously reported in the literature [21].

### 2.4. Absolute Configuration Using Modified Marfey’s Reagent of the Lichenysin Lipopeptides

Using the Marfey’s method, and according to the previously described biogenesis research [22]**,** the absolute configuration of both cyclic lichenysin amino acid residues was assigned as being L Gln^1^- L Leu^2^- D Leu^3^- L Val^4^- L Asp^5^- D Leu^6^- L Leu^7^ (Figure S44).

### 2.5. Antibiofilm Activities of the Lichenysin Lipopeptides

The clinical strain *Pseudomonas aeruginosa* MUC-N1 [23] was used to evaluate the biofilm formation activity of the crude extract sub-fractions and the isolated lipopeptides, as previously described [24].

While the AcOEt crude extract showed a very low inhibition of biofilm formation, (−12.17%), the subfraction F8 was demonstrated to significantly promote the biofilm formation (+157.0%). However, the sub-fraction F8-1, which contained a mixture of lipopeptides, mainly lichenysins (**3**–**4)** (*m*/*z* at 1007.6734 and 1007.6733) and (**6**–**7**) (*m*/*z* at 1035.7063), showed a significant antibiofilm activity (−71.47%). Nevertheless, when tested individually at 50 µM, they did not show any antibiofilm activity, even when they were combined [lichenysins (**3**–**4**) and (**6**–**7**) and (**5a**–**5b**) and (**6**–**7**)] and tested at the same concentration. However, antibiofilm activity was observed for the three compounds from 100 µM, in particular for lichenysins (**5a**–**5b**), which showed 41.27% of anti-biofilm formation (Figure S45).

### 2.6. Antimicrobial Activity

The antibacterial activity was evaluated for lichenysins (**3**–**4**) and (**6**–**7**) against a panel of Gram-positive and Gram-negative strains, including *S. aureus* ATCC 25923, *S. aureus* MRSA, *E. coli* ATCC25922, and *P. aeruginosa* ATCC 27853. Both lichenysins showed a selective activity against the MRSA strain with a CMI around 100 µM (Figure S46), without affecting its growth curve and without membranotropic activity 

## 3. Discussion

The aim of this study was first to profile the chemical diversity of the marine sediment-derived *Streptomyces* G222 strain. Its AcOEt crude extract was analyzed using a liquid chromatography-tandem mass spectrometry (LC-MS/MS) experiment and molecular networks. Of particular interest was the annotation of a cluster of lipopeptides thanks to the spectral libraries of GNPS and the *in silico* database of SIRIUS, which was validated using the ConCISE bioinformatics tool. This latter tool is an open-source graphic that can fuse molecular networks, spectral library matching, and *in silico* class predictions [9]. Therefore, ConCISE can be used to rapidly characterize complex molecular networks, and it is now developed in a wide range of untargeted metabolomics experiments.

In the present work, a specific cluster of fifteen nodes, annotated as lipopeptide, led to the identification of eight cyclic and three linear lichenysins, as well as three cyclic and one linear surfactins. Therefore, eight cyclic lichenysins having a C11 to C15 and C17 acyl chain length and three cyclic surfactins with a C13 to C15 carbonyl acyl chain length have been identified. It is noteworthy that cyclic lichenysins were observed to have a shorter elution time compared to their corresponding surfactin variants, as previously mentioned [21]. Several isoforms have been identified depending on the end of the acyl chain of the fatty acid, namely an *n*-, *iso*-, or *anteiso*- form [21].

Three cyclic lichenysins were isolated in sufficient quantity for further analysis in order to validate their structures and to evaluate their anti-biofilm and antibacterial activities. Therefore, the putative structures of lichenysins (**3**–**4**, **5a**–**5b** and **6**–**7**) [**3**–**4** (*m*/*z* at 1007.6734 and 1007.6733, **5a**–**5b** (*m*/*z* at 1021.6896) and **6**–**7** (*m*/*z* at 1035.7063)] were confirmed with detailed examination of their 1D and 2D NMR data. Key carbon signals in their ^13^C NMR spectrum and HMBC correlations allowed the identification of the *ante*-*iso* and *iso* (0.8:0.2), *iso* and *n* (0.5:0.5), and *ante*-*iso* and *iso* (0.2:0.8) forms, respectively. In a previous study, the three different forms (normal, *ante-iso*, and *iso*-branched) have already been reported for the JF2 surfactin biosurfactant obtained from the *Bacillus licheniformis* JF2 [20]. In addition, using the Marfey’s method and according to the biogenesis research, the absolute configuration of cyclic lichenysins 3–4 and 6–7 was assigned as being L Gln^1^- L Leu^2^- D Leu^3^- L Val^4^- L Asp^5^- D Leu^6^- L Leu^7^, as previously described [25].

In this study, from the same growth culture, three linear lichenysins and one linear surfactin were characterized, showing difference in the lengths of their acyl chain. So far, lichenysin variants have been essentially characterized in Gram-positive rod-shaped *Bacillus* strains, which are ubiquitous in the environment, mainly in *B. licheniformis*. Although surfactin derivatives were also known as characteristic metabolites of the genus *Bacillus*, they have already been isolated from the *Micromonospora* sp. CPCC 2,022,787 strain collected from a Chinese soil sample, in which the occurrence of two new linear surfactins and eight known cyclic surfactin derivatives were also identified [26]. Another study also reported the occurrence of cyclic and linear surfactin from *Bacillus megaterium* [19].

The production of these lipopeptides in growth culture was found to be dependent on the strain, culture medium, and incubation temperature, with optimal production between 35–45 °C [27]. Another paper on *Bacillus licheniformis* from food isolates highlighted the temperature-dependent factor in the production of lichenysins and surfactins. The authors speculated that the production of lichenysins would be lower at 55 °C, and would be optimal between 35–45 °C [28], as mentioned in the previous study [29]. Conversely, the production of surfactins would be lower at 45 °C than at 35 °C and higher at 27 °C than at 25 °C [30]. In our study, the *Streptomyces* strain, cultured at 28 °C, yielded more lichenysins than surfactins, indicating the interest of developing further studies with different temperature culture of this strain. In a recent investigation, optimizing the production of lichenysin-like polypeptides has been achieved through phosphate deprivation and the addition of 5% saccharose. The authors explained this effect by the earlier increase of lichenysin synthetase A gene expression (*Ich*AA) [31].

Lichenysins and surfactins are amphiphilic lipopeptides, best known for their strong surfactant activity. Lichenysins have been proven to be at least 10 times more powerful biosurfactants than surfactins [21]. Lipopeptides are the most potent biosurfactants used in a wide range of industries for medical and food safety applications. Their biosynthesis, which has been extensively studied, is a real challenge due to the high cost and low yield of their production. Therefore, numerous strategies are actively sought to improve their production, including fermentation, genetic, and metabolic engineering approaches [32]. Cyclic surfactins were also been reported to be more effective against HIV-1, with a stronger inhibitory activity than the linear surfactin derivatives from *Micromonospora* sp. CPCC 202,787 [26].

In our study, the isolated lichenysins **3**–**4** and **6**–**7** did not exhibit any antibacterial activit**ies** against a panel of Gram-positive and Gram-negative bacterial strains, but they showed a specific activity against the methicillin-resistant *Staphylococcus aureus*, with a similar MIC value of 100 µM. However, they did not enhance the activity of either doxycycline or erythromycin against resistant bacteria. In addition, they did not alter the kinetic antibacterial activity, as observed in the growth inhibition curve of the methicillin-resistant *S. aureus*. Furthermore, they did not affect the efflux pump, eliminating any membranotropic activity. From food isolates, it was demonstrated that lichenysin variants were slightly more toxic for Caco-2 human intestinal epithelial cells than surfactin variants [28]. Interestingly, a recent study on the marine thermotolerant *Bacillus licheniformis* B3–15 highlighted the anti-adhesive and antibiofilm properties of the crude biosurfactant (BS B3–15). The authors demonstrated that although the extract of the *B. licheniformis* BS B3–15 did not exhibit antibacterial activity, it influenced the initial adhesion of *Pseudomonas aeruginosa* ATCC 27853 and *S. aureus* ATCC 29213 on polystyrene surfaces (47% and 36%, respectively, at 300 µg. mL^−1^), and was also able to disrupt their preformed biofilms [31]. In the present work, a weak anti-biofilm formation against *P. aeruginosa* MUC-N1 was observed in the crude extract, and sub-fractions revealed to exert pro-biofilm activity. However, subsequent purifications revealed anti-biofilm activity and the isolated lichenysins tested individually only showed anti-biofilm formation activity from 100 µM.

Although there is a wealth of literature on these lipopeptides, they have rarely been isolated, which explains the different biological activities reported from their crude extracts. Given the complexity of marine crude extracts, computational methods could provide a rapid approach to their identifications. This study on a marine sediment-derived *Streptomyces* strain allowed the first report on cyclic and linear lichenysins and surfactins, suggesting the development of similar further studies for rapid identification of biosurfactants in diverse samples.

## 4. Materials and Methods

### 4.1. General Experimental Procedures

The mass spectra were recorded using an ultra high-resolution ESI-QTOF mass spectrometer (A MAXIS II). NMR spectra were obtained using a Bruker Avance 400 or 600 spectrometer using a typical pulse sequence. Flash chromatography was carried out using the Buchi C-615, C-601, and C-605 pump system (Rungis, France). Analytical reversed-phase (Luna C18, 250 × 4.6 mm, 5 μm, Phenomenex^®^, Torrance, CA, USA) column was performed with an Agilent Infinity (model 1220 LC) in conjunction with a photodiode array detector (model 1220 DAD Infinity LC) and the software OpenLab CDS (ver. 2.13). The data station captured the wavelength ranging from 200 to 600 nm. Sephadex^®^ LH-20 (Amersham Pharmacia, Uppsala, Sweden) and silica gel (200–400 mesh; Merck, Darmstadt, Germany) were used for chromatography columns (CC).

### 4.2. Sampling and Strain Isolation

Strain G222 was isolated from the sediment sample that was collected in the sea of Quang Tri, Vietnam, in May 2021, geographic coordinate 17°06′18″–107°05′38″ at a depth of 8 m; seawater temperature at the sampling site was 28 °C. The sediment sample was put into 15 mL or 50 mL sterile Falcon tube, preserved in an ice-box, and processed within 24 h.

### 4.3. Isolation of Strain G222

First, an aliquot of the sediment sample (0.5 g) was suspended in 4.5 mL of sterile distilled water, homogenized with vortexing for 1 min, and the suspension was treated using a wet-heat technique (60 °C for 6 min). Next, 0.5 mL of this suspension was transferred to another 4.5 mL sterile distilled water, and this step was repeated to set up a ten-fold dilution series to 10^−3^. At the final dilution step, aliquots of 50 µL were spread on ISP2 medium Petri dishes (soluble starch 5 g∙L^−1^; yeast extract 2 g∙L^−1^; malt extract 10 g∙L^−1^; glucose 10 g∙L^−1^; instant ocean 30 g∙L^−1^; agar 15 g∙L^−1^). These media were supplemented with 50 µg∙mL^−1^ of polymycin B and cycloheximide to inhibit Gram-negative bacterial and fungal contamination. After 7 days of aerobic incubation at 28 °C, the colony of actinomycete strain was transferred onto Petri new dish of medium ISP2 for purification. The strain G222 was identified belonging to genus *Streptomyces* using 16S rRNA gene sequence analysis.

### 4.4. Strain Identification Using 16S rRNA Gene Sequencing

Genomic DNA of strain G222 was extracted using Gen Elute Bacterial Genomic DNA kit (Sigma, Saint Louis, MO, USA). The 16S rRNA gene sequence was amplified with primers 9 F (5′-GAGTTTGATCCTGGCTCAG3′) and 1541R (5′-AAGGAGGTGATCCAACC3′). The reaction was performed in a 25.0 μL mixture containing: 10 μL of dH_2_O, 12.5 μL Platinum™ Hot Start PCR Master Mix (Invitrogen, Waltham, MA, USA), 1.0 μL for both 0.05 mM of primers, and 0.5 μL of genomic DNA. PCR program included a preheat at 94 °C for 3 min, followed by 30 cycles of denaturation at 94 °C for 1 min, annealing at 60 °C for 30 s, and elongation at 72 °C for 45 s before a final extension of 72 °C for 10 min. The PCR product was purified using the DNA purification kit (Invitrogen, Waltham, MA, USA) and then sequenced using a DNA Analyzer (ABI PRISM 3100, Applied Bioscience, Foster City, CA, USA).

### 4.5. Fermentation of the Streptomyces sp G222

The selected actinomycetes (G222) were inoculated in 20 flasks (each 5L flask containing 2.5 L of A1-broth medium: soluble starch 10 g∙L^−1^; yeast extract 4 g∙L^−1^; peptone 2 g∙L^−1^; CaCO_3_ 1 g∙L^−1^; FeSO_4_ 8mg∙mL^−1^; KBr 20 mg∙mL^−1^; instant ocean 30 g∙L^−1^; pH 7.0). The flasks were shaken at 150 rpm at 28 °C and harvested on the 10th day.

### 4.6. Preparation of the Crude Extract

The fermentation broth (50 L) was extracted with ethyl acetate (5 × 30 L). The combined ethyl acetate extracts were then concentrated under reduced pressure to give 15.5 g of crude extract.

### 4.7. LC-MS^2^ Analyzes of the AcOEt Extract

A high-resolution electrospray ionization-quadrupole time-of-flight (ESI-Q-TOF) mass spectrometer (MaXis II ETD, Bruker Daltonics, Billerica, MA, USA) connected to an HPLC system (Ultimate 3000 RSLC, Thermo Scientific, Waltham, MA, USA) enabled LC-ESI-HRMS2 analyses to be performed.

The LC separation was performed on an Acclaim RSLC Polar Advantage II column (2.2 µm, 2.1 × 100 mm, Thermo Scientific) with a flow rate of 0.3 mL.min^−1^. An isocratic flow of 1% B was maintained from 0 to 1 min (A: H_2_O + 0.1% formic acid, B: ACN + 0.08% formic acid). A linear gradient from 1% to 70% B in 4 min was initially applied, then an isocratic flow of 70% B over 10 min, followed by a linear gradient from 70% to 100% of B in 1 min, and lastly 100% of B in 3 min, and a decrease gradient to 5% in 1 min, for a total run duration of 29 min. The positive ion mass range (*m*/*z*) was recorded from 50 to 1300.

The source settings were as follows: 2.4 bar for the nebulizer gas, 200 °C for the dry heater, 8.0 L.min^−1^ for the dry gas, 3500 V for the capillary voltage, 500 V for the end plate offset, and 2000 V for the charging voltage. The collision energy of 40.0 eV was chosen for the auto MS/MS mode in LC-MS/MS using the same settings as the MS method. A sodium formate solution was immediately added as an internal reference for calibration during the first thirty seconds of the experiment. A measure of the internal calibrator was implemented by establishing a permanent MS/MS exclusion list criterion to prevent oversampling. The data were analyzed using Bruker Compass Data Analysis (ver. 4.4).

### 4.8. Mass Spectrometry: LC-MS/MS Data Processing

Data Analysis 4.4 (Bruker Daltonics) was used to convert the MS2 data files from the standard .d data-format to the .mzXML format. All .mzXML files were then imported into MZmine 3.4.27 [33]. Mass detection was carried out on centroid masses with the noise level set to 2.0E1 for MS2 and 1.0E3 for MS1, respectively. The ADAP chromatogram builder was used to build a chromatogram with a minimum group size of five scans, a group intensity threshold of 3E3, a minimum peak intensity of 3E3, and a *m*/*z* tolerance of 10 ppm was constructed [34]. Chromatogram resolution was achieved using the ADAP resolver method with the following settings: minimum feature height = 1E3, RT wavelet range = 0.1–0.5, peak duration range = 0.02–1.00, S/N threshold 70, chromatographic threshold = 10%. An *m*/*z* tolerance range of 0.03 Da and an RT feature edge filter were used for MS/MS pairing. The isotopic peak grouper method was used to categorize isotopes, with the lowest peak having an RT tolerance of 0.15 min and an *m*/*z* tolerance of 10 ppm. The adducts [M + Na–H], [M + K–H], [M + Mg–2H], [M + NH_3_], [M–Na + NH_4_], [M + 1, ^13^C] were eliminated by setting the maximum relative height to 100%. Only rows containing MS2 were retained in the resulting peak list after filtering.

Clustered data were then generated using the dedicated “Molecular networking files (e.g., GNPS, FBMN, IIMN, MetGem)” option and “Sirius/CSI-FingerID” with an .mgf file for GNPS and SIRIUS, while chromatographic data including retention times, peak areas, and peak heights were exported to a .csv file.

### 4.9. Mass Spectrometry: Molecular Networking

A molecular network was generated using the online FBMN workflow (version release_28.2) on GNPS (https://gnps.ucsd.edu/ProteoSAFe/status.jsp?task=de168cddecfe40248fa3ba5077705e55), accessed on 23 May 2023 (Figure S17). Both the MS/MS fragment ion tolerance and the parent mass tolerance were set at 0.02 Da. A network was then built by filtering edges with more than six matching peaks and a cosine score greater than 0.65. Furthermore, edges between two nodes were retained in the network if, and only if, they were in each other’s top ten most similar nodes. The network’s spectra were then checked against the GNPS spectral libraries. A minimum of five matched peaks and a score greater than 0.65 were required for all matches between network spectra and library spectra to be retained.

### 4.10. ConCISE (Consensus Classifications of In Silico Elucidations)

After adding the GNPS Task ID, the CANOPUS summary table (.txt), and the Networking information table (.tsv), the default thresholds were set at 50% for superclass, 70% for class, and 80% for subclass based on the ClassyFire ontologies. For the molecular networking job, ConCISE produced a single.csv file containing the full chemical hierarchy of the consensus annotation, a score, the ontology level at which ConCISE discovered a consensus annotation, the number of nodes that were used to construct the consensus, and the source (library or *in silico*). This .csv file was then imported into Cytoscape (ver. 3.9.1) to visualize the potential chemical families within each cluster of the molecular networking.

### 4.11. Isolation and Purification of the Lichenysin Lipopetides

The *Streptomyces* sp. G222 AcOEt crude extract (13 g) was subjected to silica gel flash chromatography, with the eluting solvent being a gradient mixture of CH_2_Cl_2_/MeOH from 0% to 100% of MeOH. A total of 13 fractions (F1 to F13) were obtained (Figure S18) which were analyzed using ultra-high-performance liquid chromatography-tandem mass spectrometry (UPLC-ESI-QToF-MS). Fractions F8 (0.367 mg) and F9 (1.6 g) were selected for isolation on the basis of their intensity, as they mainly showed the presence of lipopeptides. Fraction F8 was chromatographed on a Sephadex^®^ LH20 column using a CH_2_Cl_2_/MeOH elution gradient system from 0% to 100% of MeOH. This gave a total of 6 sub-fractions (F8-1 to F8-6) which were analyzed using ultra-high-performance liquid chromatography-tandem mass spectrometry (UPLC-ESI-QToF-MS). Only the subfraction F8-1 showed the presence of lipopeptides. Subfraction F8-1 and fraction F9 were therefore further purified by HPLC on an analytical reverse-phase column (Luna C18, 250 × 4.6 mm, 5 μm, Phenomenex^®^) using a modified protocol of lipopeptide isolation [35]. Briefly, mobile phases of water (Phase A) and acetonitrile (phase B), with 0.1% formic acid in both phase, were used as follows: injection start (30:70), 2 min (20:80), 12 min (10:90), 14 min (0:100), 16 min (0:100), 18 min (30:70), and 20 min (30:70); a flow rate of 1.5 mL.min^−1^ was applied during 20 min, recorded at 210 nm (Figure S19). The separation yielded (ante-*iso*)-and (*iso*)-C13 lichenysins (**3**–**4**) (1.01 mg, Rt = 11.2 min) (*m*/*z* at 1007.6734 and 1007.6733, respectively), *(n*)- and (*iso*)-C14 lichenysins (**5a**–**5b**), (0.66 mg, Rt = 14.1 min) (*m*/*z* at 1021.6896), and (*ante*-*iso*)- and (*iso*)-C15 lichenysins (**6**–**7**) (1.89 mg, Rt = 16.1 min) (*m*/*z* at 1035.7063).

Their MS/MS spectrum acquired on Bruker Maxis was deposited in the GNPS spectral library under the identifier CCMSLIB00012183962, and CCMSLIB00012183961 for lichenysins (**3**–**4**); CCMSLIB00012183963 for lichenysins (**5a**–**5b**) and CCMSLIB00012183959, and CCMSLIB00012183958 for lichenysins (**6**–**7**).

### 4.12. Advanced Marfey’s Analysis

The absolute configuration of the amino acid residues was determined using the advanced Marfey’s method, as previously described [36]. Briefly, all compounds (100 µg) were hydrolyzed with 6 M HCl at 100 °C for 8 h. Residual HCl vapors were removed under air filtered through 0.2-μm membrane filters at room temperature. The hydrolysates were then dissolved in 1 M NaHCO_3_ (20 µL) and the solutions were treated with 50 μL of 1% 1-fluoro-2,4-dinitrophenyl-5-L-alaninamide (L-FDAA) in acetone. The vials were heated at 40 °C for 1 hr. The mixtures were then neutralized with 1 M HCl (20 µL), dried, and the resulting L-FDAA derivatives were redissolved in MeOH (100 μL) for subsequent analysis. Authentic solutions at 50 mM of the standards L-Leu, L-Ile, L-Val, L-Gln, L-Asp were treated with L-FDAA and D-FDAA, as described above, to give the L-FDAA and D-FDAA standards. Marfey’s derivatives were analyzed using HPLC-ESI-HRMS, and their retention times were compared with those of the authentic standard derivatives. Mass spectra were recorded, as already described above. A 0.3 mL.min^−1^ flow rate was employed with an Acclaim RSLC Polar Advantage II column (2.2 µm, 2.1 × 100 mm, Thermo Scientific). The mobile phase was a mixture of 100% MilliQ water with the addition of 0.1% formic acid (Phase A) and 100% acetonitrile (Phase B) with the addition of 0.08% formic acid. A linear gradient was applied over 16 min, starting from 5% to 50% B in 2 min, then to 90% B in 2 min, and held for 2 min, and finally a decrease was to 5% in 5 min, for a total duration of 25 min. The positive ion mass range (*m*/*z*) is acquired from 50 to 1300. The data were treated with Data Analysis 4.4 (Bruker Daltonics).

### 4.13. Biofilm Formation Activity

Biofilm formation activity was assessed against the clinical strain *Pseudomonas aeruginosa* MUC-N1 according to the previously reported protocol [24]. Briefly, the bacterial strains were cultured in triplicate overnight at 37 °C with orbital shaking at 125 rpm in 5 mL of LB medium (10 g.L^−1^ NaCl, 10 g.L^−1^ tryptone, and 5 g.L^−1^ yeast extract). Except for the negative control, 200 mL of diluted culture adjusted to an optical density at 600 nm (OD_600_) of 0.05 in fresh test media was added to each well. Aliquots of crude extract or fractions were added to the 200 mL *P. aeruginosa* solutions at a concentration of 50 μg. mL^−1^ (1% DMSO). The 96-well microtitre plate (Thermo Scientific^TM^ Nunc^TM^ MicroWell^TM^, Waltham, MA, USA) was incubated in a stationary phase for 24 h. Using a TECAN Infinite M1000 (Männedorf, Switzerland) to measure OD_600_, the biofilm was stained and quantified according to the Coffey and Anderson protocol by measuring the OD_550_ after the addition of acetic acid [37]. The evaluation of the biofilm formation was adjusted according to the growth of the bacterial strains. Values are expressed as mean ± SD. A version of GraphPad Prism 8.01 was used to analyze the data.

### 4.14. Antimicrobial Assays

The antimicrobial efficacy of lichenysins (**3**–**4**) and (**6**–**7**) was systematically assessed against the Gram-negative bacterial strains, including *Escherichia coli* (ATCC 25922) and *Pseudomonas aeruginosa* (ATCC 27853), as well as the Gram-positive *Staphylococcus aureus* (ATCC 25923) and its methicillin-resistant strain *S. aureus* (MRSA). The evaluation followed a well-established protocol [38], using the standard broth dilution in accordance with the recommendations of the Comité de l’Antibiogramme de la Société Française de Microbiologie (CA-SFM). Minimum inhibitory concentrations (MICs) were determined using an inoculum of 10^5^ CFU in 200 µL of Mueller–Hinton broth (MHB) containing two-fold serial dilutions of each lichenysin. The MIC was defined as the lowest drug concentration that completely inhibited visible growth after incubation for 18 h at 37 °C. These measurements were performed independently in triplicate to ensure robustness and reliability.

In addition, to assess the potential synergistic effects, the recovery of activity of doxycycline and erythromycin against *P. aeruginosa* (ATCC 27853) and *E. coli* (ATCC 25922) was evaluated to assess potential synergistic effects. Lichenysins (**3**–**4**) and (**6**–**7**) were tested separately in combination with the respective antibiotic at a dose at which they are individually ineffective, i.e., 2 µg.mL^−1^ [38]. This aim of this approach was to elucidate any enhanced antimicrobial activity resulting from the combination of lichenysins and antibiotics.

### 4.15. Real-Time Growth Curves

To assess the kinetic antibacterial activity against *S. aureus* ATCC 25923, solutions of lichenysins (**3**–**4**) and (**6**–**7**) were subjected to rigorous testing in triplicate, following a standardized protocol [38]. In a 96-well plate, 10 µL of different stock solutions for both compounds were dispensed, along with 190 µL of a bacterial suspension at 5·10^5^ CFU.mL^−1^ in brain heart infusion (BHI) broth. Positive controls, consisting of 200 µL of a 5.10^5^ CFU.mL^−1^ bacterial suspension in BHI, and negative controls, consisting of only 200 µL of BHI broth, were also included. The plate was incubated at 37 °C in a TECAN Spark Reader (Roche Diagnostic, Rotkreuz, Switzerland), and real-time bacterial growth was monitored at every 20 min interval over a period of 18 h by measuring the optical density at 590 nm (OD_590_). This approach allowed a comprehensive evaluation of the dynamic antibacterial effects of lichenysins (**3**–**4**) and (**6**–**7**) against *S. aureus* ATCC 25923.

### 4.16. ATP Efflux Measurement

The compounds were meticulously dissolved in double-distilled water, achieving a concentration of 100 µg.mL^−1^. Meanwhile, the bacterial suspension was prepared in BHI broth. Subsequently, 90 µL of each bacterial suspension was combined with 10 µL of the respective tested compound solution, and the mixture was vigorously shaken for 3 min at 37 °C. This blending process occurred within a 96F untreated white microwell plate (Thermo Scientific 236015). Following the incubation period, 50 µL of a luciferin–luceferase reagent (Yelen, Brest, France) was introduced to the mixture, and the luminescence signal was quantified using a TECAN Spark microplate reader (Tecan). The quantification involved six readings performed over 30 sec each. Notably, ATP concentration was quantified through internal sample addition. For benchmarking, squalamine (100 µg. mL^−1^) was employed as a positive control to establish the maximal level of ATP efflux. This meticulous assay was conducted in three independent experiments, ensuring robustness and reliability in the obtained results [38].

## 5. Conclusions

The marine sediment isolate G222, collected from Quang Tri Province, North Central Vietnam, was identified as belonging to the genus *Streptomyces* using 16*S* rRNA gene sequence analysis (GenBank accession number: OP763587), and shared 99.58% identity with *Streptomyces* sp. 3196 (DQ663151.1) and *Streptomyces* sp. 66P31-1 (EU181246.1).

Chemical investigation of its AcOEt extract led to the identification of a series of cyclic and linear lichenysins and surfactins using a non-targeted LC-MS/MS experiment combined with molecular networking, spectral library matching, and *in silico* prediction, as well as cross-validation using the new bioinformatics tool ConCISE. These compounds are known as power biosurfactants produced by *Bacillus* strains. However, this study represents their first identification from a marine-derived *Streptomyces* strain G222. The compounds were found for the first time as a mixture of cyclic and linear variants of both lichenysins and surfactins.

Lichenysins (**3**–**7**) have been isolated, the predicted planar structures have been confirmed, and detailed NMR data, including COSY, HMBC, TOCSY and ROESY data of these known lichenysins, have been obtained. Their absolute configurations were determined using the modified Marfey’s method. Furthermore, antibacterial activities and biofilm formation activity of these lichenysins were investigated, and it was found that they exhibited anti-biofilm activity from 100 µM and antibacterial activity against the MRSA strain. However, it should be noted that no effect on their growth curve was observed, and no membranotropic activity was detected.

The present study introduces new avenues for additional sources of lipopeptides and highlights computational methods as solid ways to rapidly and accurately identify them. This should stimulate further investigations to enhance their production.

## Figures and Tables

**Table 1:** Cyclic lichenysins (**1**–**8**) containing a Cx β-hydroxy fatty acid chain and their retention time.

Cpd	Exact Mass	Chemical Formula	Lichenysin Precursor Ion [M + H]^+^ at *m*/*z*	Retention Time in Min	Cyclic Lichenysin Containing a Cx β-Hydroxy Fatty Acid Chain
**1**	979.64380	C_49_H_87_N_8_O_12_^+^	979.6434	17.6	C11
**2**	993.65945	C_50_H_89_N_8_O_12_^+^	993.6581	18.4	C12
**3**	1007.67510	C_51_H_91_N_8_O_12_^+^	1007.6734	17.9	C13
**4**	1007.67510	C_51_H_91_N_8_O_12_^+^	1007.6733	18.5	C13
**5**	1021.69075	C_52_H_93_N_8_O_12_^+^	1021.6896	19.4	C14
**6**	1035.70640	C_53_H_95_N_8_O_12_^+^	1035.7063	20.0	C15
**7**	1035.70640	C_53_H_95_N_8_O_12_^+^	1035.7063	20.2	C15
**8**	1063.73770	C_55_H_99_N_8_O_12_^+^	1063.7369	21.2	C17

**Table 2:** Cyclic surfactins (**12**–**14**) containing a Cx β-hydroxy fatty acid chain and their retention time.

Cpd	Exact Mass	Chemical Formula	Lichenysin Precursor Ion [M + H]^+^ at *m*/*z*	Retention Time in Min	Cyclic Surfactin Containing a Cx β-Hydroxy Fatty Acid Chain
**12**	1008.65911	C_51_H_90_N_7_O_13_^+^	1008.6618	19.3	C13
**13**	1022.67476	C_52_H_92_N_7_O_13_^+^	1022.6767	19.9	C14
**14**	1036.69041	C_53_H_94_N_7_O_13_^+^	1036.6906	20.5	C15

**Table 3:** Linear lichenysins (**9**–**11**) containing a Cx β-hydroxy fatty acid chain and their retention time.

Cpd	Exact Mass	Chemical Formula	Lichenysin Precursor Ion [M + H]^+^ at *m*/*z*	Retention Time in Min	Linear Lichenysin Containing a Cx β-Hydroxy Fatty Acid Chain
**9**	1025.68566	C_51_H_93_N_8_O_13_^+^	1025.6832	17.4	C13
**10**	1039.70131	C_52_H_95_N_8_O_13_^+^	1039.6990	18.1	C14
**11**	1053.71696	C_53_H_97_N_8_O_13_^+^	1053.7147	18.8	C15

## Data Availability

Data are contained within the article and Supplementary Materials.

## Supplementary Materials

The following supporting information can be downloaded at: https://www.mdpi.com/article/10.3390/molecules29071458/s1, Figure S1: (A) The molecular networking obtained through the LC-MS/MS analysis of the extract of G222. (B) Zoom view of the discriminant cluster of the lipopeptides; Figure S2: Fragmentations pattern and positive ion mode high-resolution ESI MS/MS spectrum for Lichenysin (**1**) (*m*/*z* 979.6434 [M + H]^+^); Figure S3: Fragmentations pattern and positive ion mode high-resolution ESI MS/MS spectrum for Lichenysin (**2**) (*m*/*z* 993.6581 [M + H]^+^); Figure S4: Fragmentations pattern and positive ion mode high-resolution ESI MS/MS spectrum for Lichenysin (**3**) (*m*/*z* 1007.6734 [M + H]^+^); Figure S5: Fragmentations pattern and positive ion mode high-resolution ESI MS/MS spectrum for Lichenysin (**4**) (isoform of **3**) (*m*/*z* 1007.6733 [M + H]^+^), Figure S6: Fragmentations pattern and positive ion mode high-resolution ESI MS/MS spectrum for Lichenysin (**5**) (*m*/*z* 1021.6896 [M + H]^+^); Figure S7: Fragmentations pattern and positive ion mode high-resolution ESI MS/MS spectrum for Lichenysin (**6**) (*m*/*z* 1035.7063 [M + H]^+^); Figure S8: Fragmentations pattern and positive ion mode high-resolution ESI MS/MS spectrum for Lichenysin (**7**) (isoform of **6**) (*m*/*z* 1035.7063 [M + H]^+^); Figure S9: Fragmentations pattern and positive ion mode high-resolution ESI MS/MS spectrum for Lichenysin (**8**) (*m*/*z* 1063.7366 [M + H]^+^); Figure S10: Fragmentations pattern and positive ion mode high-resolution ESI MS/MS spectrum for Lichenysin (**9**) (*m*/*z* 1025.6832 [M + H]^+^); Figure S11: Fragmentations pattern and positive ion mode high-resolution ESI MS/MS spectrum for linear Lichenysin (**10**) (*m*/*z* 1039.6990 [M + H]^+^); Figure S12: Fragmentations pattern and positive ion mode high-resolution ESI MS/MS spectrum for linear Lichenysin (**11**) (*m*/*z* 1053.7147 [M + H]^+^); Figure S13: Fragmentations pattern and positive ion mode high-resolution ESI MS/MS spectrum for Surfactin (**12**) (*m*/*z* 1008.6618 [M + H]^+^); Figure S14: Fragmentations pattern and positive ion mode high-resolution ESI MS/MS spectrum for Surfactin (**13**) (*m*/*z* 1022.6767 [M + H]^+^); Figure S15: Fragmentations pattern and positive ion mode high-resolution ESI MS/MS spectrum for Surfactin (**14**) (*m*/*z* 1036.6906 [M + H]^+^); Figure S16: Fragmentations pattern and positive ion mode high-resolution ESI MS/MS spectrum for linear Surfactin (**15**) (*m*/*z* 1054.7003 [M + H]^+^); Figure S17: Molecular networks obtained using the Feature-Based Molecular Network workflow on GNPS (https://gnps.ucsd.edu/ProteoSAFe/status.jsp?task=de168cddecfe40248fa3ba5077705e55) (accessed on 23 May 2023); Figure S18: Chemical fractionation schema of *Streptomyces* sp. G222 crude extract; Figure S19: HPLC profile of the subfraction F9 at 210 nm; Figure S20: ^1^H-NMR spectrum of Lichenysin (**3**–**4**) (*m*/*z* 1007.6734 and 1007.6733 [M + H]^+^) (600 MHz, CD_3_OH); Figure S21: DEPTQ spectrum of Lichenysin (**3**–**4**) (*m*/*z* 1007.6734 and 1007.6733 [M + H]^+^) (600 MHz, CD_3_OH); Figure S22: COSY-NMR spectrum of Lichenysin (**3**–**4**) (*m*/*z* 1007.6734 and 1007.6733 [M + H]^+^) (600 MHz, CD_3_OH); Figure S23: TOCSY-NMR spectrum of Lichenysin (**3**–**4**) (*m*/*z* 1007.6734 and 1007.6733 [M + H]^+^) (600 MHz, CD_3_OH); Figure S24: HSQC-NMR spectrum of Lichenysin (**3**–**4**) (*m*/*z* 1007.6734 and 1007.6733 [M + H]^+^) (600 MHz, CD_3_OH); Figure S25: HMBC-NMR spectrum of Lichenysin (**3**–**4**) (*m*/*z* 1007.6734 and 1007.6733 [M + H]^+^) (600 MHz, CD_3_OH); Figure S26: ROESY-NMR spectrum of Lichenysin (**3**) (*m*/*z* 1007.6734 and 1007.6733 [M + H]^+^) (600 MHz, CD_3_OH); Figure S27: Structure of Lichenysins (**3**–**4**) (*m*/*z* 1007.6734 and 1007.6733 [M + H]^+^, respectively); Figure S28: ^1^H-NMR spectrum of Lichenysin (**5**) (*m*/*z* 1021.6896 [M + H]^+^) (600 MHz, CD_3_OH); Figure S29: DEPTQ spectrum of Lichenysin (**5a** and **5b**) (*m*/*z* 1021.6896 [M + H]^+^) (600 MHz, CD_3_OH); Figure S30: COSY-NMR spectrum of Lichenysin (**5a** and **5b**) (*m*/*z* 1021.6896 [M + H]^+^) (600 MHz, CD_3_OH); Figure S31: TOCSY-NMR spectrum of Lichenysin (**5a** and **5b**) (*m*/*z* 1021.6896 [M + H]^+^) (600 MHz, CD_3_OH); Figure S32: HSQC-NMR spectrum of Lichenysin (**5a** and **5b**) (*m*/*z* 1021.6896 [M + H]^+^) (600 MHz, CD_3_OH); Figure S33: HMBC-NMR spectrum of Lichenysin (**5a** and **5b**) (*m*/*z* 1021.6896 [M + H]^+^) (600 MHz, CD_3_OH); Figure S34: ROESY-NMR spectrum of Lichenysin (**5a** and **5b**) (*m*/*z* 1021.6896 [M + H]^+^) (600 MHz, CD_3_OH); Figure S35: Structure of Lichenysins (**5a** and **5b**) (*m*/*z* 1021.6896 [M + H]^+^); Figure S36: ^1^H-NMR spectrum of Lichenysin (**6**–**7**) (*m*/*z* 1035.7063 [M + H]^+^) (600 MHz, CD_3_OH); Figure S37: DEPTQ spectrum of Lichenysin (**6**–**7**) (*m*/*z* 1035.7063 [M + H]^+^) (600 MHz, CD_3_OH); Figure S38: COSY-NMR spectrum of Lichenysin (**6**–**7**) (*m*/*z* 1035.7063 [M + H]^+^) (600 MHz, CD_3_OH); Figure S39: TOCSY-NMR spectrum of Lichenysin (**6**–**7**) (*m*/*z* 1035.7063 [M + H]^+^) (600 MHz, CD_3_OH); Figure S40: HSQC-NMR spectrum of Lichenysin (**6**–**7**) (*m*/*z* 1035.7063 [M + H]^+^) (600 MHz, CD_3_OH); Figure S41: HMBC-NMR spectrum of Lichenysin (**6**–**7**) (*m*/*z* 1035.7063 [M + H]^+^) (600 MHz, CD_3_OH); Figure S42: ROESY-NMR spectrum of Lichenysin (**6**–**7**) (*m*/*z* 1035.7063 [M + H]^+^) (600 MHz, CD_3_OH); Figure S43: Structure of Lichenysins (**6**–**7**) (*m*/*z* 1035.7063 [M + H]^+^); Figure S44: Marfey’s analysis in positive ion mode high-resolution ESI mass spectrum; Figure S45: Biofilm-forming activity against *Pseudomonas aeruginosa* MUC-N1; Figure S46: (A) ATP release in *S. aureus* MRSA exhibited by compounds (**3**–**4**) and (**6**–**7**) as determined using ATP efflux assay. Squalamine (100 µg.mL^−1^) was the positive control and water was the negative control. Compounds were tested at a final concentration of 100 µg.mL^−1^, and results are reported as relative luminescence unit. (B) Bacterial growth inhibition exhibited by (**3**–**4**) and (**6**–**7**) against *S. aureus* MRSA (CF-Marseille) with different concentrations. Positive control was bacteria only and negative control was media only. Table S1: Product ion spectra data for Lichenysin (**1**) (*m*/*z* 979. 6434 [M + H]^+^); Table S2: Product ion spectra data for Lichenysin (**2**) (*m*/*z* 993.6581 [M + H]^+^); Table S3: Product ion spectra data for Lichenysin (**3**) (*m*/*z* 1007.6734 [M + H]^+^); Table S4: Product ion spectra data for Lichenysin (**4**) (isoform of **3**) (*m*/*z* 1007.6733 [M + H]^+^); Table S5: Product ion spectra data for Lichenysin (**5**) (*m*/*z* 1021.6896 [M + H]^+^); Table S6: Product ion spectra data for Lichenysin (**6**) (*m*/*z* 1035.7063 [M + H]^+^); Table S7: Product ion spectra data Lichenysin (**7**) (isoform of **6**) (*m*/*z* 1035.7063 [M + H]^+^); Table S8: Product ion spectra data Lichenysin (**8**) (*m*/*z* 1063.7366 [M + H]^+^); Table S9: Product ion spectra data for Lichenysin (**9**) (*m*/*z* 1025.6832 [M + H]^+^); Table S10: Product ion spectra data for Linear Lichenysin (**10**) (*m*/*z* 1039.6990 [M + H]^+^); Table S11: Product ion spectra data for Linear Lichenysin (**11**) (*m*/*z* 1053.7147 [M + H]^+^); Table S12: Product ion spectra data for Surfactin (**12**) (*m*/*z* 1008.6618 [M + H]^+^); Table S13: Product ion spectra data for Surfactin (**13**) (*m*/*z* 1022.6767 [M + H]^+^); Table S14: Product ion spectra data for Surfactin (**14**) (*m*/*z* 1036.6906 [M + H]^+^); Table S15: Product ion spectra data for Linear Surfactin (**15**) (*m*/*z* 1054.7003 [M + H]^+^); Table S16: ^1^H, ^13^C, HMBC, and ROESY NMR data of Lichenysin (**3**–**4**) (*m*/*z* 1007.6734 and 1007.6733 [M + H]^+^) (600 MHz, CD_3_OH), isoform *ante-iso* and *iso* (0.8:0.2); Table S17: ^1^H, ^13^C, HMBC, and ROESY NMR data of Lichenysin (**5a** and **5b**) (*m*/*z* 1021.6896 [M + H]^+^) (600 MHz, CD_3_OH) isoform: *iso* and *n* (0.5:0.5); Table S18: ^1^H, ^13^C, HMBC, and ROESY NMR data of Lichenysin (**6**–**7**) (*m*/*z* 1035.7063 [M + H]^+^) (600 MHz, CD_3_OH) isoform: *ante*-*iso* and *iso* (0.2:0.8).
