# Supplementary material for: Computational Methods Reveal a Series of Cyclic and Linear Lichenysins and Surfactins from the Vietnamese Marine Sediment-Derived Streptomyces Strain G222
Source: Molecules. 2024 Mar 24;29(7):1458. doi: 10.3390/molecules29071458 (PMC11012875; doi:10.3390/molecules29071458)
Supplement: Supplementary file 1 [file molecules-29-01458-s001.zip › molecules-2903930-supplementary.pdf]

molecules

Supplementary materials

Computational Methods Reveal a Series of Cyclic and Linear Lichenysins and Surfactins from the Vietnamese Marine Sediment-Derived *Streptomyces* Strain G222

Andrea Castaldi [1], Bich Ngan Truong [2], Quyen Thi Vu [2,†], Thi Hong Minh Le [2,†], Arul Marie [1], Gaël Le Pennec [3], Florent Rouvier [4], Jean-Michel Brunel [4], Arlette Longeon [1], Van Cuong Pham [2], Thi Mai Huong Doan [2,*], and Marie-Lise Bourguet-Kondracki [1,*]

[1] Molécules de Communication et Adaptation des Microorganismes, UMR 7245 CNRS, Muséum National d'Histoire Naturelle, 57 rue Cuvier (CP54), 75005 Paris, France
[2] Institute of Marine Biochemistry (IMBC), Vietnam Academy of Science and Technology (VAST), 18 Hoang Quoc Viet, Caugiay, Hanoi 100000, Vietnam
[3] Laboratoire de Biotechnologie et Chimie Marines, Université Bretagne Sud, EMR CNRS 6076, IUEM, 56100 Lorient, France
[4] UMR MD1 "Membranes et Cibles Thérapeutiques", U1261 INSERM, Aix-Marseille Université, Faculté de Pharmacie, 27 Bd Jean Moulin, CEDEX 5, 13385 Marseille, France
* Correspondence: huongdm@imbc.vast.vn (D.T.M.H.); marie-lise.bourguet@mnhn.fr (M.-L.B.-K.)
† The authors contributed equally to the study

Figure S1: A) The molecular networking obtained through the LC-MS/MS analysis of the extract of G222. B) Zoom view of the discriminant cluster of the lipopeptides.

Figure S2: Fragmentations pattern and positive ion mode high-resolution ESI MS/MS spectrum for Lichenysin (**1**) (*m/z* 979.6434 $[M + H]^+$).

Figure S3: Fragmentations pattern and positive ion mode high-resolution ESI MS/MS spectrum for Lichenysin (**2**) (*m/z* 993.6581 $[M + H]^+$)

Figure S4: Fragmentations pattern and positive ion mode high-resolution ESI MS/MS spectrum for Lichenysin (**3**) (*m/z* 1007.6734 $[M + H]^+$)

Figure S5: Fragmentations pattern and positive ion mode high-resolution ESI MS/MS spectrum for Lichenysin (**4**) (isoform of **3**) (*m/z* 1007.6733 $[M + H]^+$)

Figure S6: Fragmentations pattern and positive ion mode high-resolution ESI MS/MS spectrum for Lichenysin (**5**) (*m/z* 1021.6896 $[M + H]^+$)

Figure S7: Fragmentations pattern and positive ion mode high-resolution ESI MS/MS spectrum for Lichenysin (**6**) (*m/z* 1035.7063 $[M + H]^+$)

Figure S8: Fragmentations pattern and positive ion mode high-resolution ESI MS/MS spectrum for Lichenysin (**7**) (isoform of **6**) (*m/z* 1035.7063 $[M + H]^+$)

Figure S9: Fragmentations pattern and positive ion mode high-resolution ESI MS/MS spectrum for Lichenysin (**8**) (*m/z* 1063.7366 $[M + H]^+$)

Figure S10: Fragmentations pattern and positive ion mode high-resolution ESI MS/MS spectrum for Lichenysin (**9**) (*m/z* 1025.6832 $[M + H]^+$).

Figure S11: Fragmentations pattern and positive ion mode high-resolution ESI MS/MS spectrum for linear Lichenysin (**10**) (*m/z* 1039.6990 $[M + H]^+$)

Figure S12: Fragmentations pattern and positive ion mode high-resolution ESI MS/MS spectrum for linear Lichenysin (**11**) (*m/z* 1053.7147 $[M + H]^+$)

Figure S13: Fragmentations pattern and positive ion mode high-resolution ESI MS/MS spectrum for Surfactin (**12**) (*m/z* 1008.6618 $[M + H]^+$)
Figure S14: Fragmentations pattern and positive ion mode high-resolution ESI MS/MS spectrum for Surfactin (**13**) (*m/z* 1022.6767 $[M + H]^+$)
Figure S15: Fragmentations pattern and positive ion mode high-resolution ESI MS/MS spectrum for Surfactin (**14**) (*m/z* 1036.6906 $[M + H]^+$)
Figure S16: Fragmentations pattern and positive ion mode high-resolution ESI MS/MS spectrum for linear Surfactin (**15**) (*m/z* 1054.7003 $[M + H]^+$)
Figure S17: Molecular networks obtained using the Feature-Based Molecular Network workflow on GNPS (https://gnps.ucsd.edu/ProteoSAFe/status.jsp?task=de168cddecfe40248fa3ba5077705e55) (accessed on 23 May 2023).
Figure S18: Chemical fractionation schema of *Streptomyces* sp. G222 crude extract.
Figure S19: HPLC profile of the subfraction F9 at 210 nm.
Figure S20: ^{1}H-NMR spectrum of Lichenysin (**3**-**4**) (*m/z* 1007.6734 and 1007.6733 $[M + H]^+$) (600 MHz, CD_3OH).
Figure S21: DEPTQ spectrum of Lichenysin (**3**-**4**) (*m/z* 1007.6734 and 1007.6733 $[M + H]^+$) (600 MHz, CD_3OH).
Figure S22: COSY-NMR spectrum of Lichenysin (**3**-**4**) (*m/z* 1007.6734 and 1007.6733 $[M + H]^+$) (600 MHz, CD_3OH).
Figure S23: TOCSY-NMR spectrum of Lichenysin (**3**-**4**) (*m/z* 1007.6734 and 1007.6733 $[M + H]^+$) (600 MHz, CD_3OH).
Figure S24: HSQC-NMR spectrum of Lichenysin (**3**-**4**) (*m/z* 1007.6734 and 1007.6733 $[M + H]^+$) (600 MHz, CD_3OH)
Figure S25: HMBC-NMR spectrum of Lichenysin (**3**-**4**) (*m/z* 1007.6734 and 1007.6733 $[M + H]^+$) (600 MHz, CD_3OH).
Figure S26: ROESY-NMR spectrum of Lichenysin (**3**-**4**) (*m/z* 1007.6734 and 1007.6733 $[M + H]^+$) (600 MHz, CD_3OH).
Figure S27: Structure of Lichenysins (**3**-**4**) (*m/z* 1007.6734 and 1007.6733 $[M + H]^+$; respectively).
Figure S28: ^{1}H-NMR spectrum of Lichenysin (**5a** and **5b**) (*m/z* 1021.6896 $[M + H]^+$) (600 MHz, CD_3OH).
Figure S29: DEPTQ spectrum of Lichenysin (**5a** and **5b**) (*m/z* 1021.6896 $[M + H]^+$) (600 MHz, CD_3OH).
Figure S30: COSY-NMR spectrum of Lichenysin (**5a** and **5b**) (*m/z* 1021.6896 $[M + H]^+$) (600 MHz, CD_3OH).
Figure S31: TOCSY-NMR spectrum of Lichenysin (**5a** and **5b**) (*m/z* 1021.6896 $[M + H]^+$) (600 MHz, CD_3OH).
Figure S32: HSQC-NMR spectrum of Lichenysin (**5a** and **5b**) (*m/z* 1021.6896 $[M + H]^+$) (600 MHz, CD_3OH).
Figure S33: HMBC-NMR spectrum of Lichenysin (**5a** and **5b**) (*m/z* 1021.6896 $[M + H]^+$) (600 MHz, CD_3OH).
Figure S34: ROESY-NMR spectrum of Lichenysin (**5a** and **5b**) (*m/z* 1021.6896 $[M + H]^+$) (600 MHz, CD_3OH).
Figure S35: Structure of Lichenysins (**5a** and **5b**) (*m/z* 1021.6896 $[M + H]^+$).
Figure S36: ^{1}H-NMR spectrum of Lichenysin (**6**-**7**) (*m/z* 1035.7063 $[M + H]^+$) (600 MHz, CD_3OH).
Figure S37: DEPTQ spectrum of Lichenysin (**6**-**7**) (*m/z* 1035.7063 $[M + H]^+$) (600 MHz, CD_3OH).
Figure S38: COSY-NMR spectrum of Lichenysin (**6**-**7**) (*m/z* 1035.7063 $[M + H]^+$) (600 MHz, CD_3OH).
Figure S39: TOCSY-NMR spectrum of Lichenysin (**6**-**7**) (*m/z* 1035.7063 $[M + H]^+$) (600 MHz, CD_3OH).
Figure S40: HSQC-NMR spectrum of Lichenysin (**6**-**7**) (*m/z* 1035.7063 $[M + H]^+$) (600 MHz, CD_3OH).
Figure S41: HMBC-NMR spectrum of Lichenysin (**6**-**7**) (*m/z* 1035.7063 $[M + H]^+$) (600 MHz, CD_3OH).
Figure S42: ROESY-NMR spectrum of Lichenysin (**6**-**7**) (*m/z* 1035.7063 $[M + H]^+$) (600 MHz, CD_3OH).
Figure S43: Structure of Lichenysins (**6**-**7**) (*m/z* 1035.7063 $[M + H]^+$).
Figure S44: Marfey's analysis in positive ion mode high-resolution ESI mass spectrum
Figure S45: Biofilm-forming activity against *Pseudomonas aeruginosa* MUC-N1.
Figure S46: A) ATP release in *S. aureus* MRSA exhibited by compounds (**3**-**4**) and (**6**-**7**) as determined using ATP efflux assay. Squalamine (100 $\mu g.mL^{-1}$) was the positive control and water was the negative control. Compounds were tested at a final concentration of 100 $\mu g.mL^{-1}$, and results are reported as relative luminescence unit. B) Bacterial growth

inhibition exhibited by (**3**-**4**) and (**6**-**7**) against *S. aureus* MRSA (CF-Marseille) with different concentrations. Positive control was bacteria only and negative control was media only.

Table S1: Product ion spectra data for Lichenysin (**1**) (*m/z* 979.6434 $[M + H]^+$)
Table S2: Product ion spectra data for Lichenysin (**2**) (*m/z* 993.6581 $[M + H]^+$)
Table S3: Product ion spectra data for Lichenysin (**3**) (*m/z* 1007.6734 $[M + H]^+$)
Table S4: Product ion spectra data for Lichenysin (**4**) (isoform of **3**) (*m/z* 1007.6733 $[M + H]^+$)
Table S5: Product ion spectra data for Lichenysin (**5**) (*m/z* 1021.6896 $[M + H]^+$)
Table S6: Product ion spectra data for Lichenysin (**6**) (*m/z* 1035.7063 $[M + H]^+$)
Table S7: Product ion spectra data Lichenysin (**7**) (isoform of **6**) (*m/z* 1035.7063 $[M + H]^+$)
Table S8: Product ion spectra data Lichenysin (**8**) (*m/z* 1063.7366 $[M + H]^+$)
Table S9: Product ion spectra data for Lichenysin (**9**) (*m/z* 1025.6832 $[M + H]^+$)
Table S10: Product ion spectra data for Linear Lichenysin (**10**) (*m/z* 1039.6990 $[M + H]^+$)
Table S11: Product ion spectra data for Linear Lichenysin (**11**) (*m/z* 1053.7147 $[M + H]^+$)
Table S12: Product ion spectra data for Surfactin (**12**) (*m/z* 1008.6618 $[M + H]^+$)
Table S13: Product ion spectra data for Surfactin (**13**) (*m/z* 1022.6767 $[M + H]^+$)
Table S14: Product ion spectra data for Surfactin (**14**) (*m/z* 1036.6906 $[M + H]^+$)
Table S15: Product ion spectra data for Linear Surfactin (**15**) (*m/z* 1054.7003 $[M + H]^+$)
Table S16. ^{1}H, ^{13}C, HMBC, and ROESY NMR data of Lichenysin (**3**-**4**) (*m/z* 1007.6734 and 1007.6733 $[M + H]^+$) (600 MHz, CD_3OH), isoform *ante-iso* and *iso* (0.8:0.2).
Table S17. ^{1}H, ^{13}C, HMBC, and ROESY NMR data of Lichenysin (**5a** and **5b**) (*m/z* 1021.6896 $[M + H]^+$) (600 MHz, CD_3OH) isoform: *iso* and *n* (0.5:0.5).
Table S18. ^{1}H, ^{13}C, HMBC, and ROESY NMR data of Lichenysin (**6-7**) (*m/z* 1035.7063 $[M + H]^+$) (600 MHz, CD_3OH) isoform: *ante-iso* and *iso* (0.2:0.8).

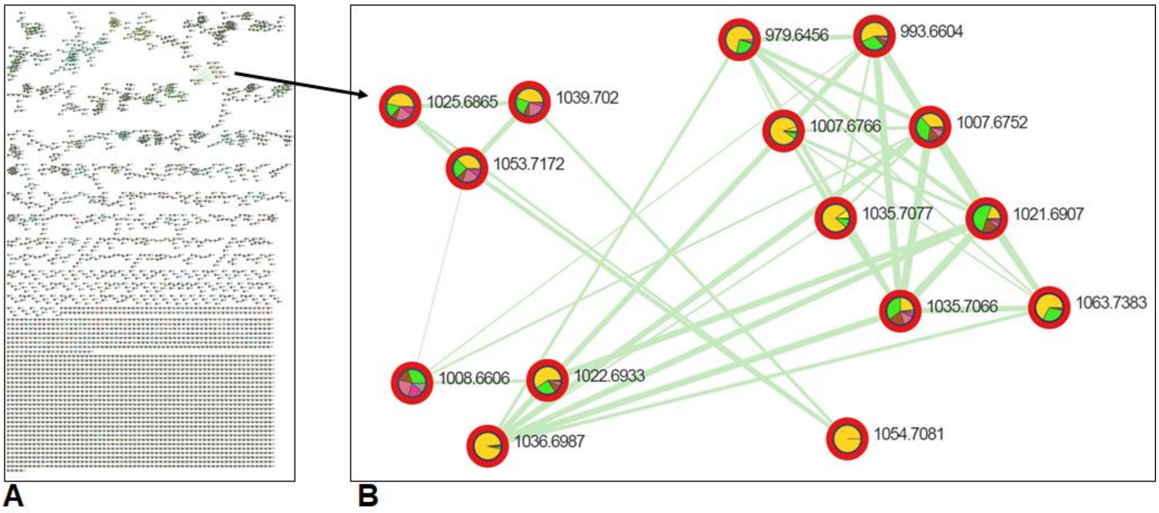

Figure S1. A) The molecular networking obtained through the LC-MS/MS analysis of the extract of G222. B) Zoom view of the discriminant cluster of the lipopeptides.

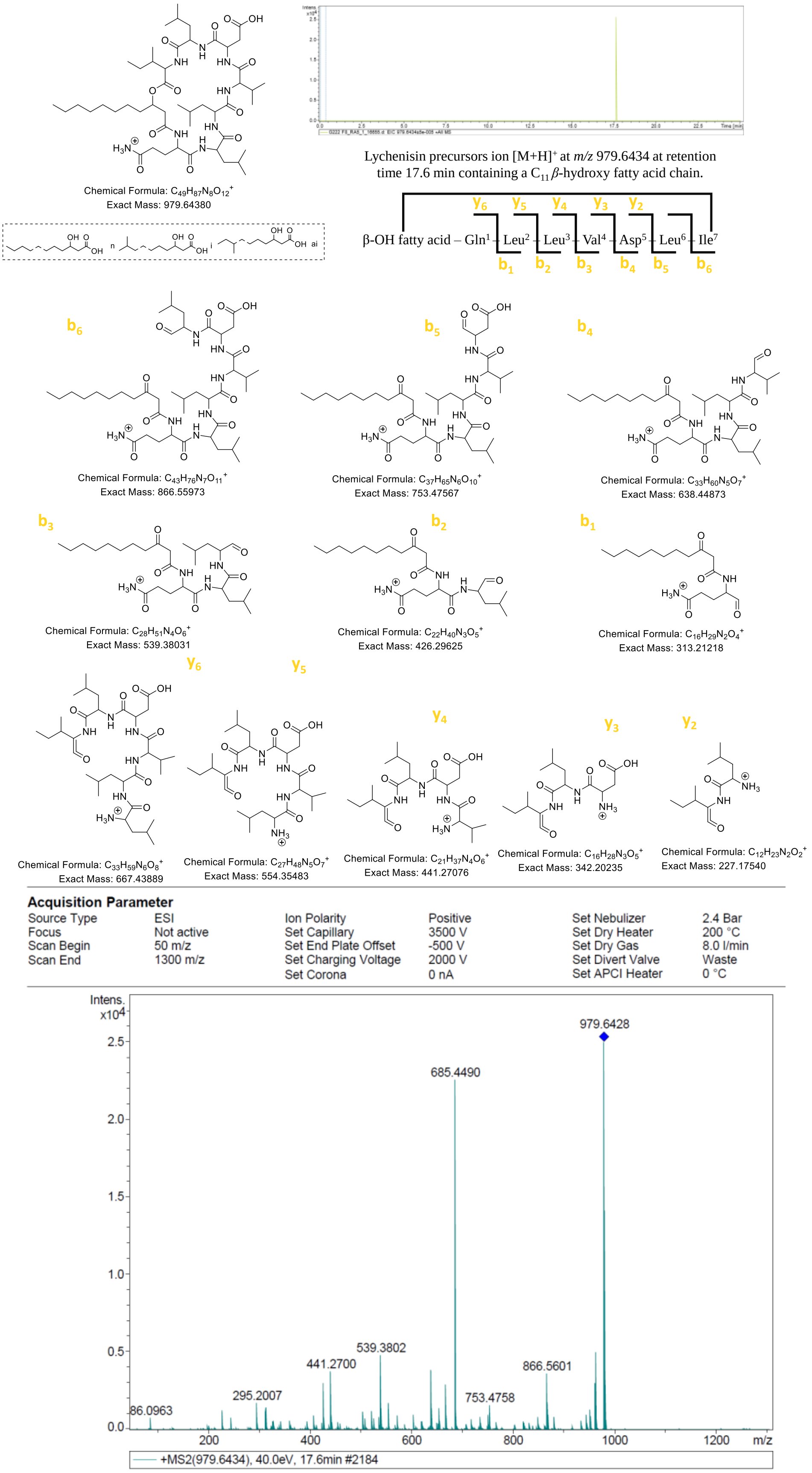

Figure S2: Fragmentations pattern and positive ion mode high-resolution ESI MS/MS spectrum for Lichenysin (**1**) (*m/z* 979.6501 [M + H]+).

Table S1. Product ion spectra data for Lichenysin (**1**) (*m/z* 979.6434 [M + H]$^+$).

Product ion Assignment	(*m/z*)	Error, pm	Molecular Formula
β-OH fatty acid – Gln^1 – Leu^2 – Leu^3 – Val^4 – Asp^5 – Leu^6 – Ile^7	979.6434	0.4	$C_{49}H_{87}N_8O_{12}$
b_6	866.5601	0.4	$C_{43}H_{76}N_7O_{11}$
b_5	753.4758	-0.2	$C_{37}H_{65}N_6O_{10}$
b_4	638.4484	0.5	$C_{33}H_{60}N_5O_7$
b_3	539.3802	0.3	$C_{28}H_{51}N_4O_6$
b_2	426.2960	0.6	$C_{22}H_{40}N_3O_5$
b_1	313.2116	1.8	$C_{16}H_{29}N_2O_4$
y_6	667.4395	-0.9	$C_{33}H_{59}N_8O_6$
y_5	554.3550	-0.3	$C_{27}H_{48}N_5O_7$
y_4	441.2700	1.8	$C_{21}H_{37}N_4O_6$
y_3	342.2022	0.4	$C_{16}H_{32}N_5O_3$
y_2	227.1753	0.7	$C_{12}H_{23}N_2O_2$

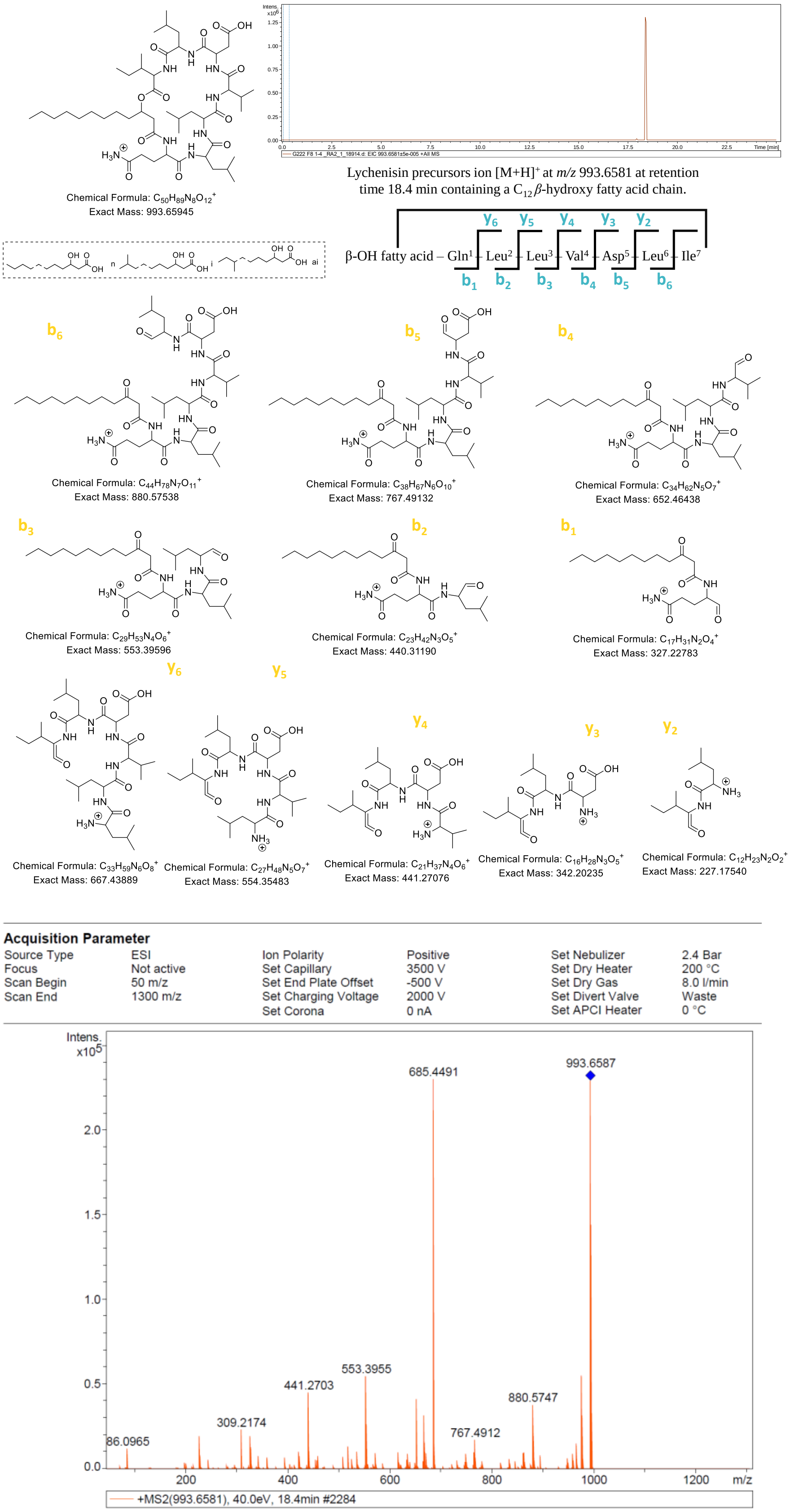

Figure S3: Fragmentations pattern and positive ion mode high-resolution ESI MS/MS spectrum for Lichenysin (**2**) (*m/z* 993.6581 [M + H]$^{+}$)

Table S2. Product ion spectra data for Lichenysin (**2**) (*m*/*z* 993.6581 [M + H]$^+$).

Product ion Assignment	(*m*/*z*)	Error, pm	Molecular Formula
β-OH fatty acid – Gln^1 – Leu^2 – Leu^3 – Val^4 – Asp^5 – Leu^6 – Ile^7	993.6581	-1.7	$C_{50}H_{89}N_8O_{12}$
b_6	880.5754	0.8	$C_{44}H_{78}N_7O_{11}$
b_5	767.4912	0.1	$C_{38}H_{67}N_6O_{10}$
b_4	652.4641	0.4	$C_{34}H_{62}N_5O_7$
b_3	553.3955	0.9	$C_{29}H_{53}N_4O_6$
b_2	440.3115	1.0	$C_{23}H_{42}N_3O_5$
b_1	327.2278	0.5	$C_{17}H_{31}N_2O_4$
y_6	667.4386	0.4	$C_{33}H_{59}N_8O_6$
y_5	554.3545	0.6	$C_{27}H_{48}N_5O_7$
y_4	441.2703	1.0	$C_{21}H_{37}N_4O_6$
y_3	342.2020	1.1	$C_{16}H_{32}N_5O_3$
y_2	227.1754	0.1	$C_{12}H_{23}N_2O_2$

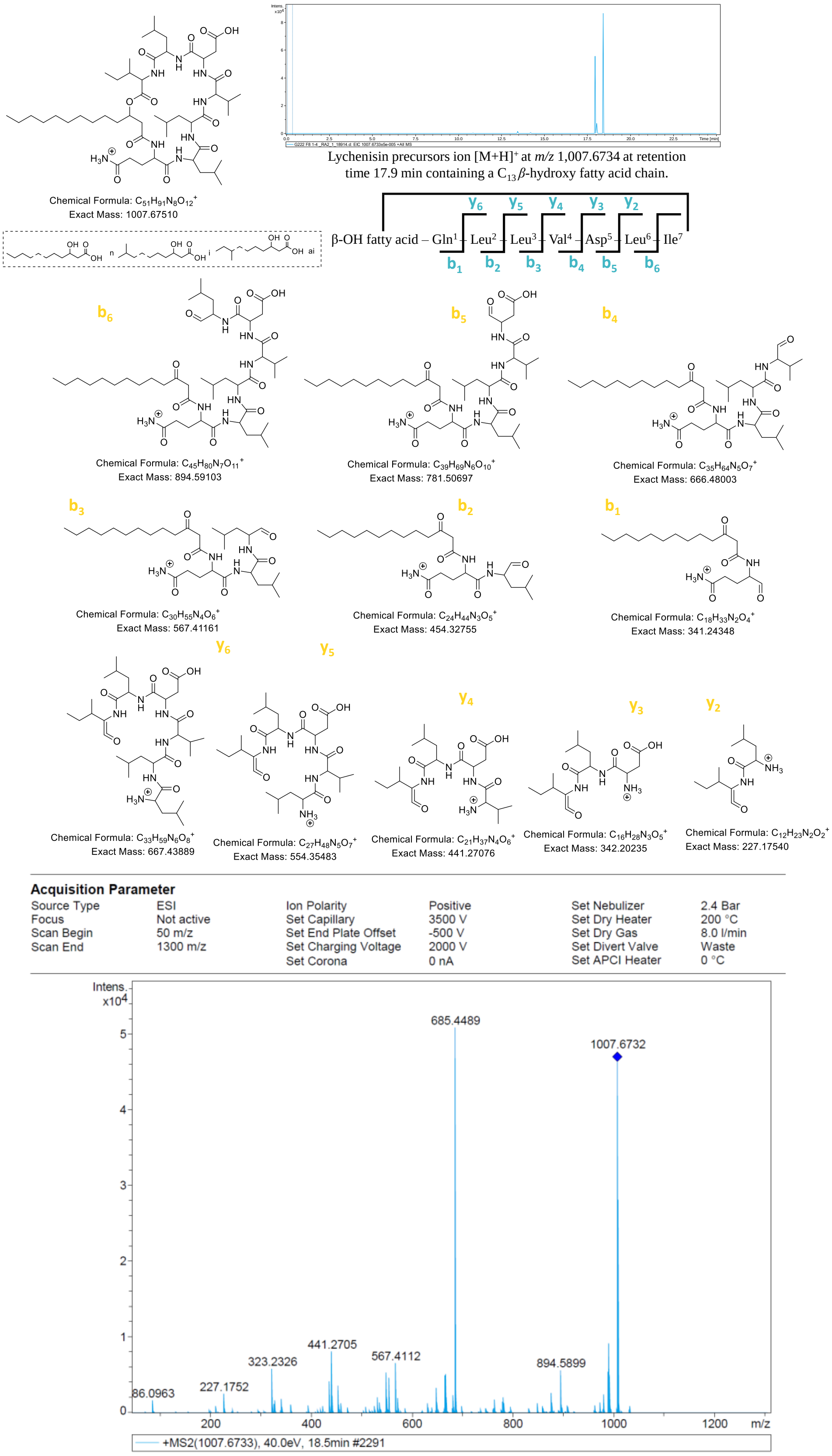

Figure S4: Fragmentations pattern and positive ion mode high-resolution ESI MS/MS spectrum for Lichenysin (**3**) (*m/z* 1007.6734 [M + H]+)

Table S3. Product ion spectra data for Lichenysin (**3**) (*m*/*z* 1007.6734 [M + H]+).

Product ion Assignment	(*m*/*z*)	Error, pm	Molecular Formula
β-OH fatty acid – Gln[1] – Leu[2] – Leu[3] – Val[4] – Asp[5] – Leu[6] – Ile[7]	1007.6734	-1.7	$C_{51}H_{91}N_8O_{12}$
b_6	894.5896	1.6	$C_{45}H_{80}N_7O_{11}$
b_5	781.5077	-1.0	$C_{39}H_{69}N_6O_{10}$
b_4	666.4787	2.0	$C_{35}H_{64}N_5O_7$
b_3	567.4111	1.0	$C_{30}H_{55}N_4O_6$
b_2	454.3267	1.8	$C_{24}H_{44}N_3O_5$
b_1	341.2429	1.6	$C_{18}H_{33}N_2O_4$
y_6	667.4393	-0.7	$C_{33}H_{59}N_8O_6$
y_5	554.3538	1.8	$C_{27}H_{48}N_5O_7$
y_4	441.2702	1.3	$C_{21}H_{37}N_4O_6$
y_3	342.2026	-0.8	$C_{16}H_{32}N_5O_3$
y_2	227.1755	0.4	$C_{12}H_{23}N_2O_2$

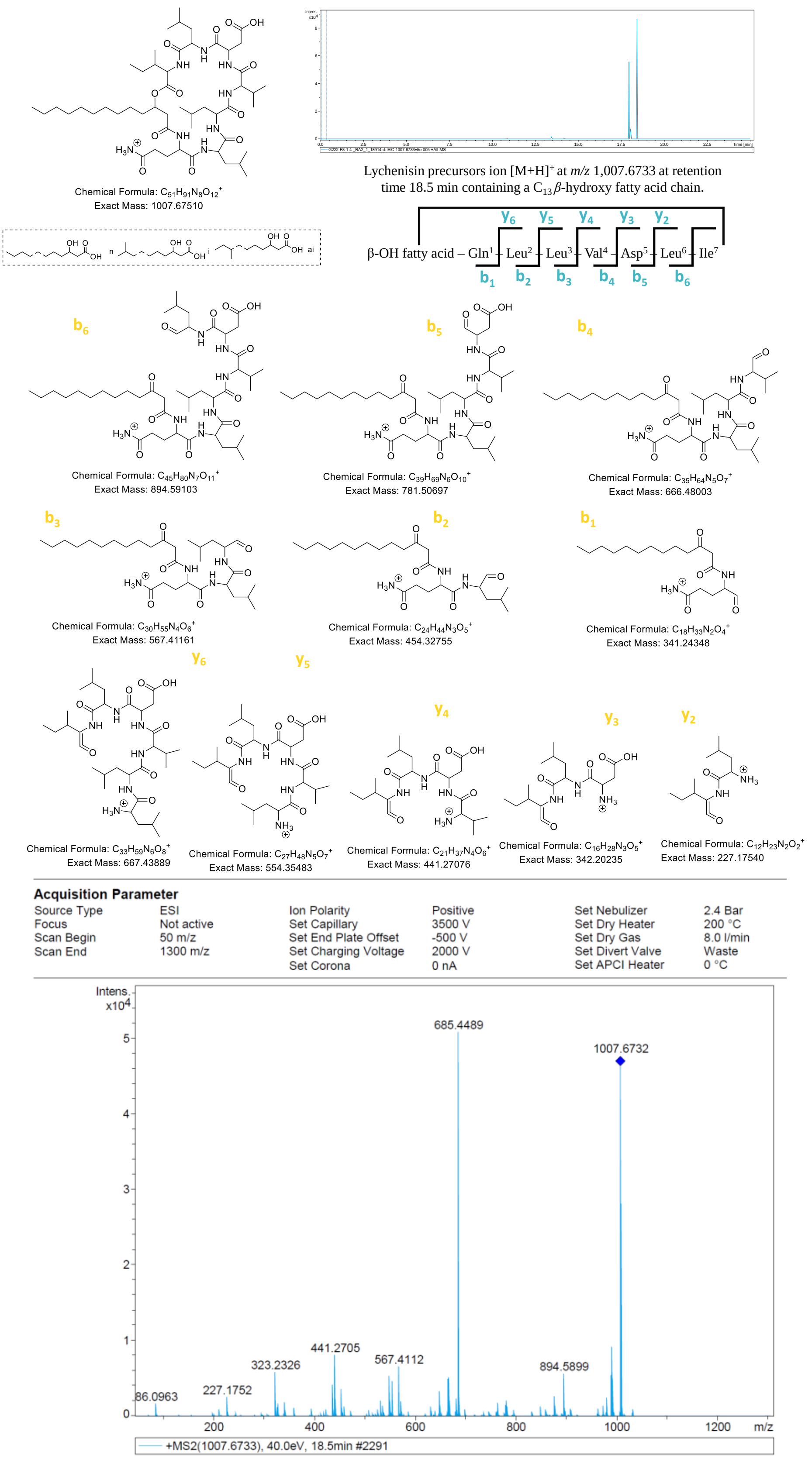

Figure S5: Fragmentations pattern and positive ion mode high-resolution ESI MS/MS spectrum for Lichenysin (**4**) (isoform of **3**) (*m/z* 1007.6733 [M + H]$^+$)

Table S4: Product ion spectra data for Lichenysin (**4**) (isoform of **3**) (*m*/*z* 1007.6733 [M + H]$^+$)

**Product ion Assignment**	**(*m*/*z*)**	**Error, pm**	**Molecular Formula**
β-OH fatty acid – Gln^1 – Leu^2 – Leu^3 – Val^4 – Asp^5 – Leu^6 – Ile^7	1007.6733	-1.8	$C_{51}H_{91}N_8O_{12}$
b_6	894.5899	2.7	$C_{45}H_{80}N_7O_{11}$
b_5	781.5060	2.9	$C_{39}H_{69}N_6O_{10}$
b_4	666.4793	1.1	$C_{35}H_{64}N_5O_7$
b_3	567.4116	0.8	$C_{30}H_{55}N_4O_6$
b_2	454.3265	2.2	$C_{24}H_{44}N_3O_5$
b_1	341.2431	1.2	$C_{18}H_{33}N_2O_4$
y_6	667.4389	0.2	$C_{33}H_{59}N_8O_6$
y_5	554.3540	-0.9	$C_{27}H_{48}N_5O_7$
y_4	441.2705	0.7	$C_{21}H_{37}N_4O_6$
y_3	342.2018	1.6	$C_{16}H_{32}N_5O_3$
y_2	227.1752	0.8	$C_{12}H_{23}N_2O_2$

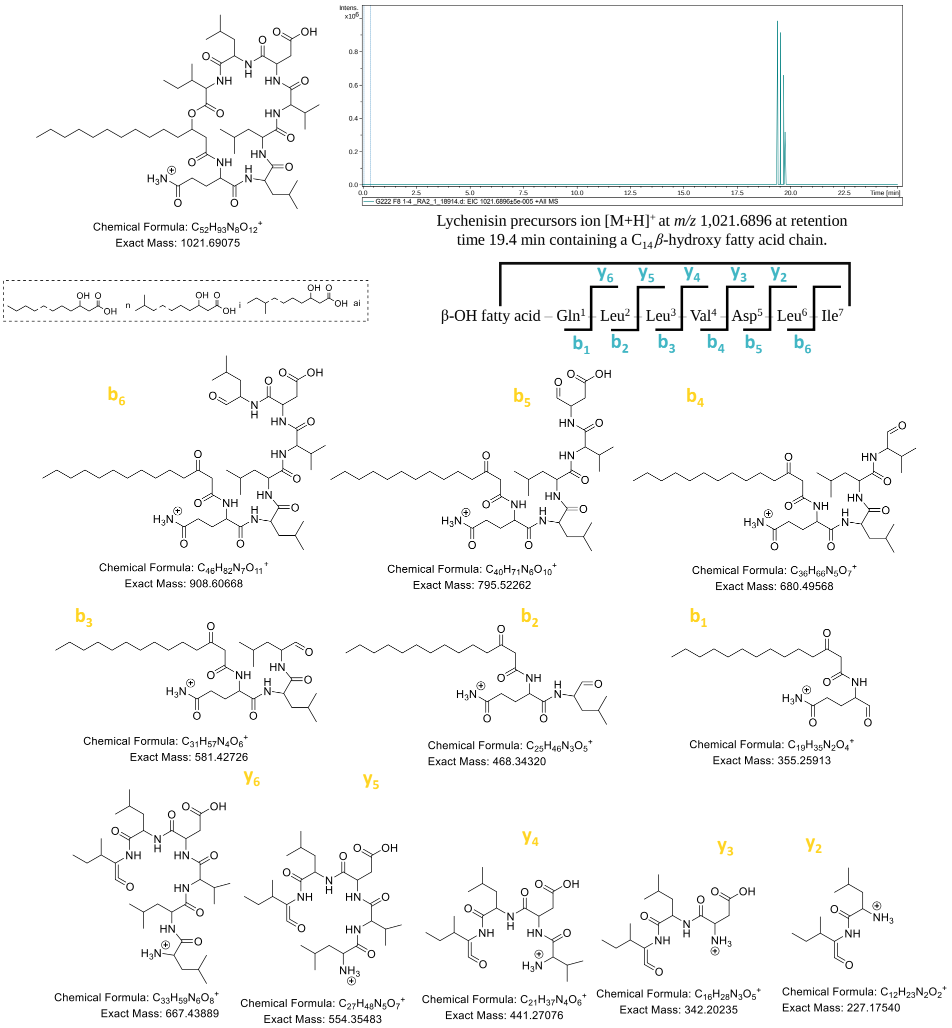

Acquisition Parameter

Source Type	ESI	Ion Polarity	Positive	Set Nebulizer	2.4 Bar
Focus	Not active	Set Capillary	3500 V	Set Dry Heater	200 °C
Scan Begin	50 m/z	Set End Plate Offset	-500 V	Set Dry Gas	8.0 l/min
Scan End	1300 m/z	Set Charging Voltage	2000 V	Set Divert Valve	Waste
		Set Corona	0 nA	Set APCI Heater	0 °C

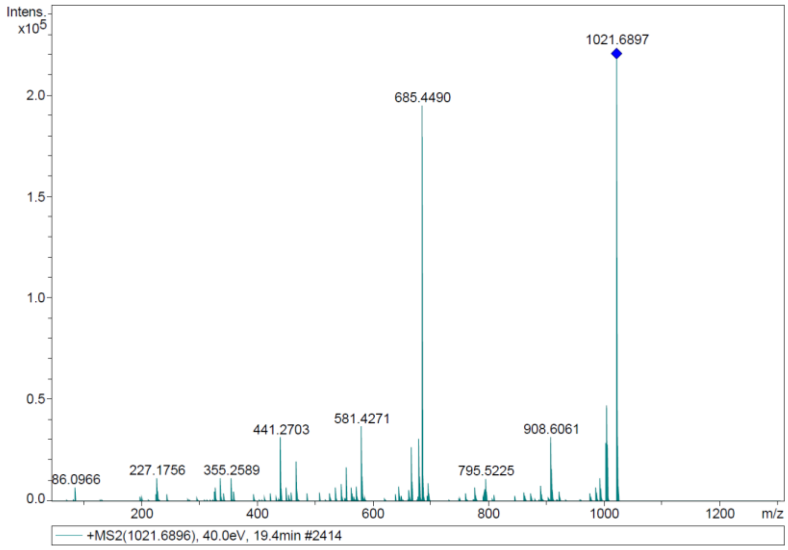

Figure S6: Fragmentations pattern and positive ion mode high-resolution ESI MS/MS spectrum for Lichenysin (**5**) (m/z 1021.6896 [M + H]$^+$)

Table S5: Product ion spectra data for Lichenysin (**5**) (*m/z* 1021.6896 [M + H]$^+$)

Product ion Assignment	(*m/z*)	Error, pm	Molecular Formula
β-OH fatty acid – Gln^1 – Leu^2 – Leu^3 – Val^4 – Asp^5 – Leu^6 – Ile^7	1021.6896	-0.01	$C_{52}H_{93}N_8O_{12}$
b_6	908.6061	0.6	$C_{46}H_{82}N_7O_{11}$
b_5	795.5225	0.2	$C_{40}H_{71}N_6O_{10}$
b_4	680.4950	1.0	$C_{36}H_{66}N_5O_7$
b_3	581.4271	0.3	$C_{31}H_{57}N_4O_6$
b_2	468.3427	1.0	$C_{25}H_{46}N_3O_5$
b_1	355.2589	0.6	$C_{19}H_{35}N_2O_4$
y_6	667.4388	0.2	$C_{33}H_{59}N_8O_6$
y_5	554.3547	0.2	$C_{27}H_{48}N_5O_7$
y_4	441.2703	1.1	$C_{21}H_{37}N_4O_6$
y_3	342.2022	0.5	$C_{16}H_{32}N_5O_3$
y_2	227.1756	-0.8	$C_{12}H_{23}N_2O_2$

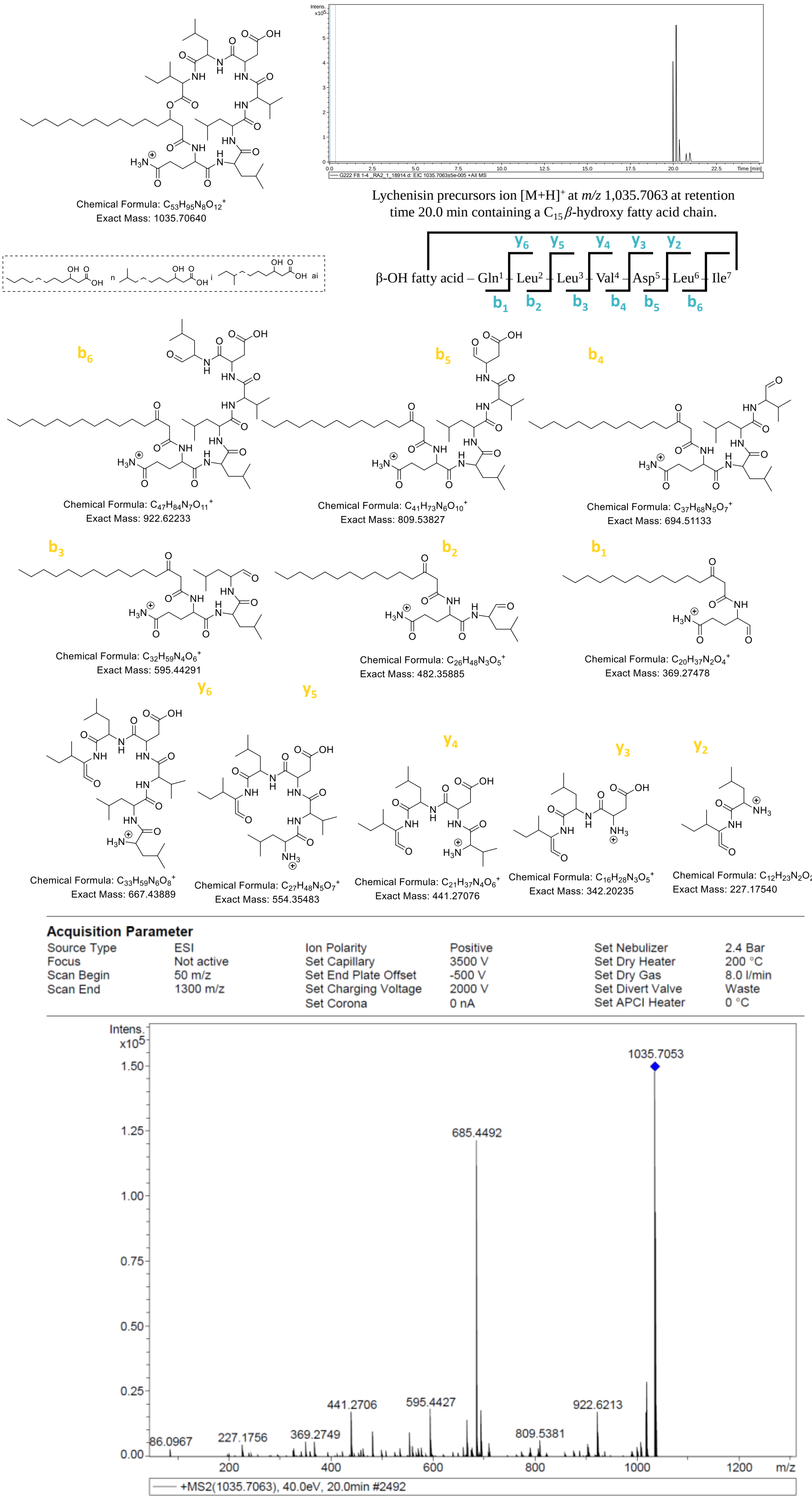

Figure S7: Fragmentations pattern and positive ion mode high-resolution ESI MS/MS spectrum for Lichenysin (**6**) (*m/z* 1035.7063 [M + H]+)

Table S6. Product ion spectra data for Lichenysin (**6**) (*m/z* 1035.7063 [M + H]+).

Product ion Assignment	(*m/z*)	Error, pm	Molecular Formula
β-OH fatty acid – Gln[1] – Leu[2] – Leu[3] – Val[4] – Asp[5] – Leu[6] – Ile[7]	1035.7063	-0.01	$C_{53}H_{95}N_8O_{12}$
b_6	922.6213	1.1	$C_{47}H_{84}N_7O_{11}$
b_5	809.5381	0.2	$C_{41}H_{73}N_6O_{10}$
b_4	694.5113	0.4	$C_{37}H_{68}N_5O_7$
b_3	595.4427	0.3	$C_{32}H_{59}N_4O_6$
b_2	482.3585	0.8	$C_{26}H_{48}N_3O_5$
b_1	369.2749	-0.2	$C_{20}H_{37}N_2O_4$
y_6	667.4391	-0.3	$C_{33}H_{59}N_8O_6$
y_5	554.3545	0.5	$C_{27}H_{48}N_5O_7$
y_4	441.2706	0.4	$C_{21}H_{37}N_4O_6$
y_3	342.2026	-0.6	$C_{16}H_{32}N_5O_3$
y_2	227.1756	-0.7	$C_{12}H_{23}N_2O_2$

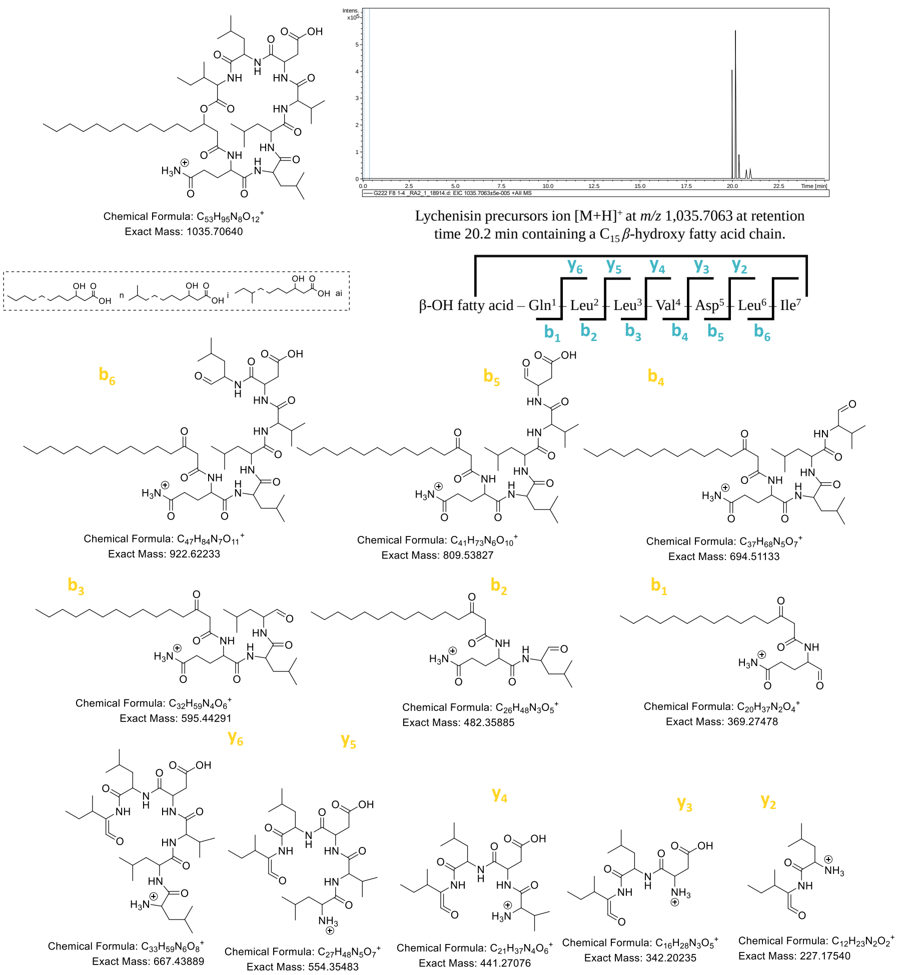

Acquisition Parameter

Source Type	ESI	Ion Polarity	Positive	Set Nebulizer	2.4 Bar
Focus	Not active	Set Capillary	3500 V	Set Dry Heater	200 °C
Scan Begin	50 m/z	Set End Plate Offset	-500 V	Set Dry Gas	8.0 l/min
Scan End	1300 m/z	Set Charging Voltage	2000 V	Set Divert Valve	Waste
		Set Corona	0 nA	Set APCI Heater	0 °C

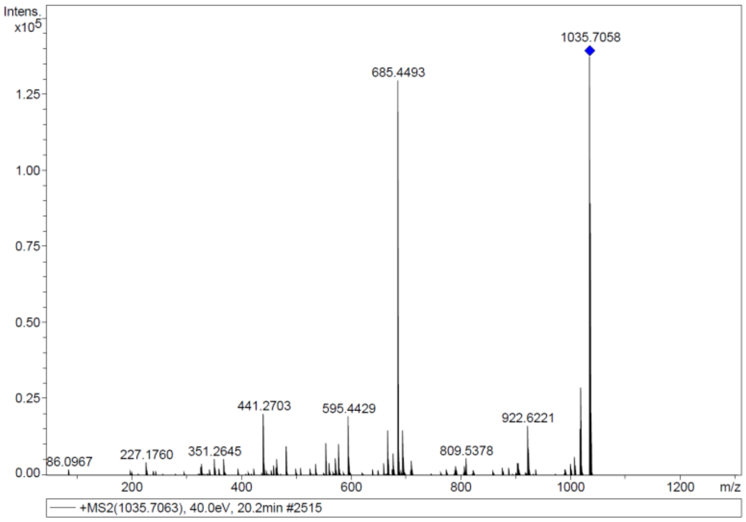

Figure S8: Fragmentations pattern and positive ion mode high-resolution ESI MS/MS spectrum for Lichenysin (**7**) (isoform of **6**) (m/z 1035.7063 $[M + H]^+$)

Table S7. Product ion spectra data Lichenysin (**7**) (isoform of **6**) (*m*/*z* 1035.7063 [M + H]$^+$).

**Product ion Assignment**	**(*m*/*z*)**	**Error, pm**	**Molecular Formula**
β-OH fatty acid – Gln^1 – Leu^2 – Leu^3 – Val^4 – Asp^5 – Leu^6 – Ile^7	1035.7063	-0.01	$C_{53}H_{95}N_8O_{12}$
b_6	922.6221	0.2	$C_{47}H_{84}N_7O_{11}$
b_5	809.5378	0.6	$C_{41}H_{73}N_6O_{10}$
b_4	694.5109	0.6	$C_{37}H_{68}N_5O_7$
b_3	595.4429	0.0	$C_{32}H_{59}N_4O_6$
b_2	482.3589	0.0	$C_{26}H_{48}N_3O_5$
b_1	369.2748	0.0	$C_{20}H_{37}N_2O_4$
y_6	667.4388	0.1	$C_{33}H_{59}N_8O_6$
y_5	554.3552	-0.6	$C_{27}H_{48}N_5O_7$
y_4	441.2703	1.0	$C_{21}H_{37}N_4O_6$
y_3	342.2025	-0.5	$C_{16}H_{32}N_5O_3$
y_2	227.1760	-2.5	$C_{12}H_{23}N_2O_2$

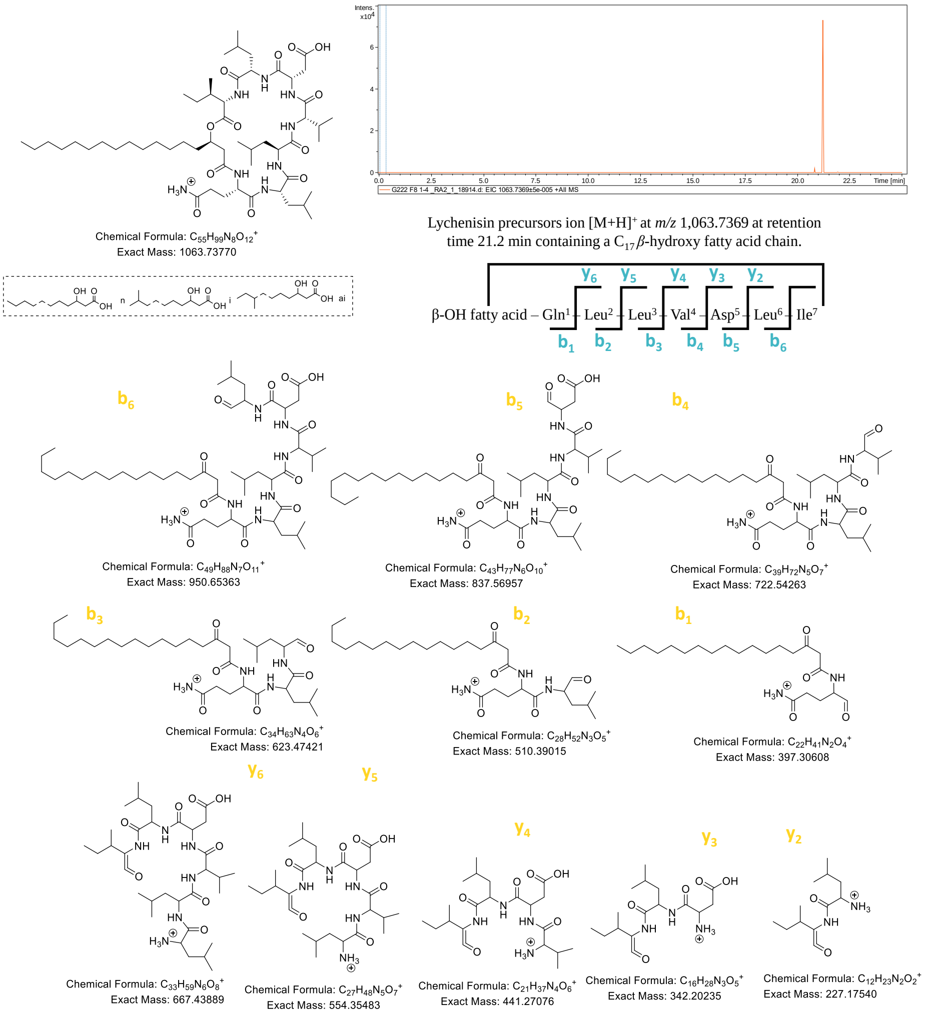

Acquisition Parameter

Source Type	ESI	Ion Polarity	Positive	Set Nebulizer	2.4 Bar
Focus	Not active	Set Capillary	3500 V	Set Dry Heater	200 °C
Scan Begin	50 m/z	Set End Plate Offset	-500 V	Set Dry Gas	8.0 l/min
Scan End	1300 m/z	Set Charging Voltage	2000 V	Set Divert Valve	Waste
		Set Corona	0 nA	Set APCI Heater	0 °C

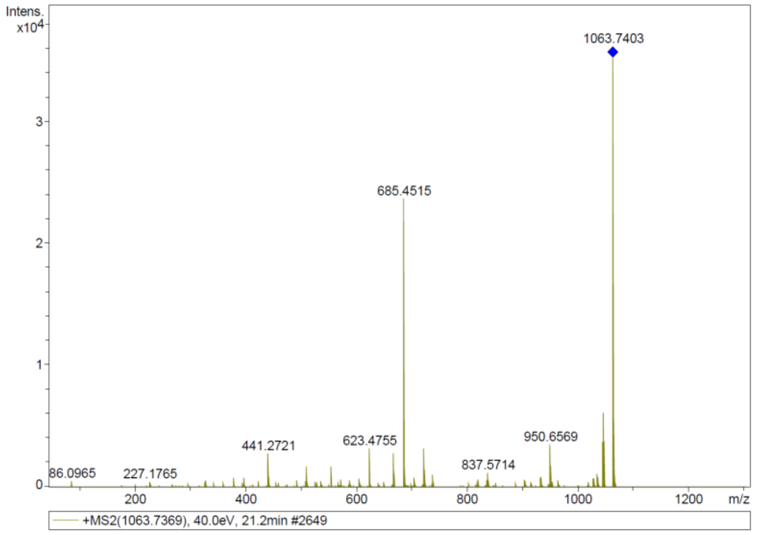

Figure S9: Fragmentations pattern and positive ion mode high-resolution ESI MS/MS spectrum for Lichenysin (**8**) (*m/z* 1063.7366 [M + H]+)

Table S8. Product ion spectra data Lichenysin (**8**) (*m*/*z* 1063.7366 [M + H]+).

Product ion Assignment	(*m*/*z*)	Error, pm	Molecular Formula
β-OH fatty acid – Gln[1] – Leu[2] – Leu[3] – Val[4] – Asp[5] – Leu[6] – Ile[7]	1063.7366	-1.0	$C_{55}H_{99}N_8O_{12}$
b_6	950.6569	-3.4	$C_{49}H_{88}N_7O_{11}$
b_5	837.5714	-0.6	$C_{43}H_{77}N_6O_{10}$
b_4	722.5448	-3.1	$C_{39}H_{72}N_5O_7$
b_3	623.4755	-2.1	$C_{34}H_{63}N_4O_6$
b_2	510.3915	-2.7	$C_{28}H_{52}N_3O_5$
b_1	397.3070	-2.4	$C_{22}H_{41}N_2O_4$
y_6	667.4414	2.2	$C_{33}H_{59}N_8O_6$
y_5	554.3564	-2.8	$C_{27}H_{48}N_5O_7$
y_4	441.2721	-2.1	$C_{21}H_{37}N_4O_6$
y_3	342.2044	-6.0	$C_{16}H_{32}N_5O_3$
y_2	227.1765	-4.8	$C_{12}H_{23}N_2O_2$

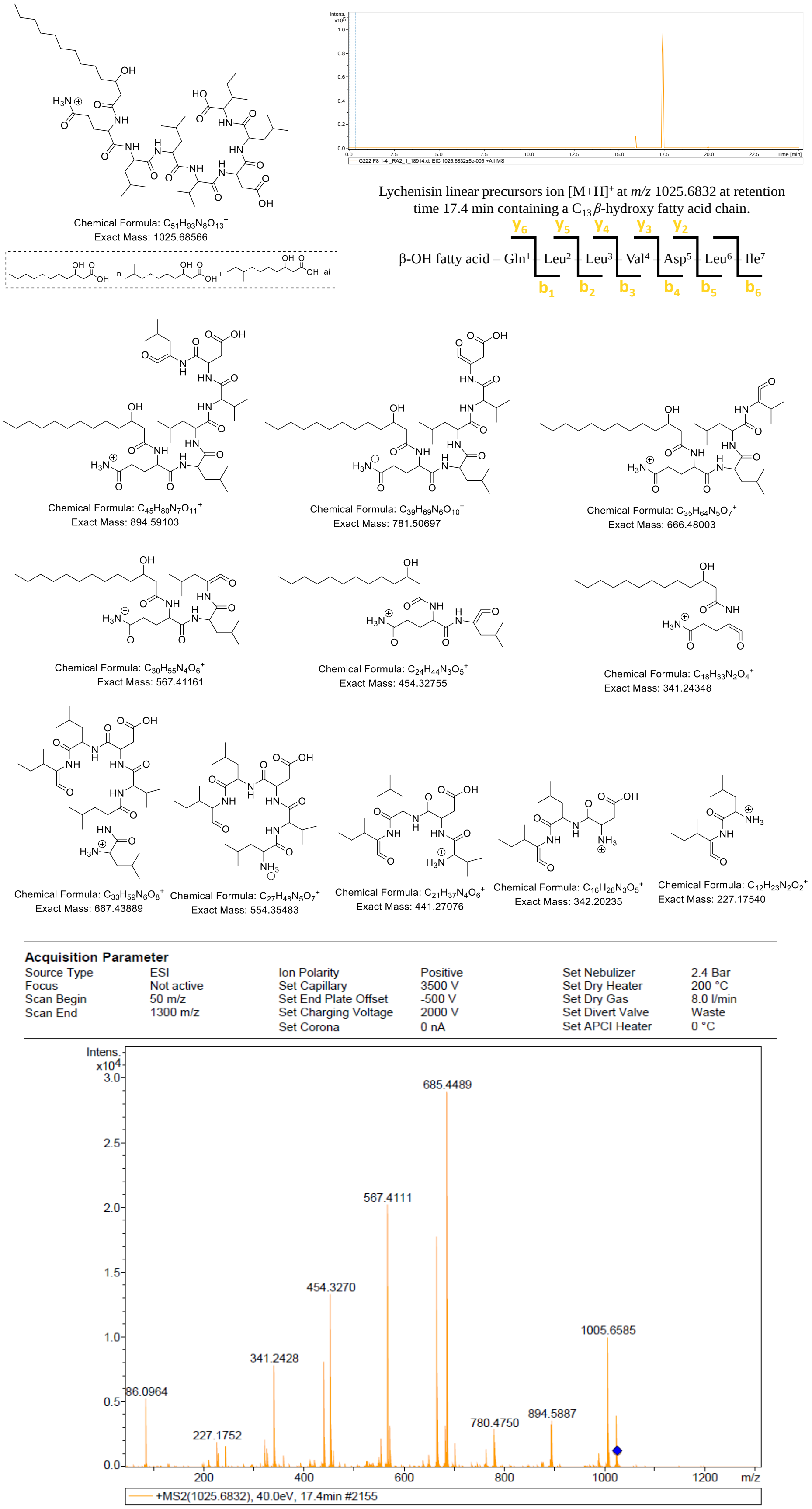

Figure S10. Fragmentations pattern and positive ion mode high-resolution ESI MS/MS spectrum for linear Lichenysin (**9**) (*m*/*z* 1025.6832 [M + H]+).

Table S9. Product ion spectra data for Lichenysin (**9**) (*m/z* 1025.6832 $[M + H]^+$).

Product ion Assignment	(*m/z*)	Error, pm	Molecular Formula
β-OH fatty acid – Gln^1 – Leu^2 – Leu^3 – Val^4 – Asp^5 – Leu^6 – Ile^7	1025.6832	-2.4	$C_{51}H_{93}N_8O_{13}$
b_6	894.5887	2.6	$C_{45}H_{80}N_7O_{11}$
b_5	781.5056	1.7	$C_{39}H_{69}N_6O_{10}$
b_4	666.4797	0.5	$C_{35}H_{64}N_5O_7$
b_3	567.4111	0.9	$C_{30}H_{55}N_4O_6$
b_2	454.3270	1.2	$C_{24}H_{44}N_3O_5$
b_1	341.2428	2.1	$C_{18}H_{33}N_2O_4$
y_6	667.4831	5.1	$C_{33}H_{59}N_8O_6$
y_5	554.3541	1.4	$C_{27}H_{48}N_5O_7$
y_4	441.2701	1.5	$C_{21}H_{37}N_4O_6$
y_3	342.2459	12.0	$C_{16}H_{32}N_5O_3$
y_2	227.1752	0.7	$C_{12}H_{23}N_2O_2$

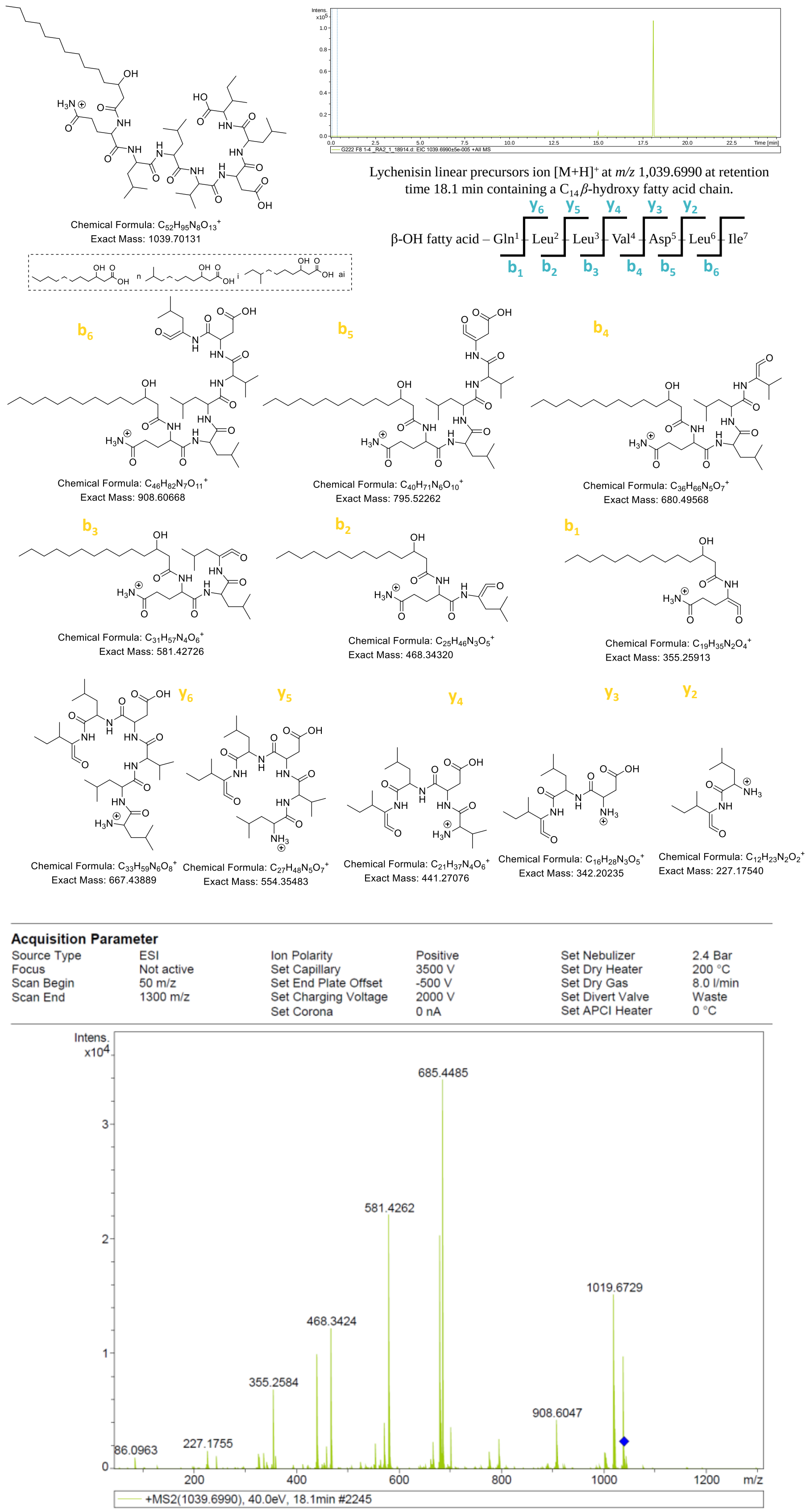

Figure S11: Fragmentations pattern and positive ion mode high-resolution ESI MS/MS spectrum for linear Lichenysin (**10**) (m/z 1039.6990 $[M + H]^+$)

Table S10. Product ion spectra data for Linear Lichenysin (**10**) (*m/z* 1039.6990 [M + H]+).

Product ion Assignment	(*m/z*)	Error, pm	Molecular Formula
β-OH fatty acid – Gln[1] – Leu[2] – Leu[3] – Val[4] – Asp[5] – Leu[6] – Ile[7]	1039.6990	-2.22	$C_{52}H_{95}N_8O_{13}$
b_6	908.6047	2.1	$C_{46}H_{82}N_7O_{11}$
b_5	795.5204	2.8	$C_{40}H_{71}N_6O_{10}$
b_4	680.4947	1.4	$C_{36}H_{66}N_5O_7$
b_3	581.4262	1.8	$C_{31}H_{57}N_4O_6$
b_2	468.3424	1.8	$C_{25}H_{46}N_3O_5$
b_1	355.2584	2.5	$C_{18}H_{33}N_2O_4$
y_6	667.4383	0.8	$C_{33}H_{59}N_8O_6$
y_5	554.3537	2.0	$C_{27}H_{48}N_5O_7$
y_4	441.2698	2.2	$C_{21}H_{37}N_4O_6$
y_3	342.2024	-0.1	$C_{19}H_{35}N_5O_3$
y_2	227.1755	-0.3	$C_{12}H_{23}N_2O_2$

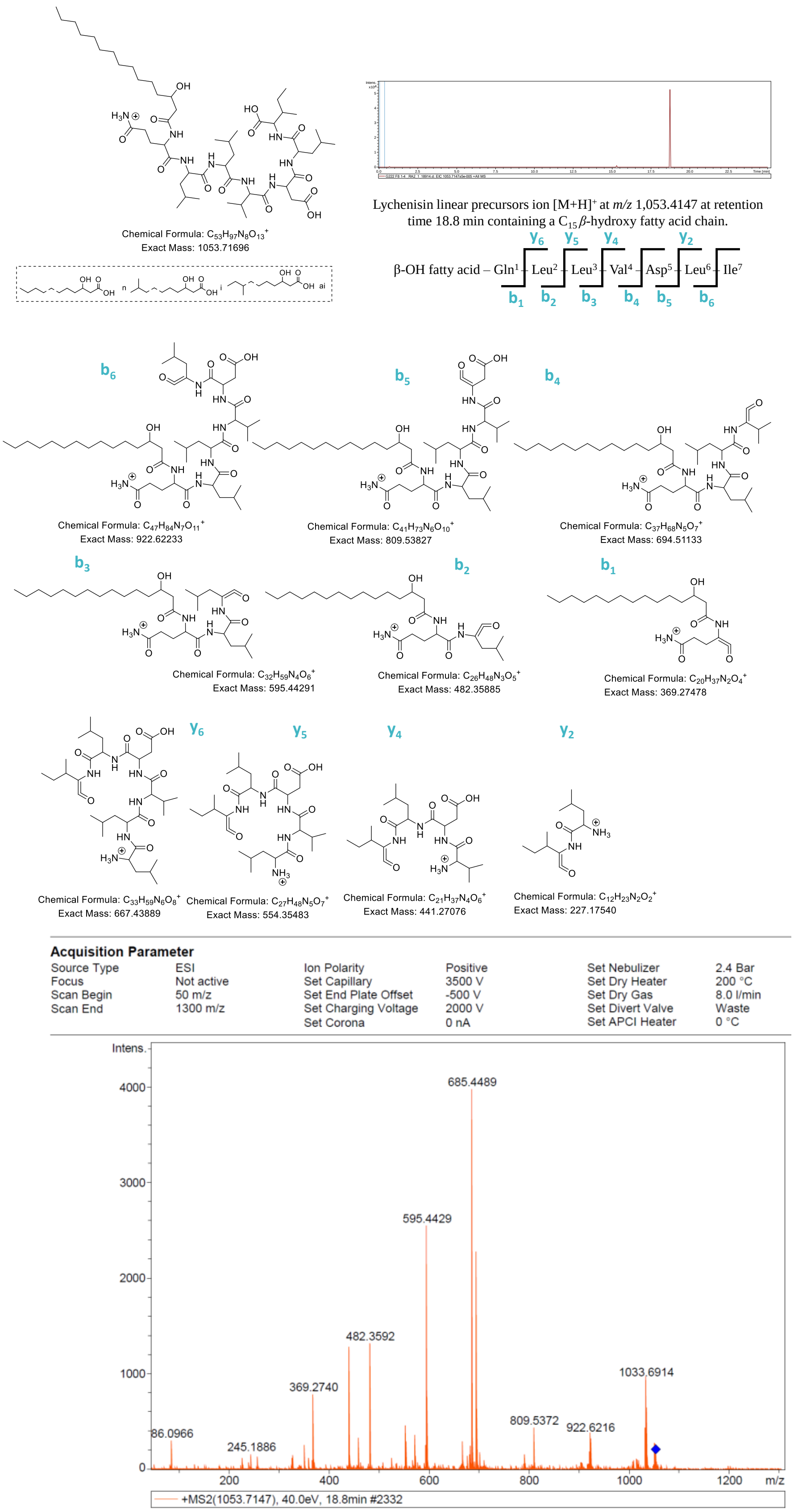

Figure S12 Fragmentations pattern and positive ion mode high-resolution ESI MS/MS spectrum for linear Lichenysin (**11**) (*m/z* 1053.7147 [M + H]$^+$)

Table S11. Product ion spectra data for Linear Lichenysin (**11**) (*m/z* 1053.7147 [M + H]+).

Product ion Assignment	(*m/z*)	Error, pm	Molecular Formula
β-OH fatty acid – Gln[1] – Leu[2] – Leu[3] – Val[4] – Asp[5] – Leu[6] – Ile[7]	1053.7147	-2.1	$C_{53}H_{97}N_8O_{13}$
b_6	922.6216	0.8	$C_{47}H_{84}N_7O_{11}$
b_5	809.5372	1.3	$C_{41}H_{73}N_6O_{10}$
b_4	694.5120	-0.9	$C_{37}H_{68}N_5O_7$
b_3	595.4429	0.0	$C_{32}H_{59}N_4O_6$
b_2	482.3592	-0.7	$C_{26}H_{48}N_3O_5$
b_1	369.2740	2.0	$C_{20}H_{37}N_2O_4$
y_6	667.4389	0.9	$C_{33}H_{59}N_8O_6$
y_5	554.3551	-0.5	$C_{27}H_{48}N_5O_7$
y_4	441.2698	2.5	$C_{21}H_{37}N_4O_6$
y_2	227.1755	0.5	$C_{12}H_{23}N_2O_2$

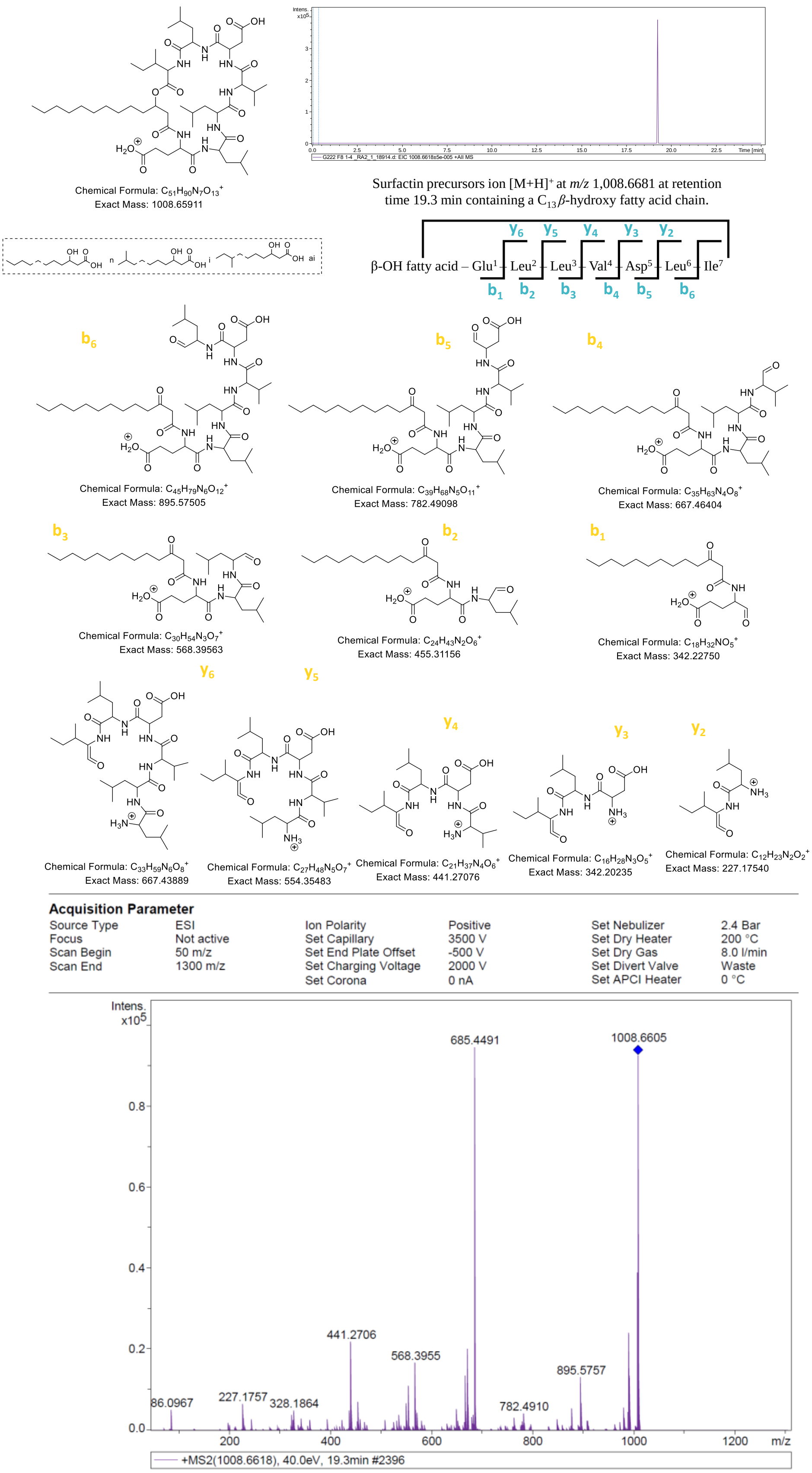

Figure S13: Fragmentations pattern and positive ion mode high-resolution ESI MS/MS spectrum for Surfactin (**12**) (m/z 1008.6618 $[M + H]^+$)

Table S12. Product ion spectra data for Surfactin (**12**) (*m*/*z* 1008.6618 [M + H]+).

Product ion Assignment	(*m*/*z*)	Error, pm	Molecular Formula
β-OH fatty acid – Glu[1] – Leu/Ile[2] – Leu[3] – Val[4] – Asp[5] – Leu[6] – Leu/Ile[7]	1008.6618	2.7	$C_{51}H_{90}N_7O_{13}$
b_6	895.5757	0.8	$C_{45}H_{79}N_6O_{12}$
b_5	782.4910	0.0	$C_{39}H_{68}N_5O_{11}$
b_4	667.4605	0.6	$C_{35}H_{63}N_4O_8$
b_3	568.3955	0.2	$C_{30}H_{54}N_3O_7$
b_2	455.3114	0.4	$C_{24}H_{43}N_2O_6$
b_1	342.2277	-0.6	$C_{18}H_{33}NO_5$
y_6	667.4412	-3.4	$C_{33}H_{59}N_8O_6$
y_5	554.3546	0.3	$C_{27}H_{48}N_5O_7$
y_4	441.2706	0.3	$C_{21}H_{37}N_4O_6$
y_3	342.2023	0.1	$C_{16}H_{32}N_5O_3$
y_2	227.0655	-1.4	$C_{12}H_{23}N_2O_2$

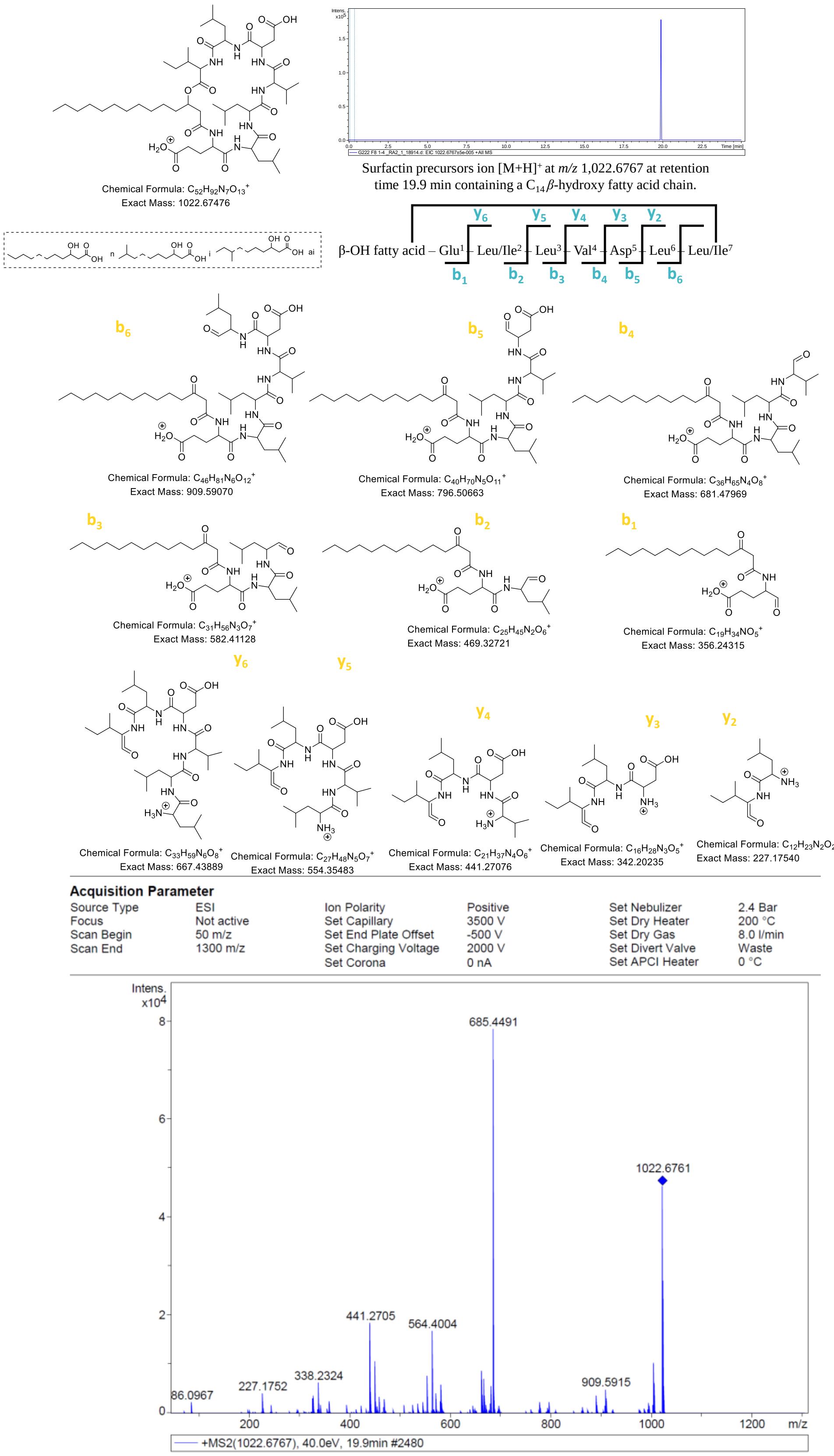

Figure S14: Fragmentations pattern and positive ion mode high-resolution ESI MS/MS spectrum for Surfactin (**13**) (*m/z* 1022.6767 [M + H]+)

Table S13. Product ion spectra data for Surfactin (**13**) (*m/z* 1022.6767 [M + H]+).

Product ion Assignment	(*m/z*)	Error, pm	Molecular Formula
β-OH fatty acid – Glu[1] – Leu/Ile[2] – Leu[3] – Val[4] – Asp[5] – Leu[6] – Leu/Ile[7]	1022.6767	1.9	$C_{52}H_{92}N_7O_{13}$
b_6	909.5915	-0.9	$C_{46}H_{81}N_6O_{12}$
b_5	796.5079	-1.6	$C_{40}H_{70}N_5O_{11}$
b_4	681.4800	1.5	$C_{36}H_{65}N_4O_8$
b_3	582.4119	-1.1	$C_{31}H_{56}N_3O_7$
b_2	469.3273	-0.1	$C_{25}H_{45}N_2O_6$
b_1	356.2429	0.6	$C_{19}H_{34}NO_5$
y_6	667.4383	0.9	$C_{33}H_{59}N_8O_6$
y_5	554.3546	0.4	$C_{27}H_{48}N_5O_7$
y_4	441.2705	0.6	$C_{21}H_{37}N_4O_6$
y_3	342.2024	-0.1	$C_{16}H_{32}N_5O_3$
y_2	227.1752	0.8	$C_{12}H_{23}N_2O_2$

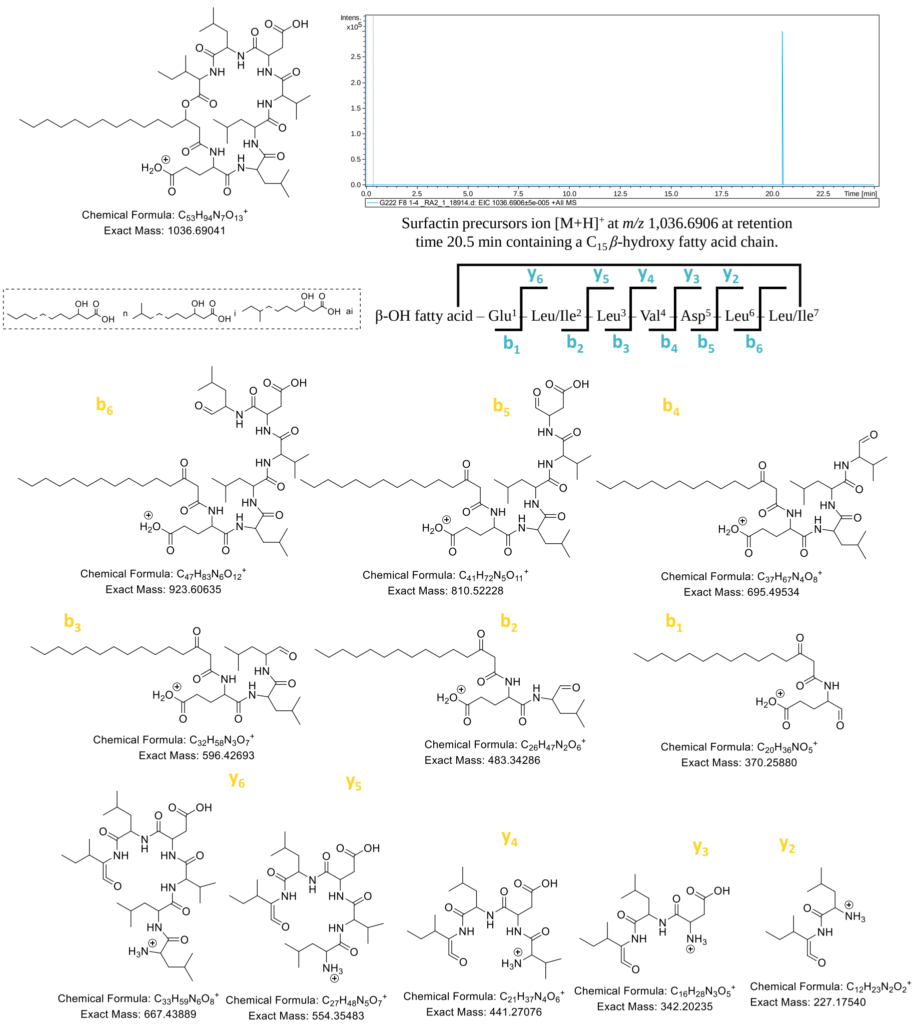

Acquisition Parameter

Source Type	ESI	Ion Polarity	Positive	Set Nebulizer	2.4 Bar
Focus	Not active	Set Capillary	3500 V	Set Dry Heater	200 °C
Scan Begin	50 m/z	Set End Plate Offset	-500 V	Set Dry Gas	8.0 l/min
Scan End	1300 m/z	Set Charging Voltage	2000 V	Set Divert Valve	Waste
		Set Corona	0 nA	Set APCI Heater	0 °C

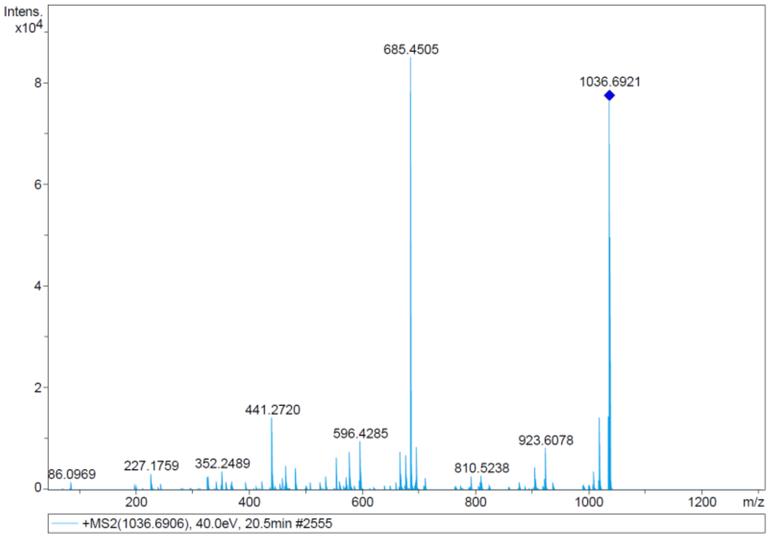

Figure S15: Fragmentations pattern and positive ion mode high-resolution ESI MS/MS spectrum for Surfactin (**14**) (*m/z* 1036.6906 $[M + H]^+$)

Table S14: Product ion spectra data for Surfactin (**14**) (*m*/*z* 1036.6906 [M + H]+)

Product ion Assignment	(*m*/*z*)	Error, pm	Molecular Formula
β-OH fatty acid – Glu[1] – Leu/Ile[2] – Leu[3] – Val[4] – Asp[5] – Leu[6] – Leu/Ile[7]	1036.6906	0.2	$C_{53}H_{94}N_7O_{13}$
b_6	923.6078	-1.6	$C_{47}H_{83}N_6O_{12}$
b_5	810.5238	-1.8	$C_{41}H_{72}N_5O_{11}$
b_4	695.4970	-2.4	$C_{37}H_{67}N_4O_8$
b_3	596.4285	-2.6	$C_{32}H_{58}N_3O_7$
b_2	483.3440	0.5	$C_{26}H_{47}N_2O_6$
b_1	370.2599	4.2	$C_{20}H_{36}NO_5$
y_6	667.4401	-1.8	$C_{33}H_{59}N_8O_6$
y_5	554.3548	-2.1	$C_{27}H_{48}N_5O_7$
y_4	441.2720	-2.9	$C_{21}H_{37}N_4O_6$
y_3	342.2033	-2.8	$C_{16}H_{32}N_5O_3$
y_2	227.1759	-2.1	$C_{12}H_{23}N_2O_2$

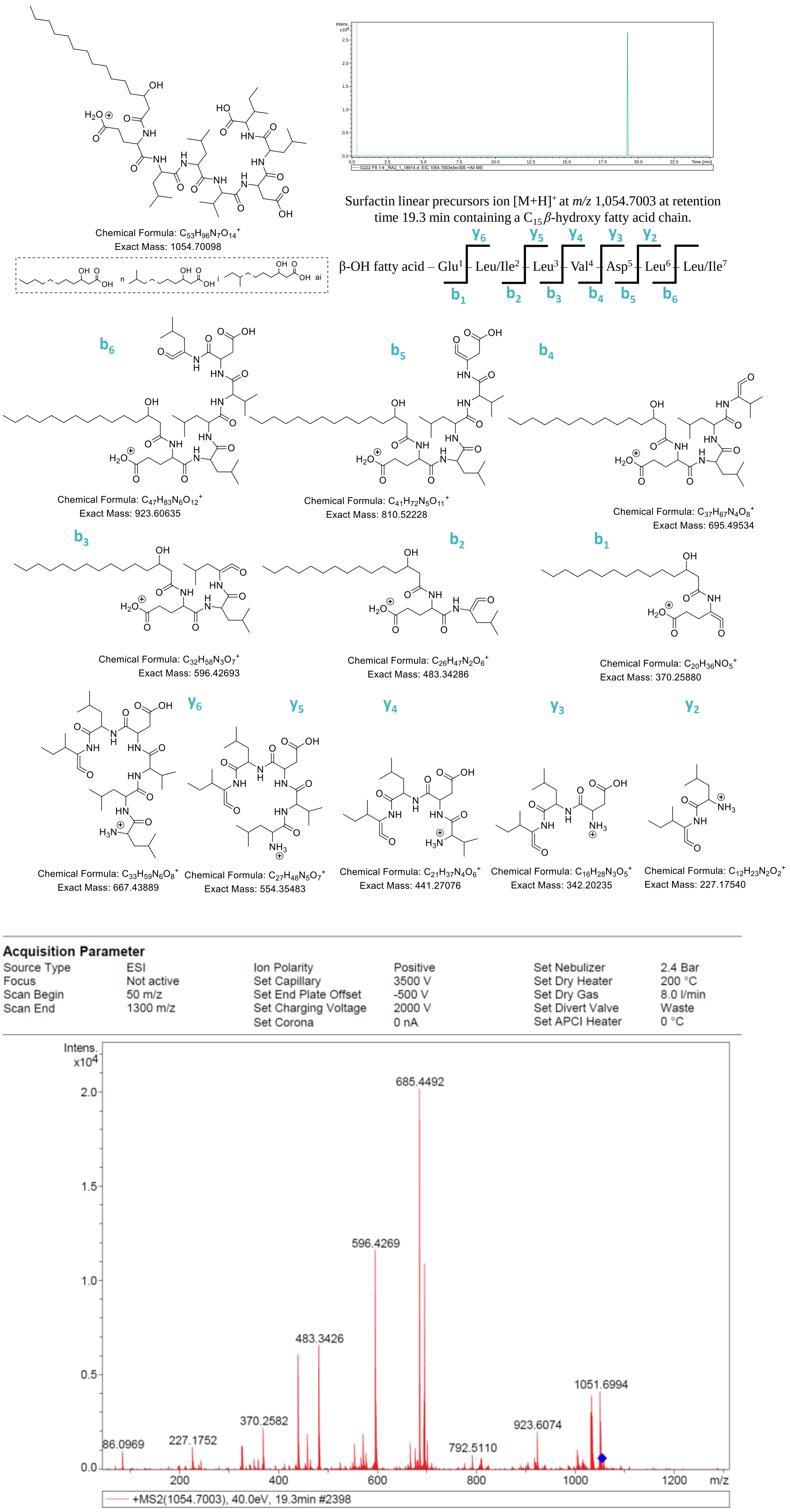

Figure S16: Fragmentations pattern and positive ion mode high-resolution ESI MS/MS spectrum for linear Surfactin (**15**) (*m/z* 1054.7003 [M + H]+)

Table S15: Product ion spectra data for Linear Surfactin (**15**) (*m/z* 1054.7003 $[M + H]^+$)

Product ion Assignment	(*m/z*)	Error, pm	Molecular Formula
β-OH fatty acid – Glu^1 – Leu/Ile^2 – Leu^3 – Val^4 – Asp^5 – Leu^6 – Leu/Ile^7	1054.7003	0.6	$C_{53}H_{96}N_7O_{14}$
b_6	923.6074	-1.2	$C_{47}H_{83}N_6O_{12}$
b_5	810.5224	-0.1	$C_{41}H_{72}N_5O_{11}$
b_4	695.4949	2.5	$C_{37}H_{67}N_4O_8$
b_3	596.4269	0.0	$C_{32}H_{58}N_3O_7$
b_2	483.3426	0.6	$C_{26}H_{47}N_2O_6$
b_1	370.2582	1.7	$C_{20}H_{36}NO_5$
y_6	667.4322	0.4	$C_{33}H_{59}N_8O_6$
y_5	554.3551	-0.6	$C_{27}H_{48}N_5O_7$
y_4	441.2708	0.0	$C_{21}H_{37}N_4O_6$
y_3	342.2024	-0.1	$C_{16}H_{28}N_3O_5$
y_2	227.1752	1.0	$C_{12}H_{23}N_2O_2$

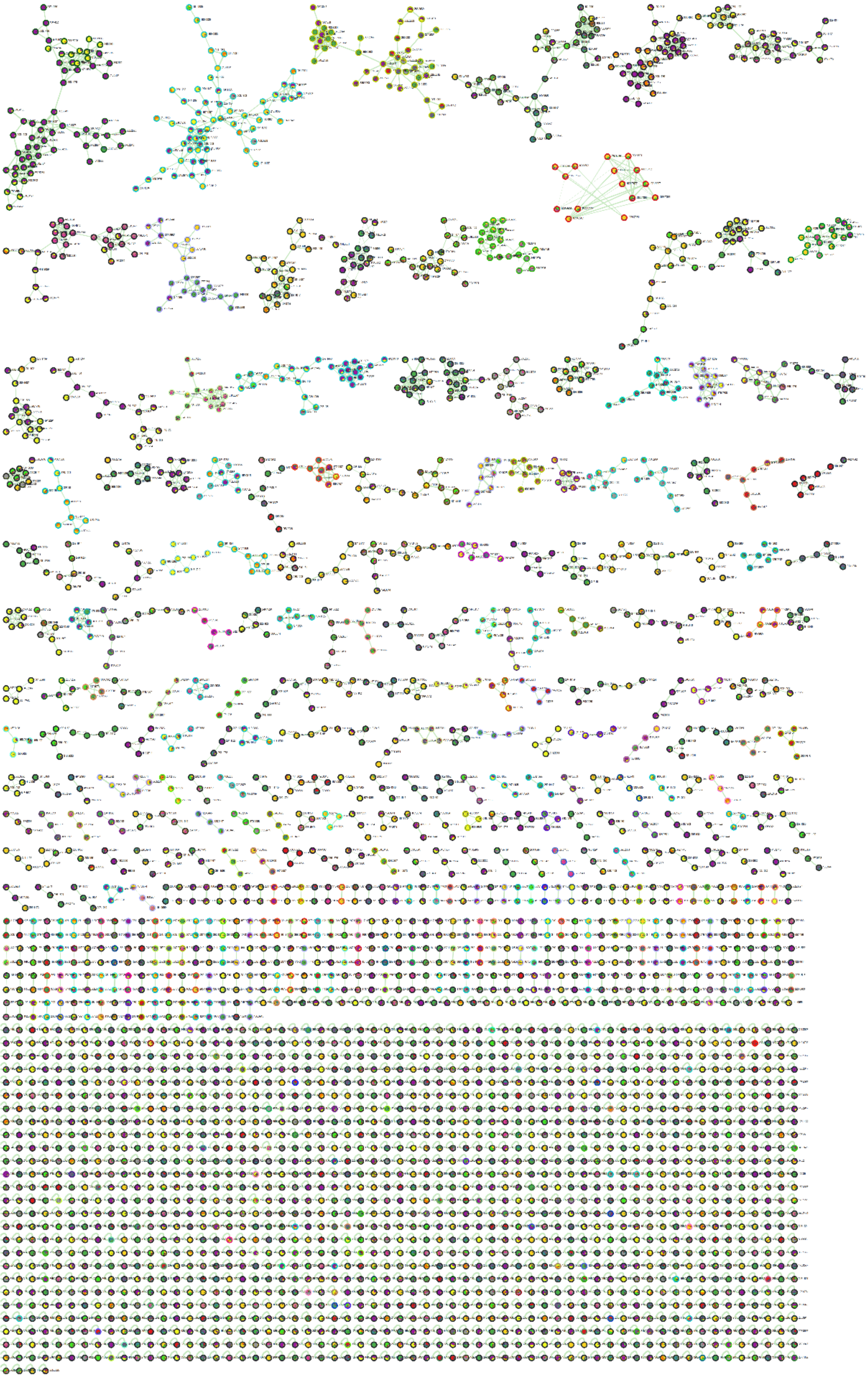

Figure S17: Molecular networks obtained using the Feature-Based Molecular Network workflow on GNPS (https://gnps.ucsd.edu/ProteoSAFe/status.jsp?task=de168cddecfe40248fa3ba5077705e55) (accessed on 23 May 2023).

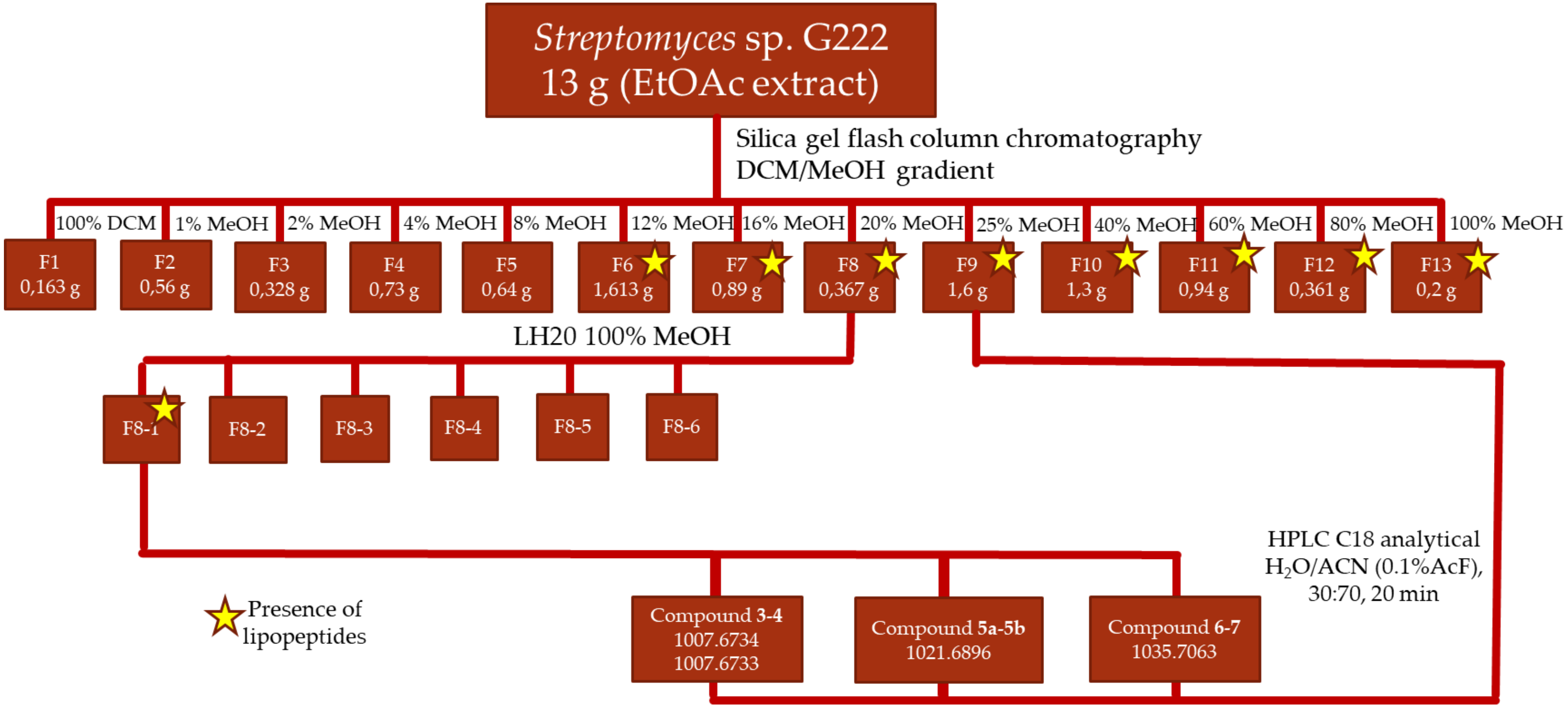

Figure S18: Chemical fractionation schema of *Streptomyces* sp. G222 crude extract.

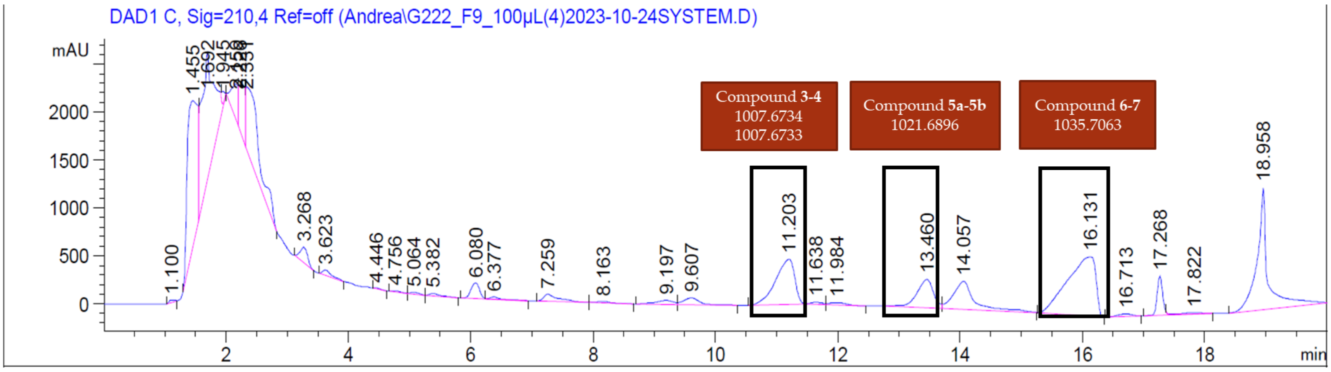

Figure S19: HPLC profile of the subfraction F9 at 210 nm.

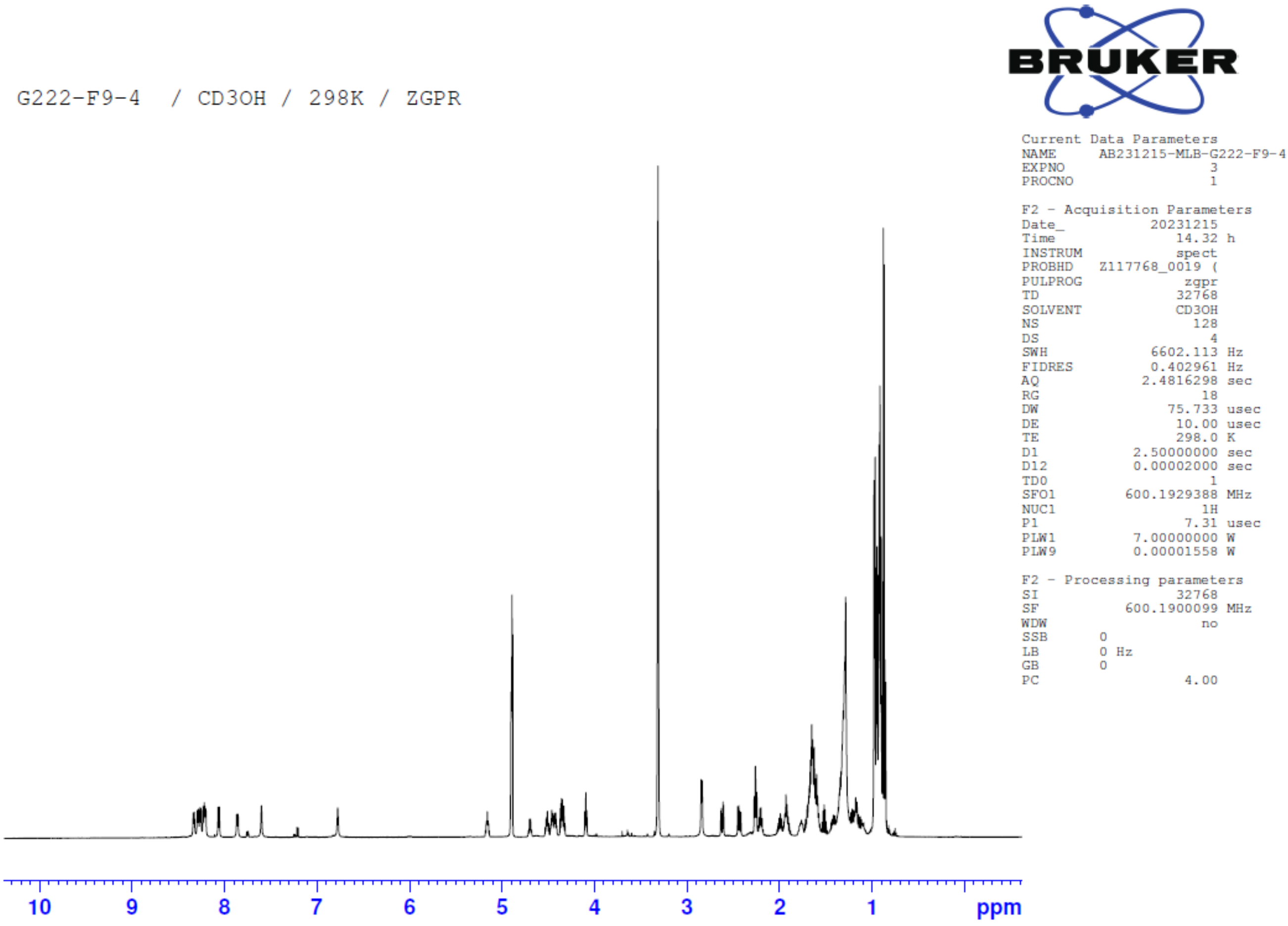

Figure S20: ^{1}H-NMR spectrum of Lichenysin (**3-4**) (*m/z* 1007.6734 and 1007.6733 $[M + H]^{+}$) (600 MHz, CD_3OH).

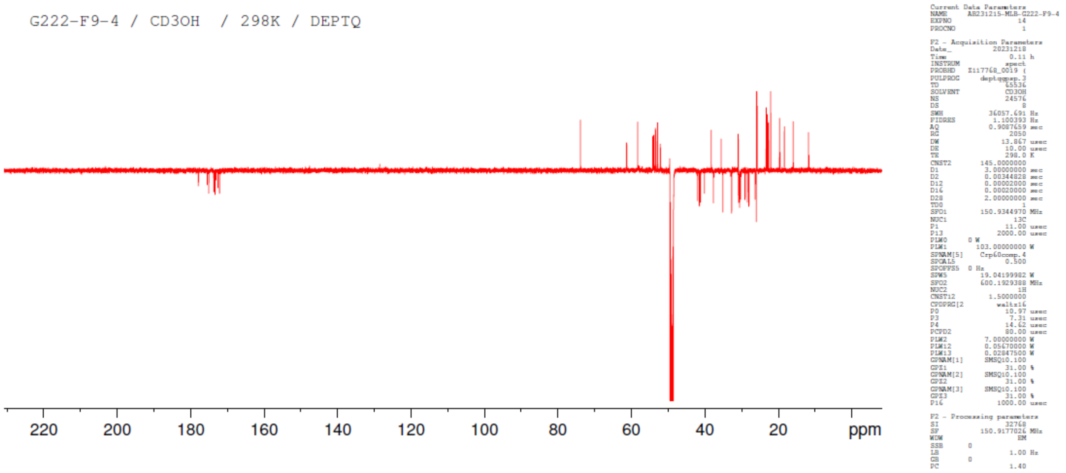

Figure S21: DEPTQ spectrum of Lichenysin (**3-4**) (*m*/*z* 1007.6734 and 1007.6733 [M + H]+) (600 MHz, CD_3OH).

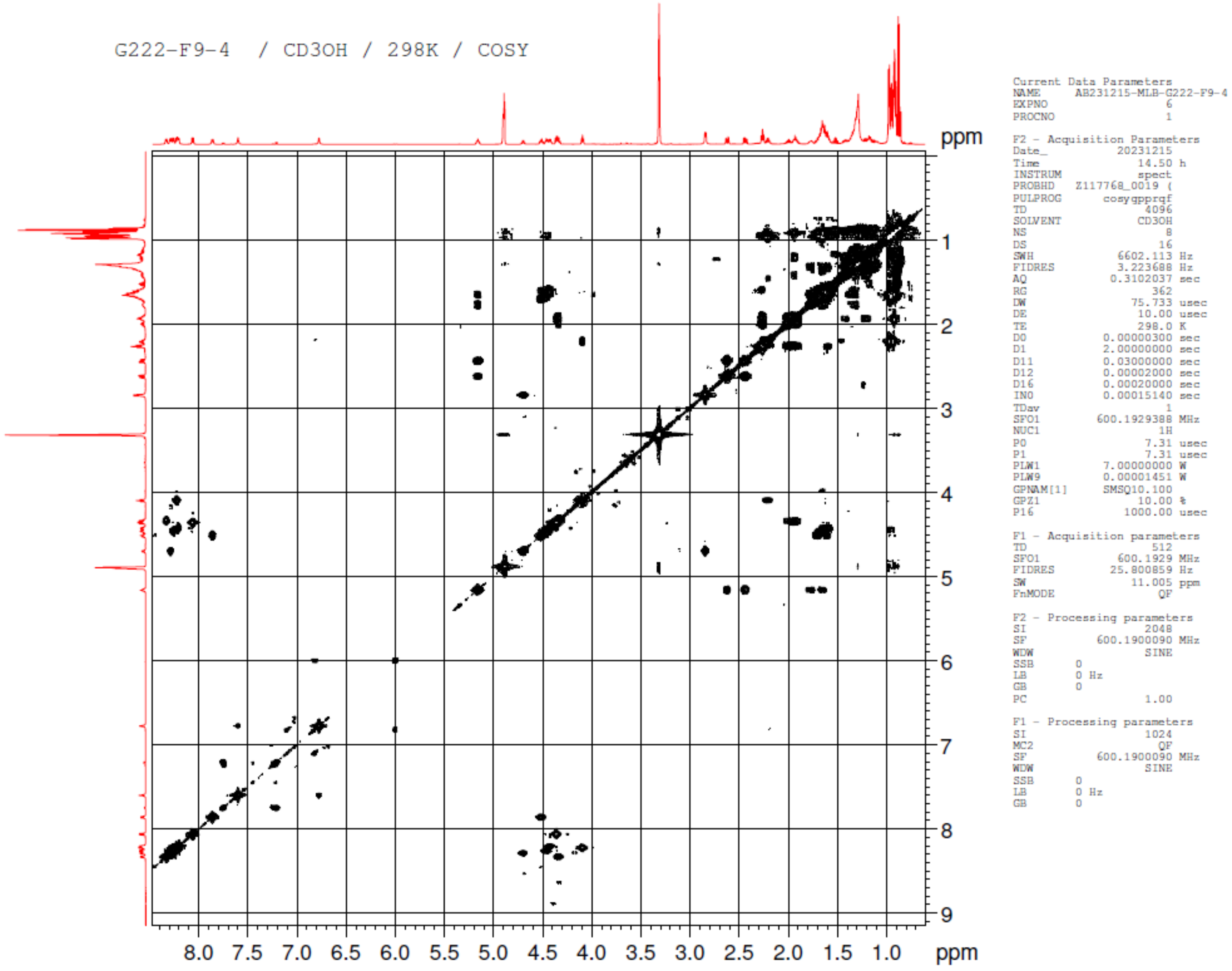

Figure S22: COSY-NMR spectrum of Lichenysin (**3-4**) (*m*/*z* 1007.6734 and 1007.6733 [M + H]+) (600 MHz, CD_3OH).

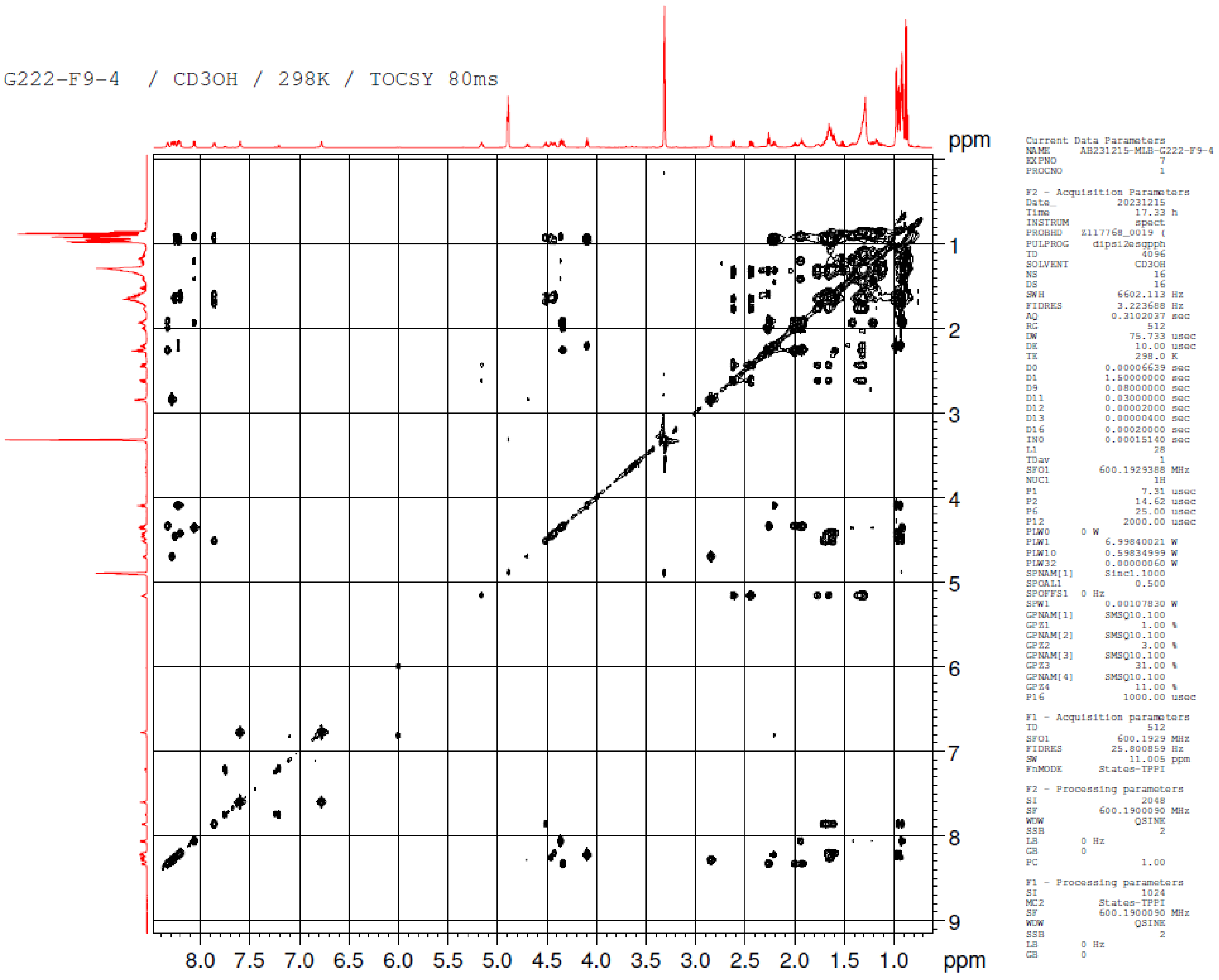

Figure S23: TOCSY-NMR spectrum of Lichenysin (**3-4**) (*m/z* 1007.6734 and 1007.6733 [M + H]$^+$) (600 MHz, CD_3OH).

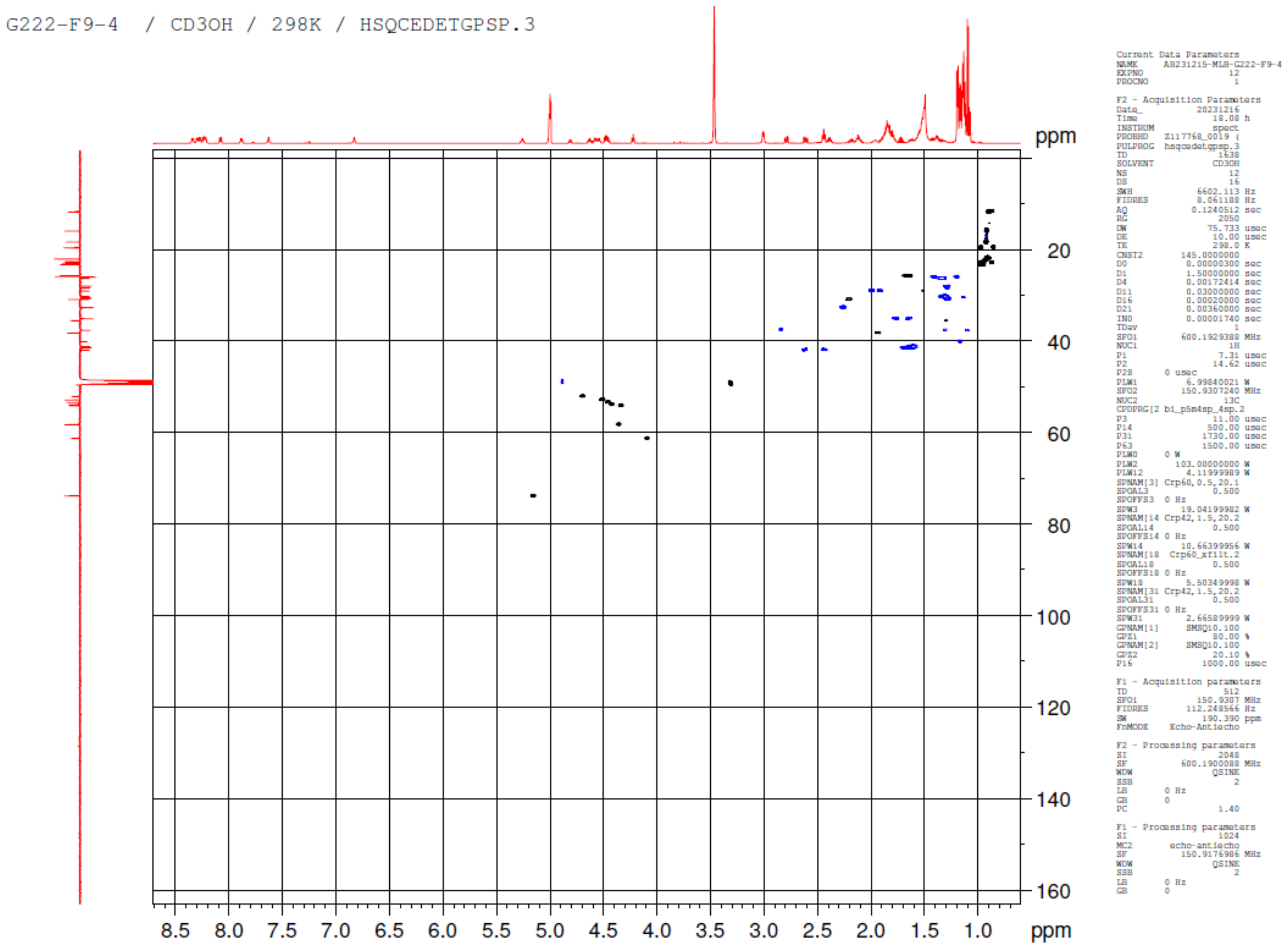

Figure S24: HSQC-NMR spectrum of Lichenysin (**3-4**) (*m*/*z* 1007.6734 and 1007.6733 [M + H]+) (600 MHz, CD_3OH)

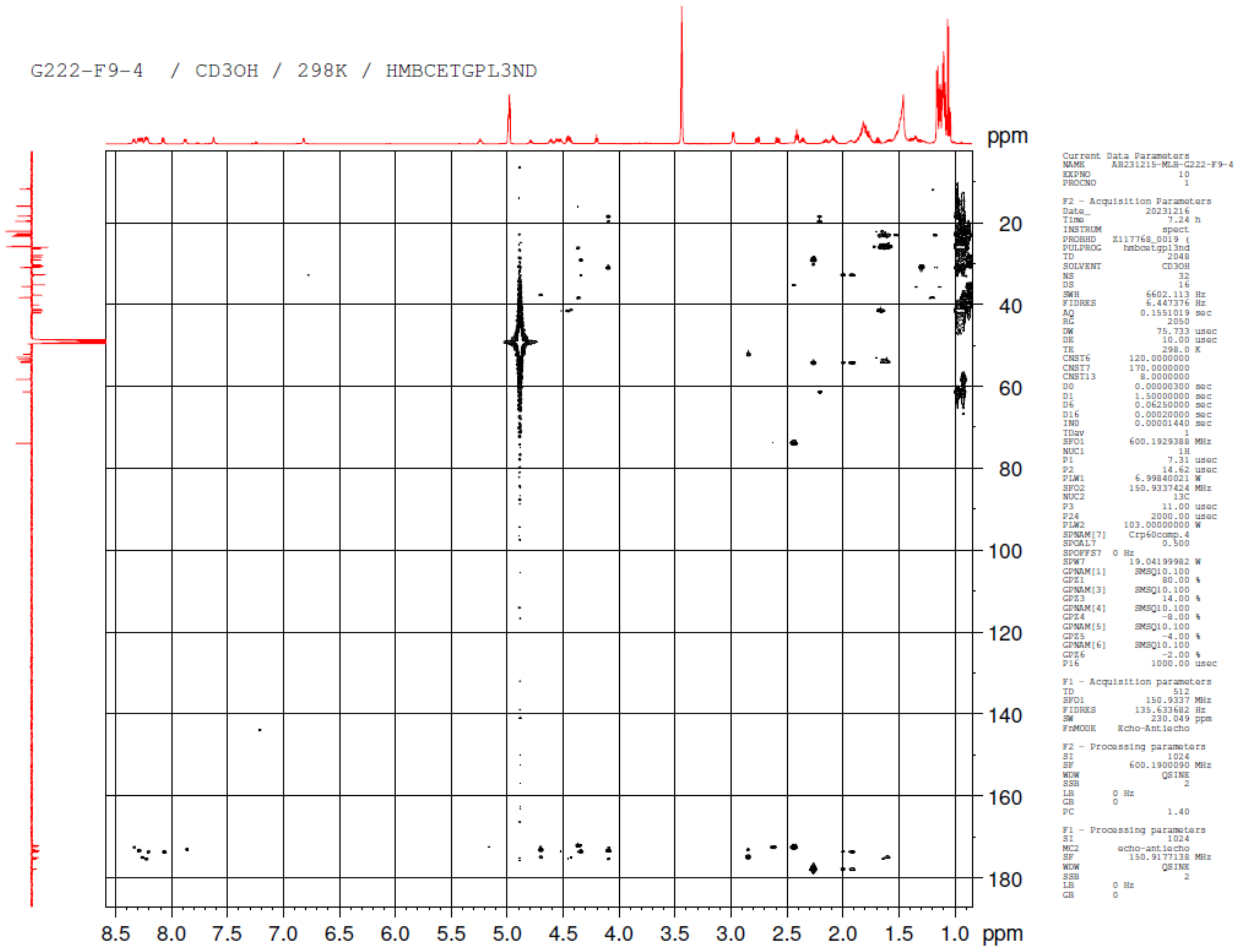

Figure S25: HMBC-NMR spectrum of Lichenysin (**3**-**4**) (*m/z* 1007.6734 and 1007.6733 [M + H]$^+$) (600 MHz, CD_3OH).

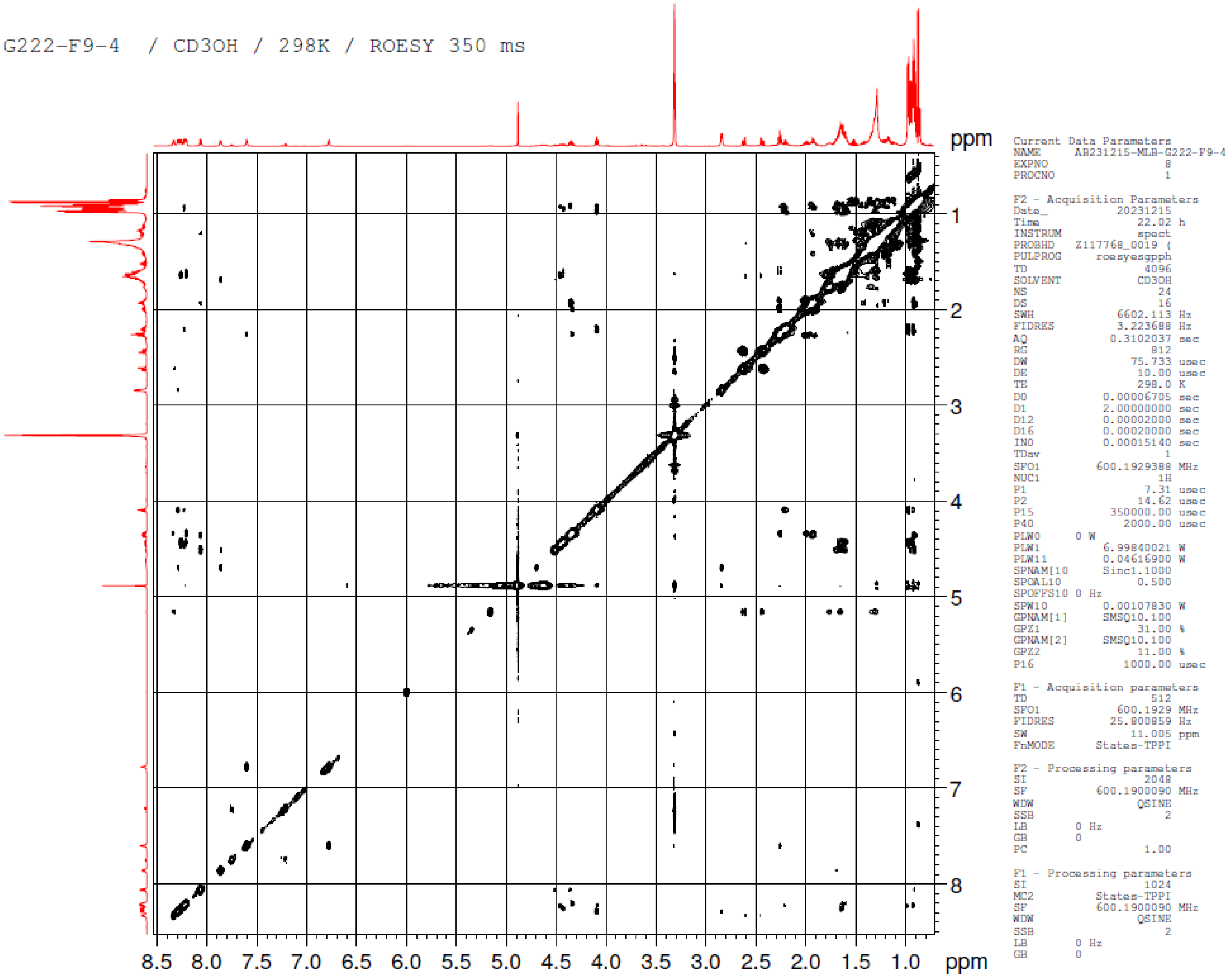

Figure S26: ROESY-NMR spectrum of Lichenysin (**3-4**) (*m/z* 1007.6734 and 1007.6733 [M + H]$^+$) (600 MHz, CD_3OH).

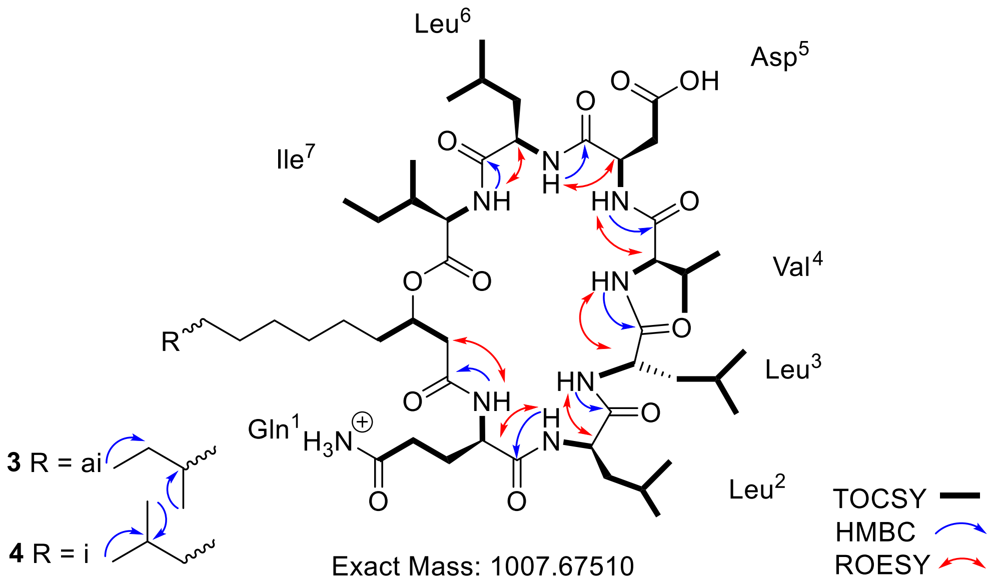

Figure S27: Structure of Lichenysins (**3-4**) (*m/z* 1007.6734 and 1007.6733 [M + H]$^+$; respectively).

Table S16. ^{1}H, ^{13}C, HMBC, and ROESY NMR data of Lichenysin (**3**-**4**) (*m/z* 1007.6734 and 1007.6733 [M + H]$^{+}$) (600 MHz, CD_3OH), isoform *ante-iso* and *iso* (0.8:0.2).

Unit	Pos.	δ_C, Mult.		δ_H (*J* in Hz)	HMBC	ROESY [a]
L-Gln¹	NH	-		8.33, d (7.46)	Gln¹-α, FA-CO,	FA-2-a, FA-2-b, FA-3
	C=O	173.62, C		-	-	-
	α-C	54.15, CH		4.33, ddd (7,46, 9.93, 14.13)	Gln¹-CO, Gln¹-β, Gln¹- γ	Leu²-NH
	β-C	29.05, CH_2	a.<br>b.	1.99, m<br>1.91, m	Gln¹-CO, Gln¹-α, Gln¹-γ, Gln¹-δ-C=O	Leu²-NH
	γ-C	32.75, CH_2		2.26, t (7.46)	Gln¹-α, Gln¹-β, Gln¹-δ-C=O	Leu²-NH
	δ-C=O	177.87, CO²		-	-	-
	ε-NH_2	-	a.<br>b.	7.61, brs<br>6.77, brs	Gln¹-δ-C=O, Gln¹- γ	-
L-Leu²	NH	-		8.20, dd (7.57)	Gln¹-CO	Gln¹-α, Gln¹-β, Gln¹-γ
	C=O	175.09, C		-	-	-
	α-C	53.90, CH		4.42, ddd (5.93, 7.57, 14.06)	Leu²-CO, Leu²-β, Leu²-γ	Leu³-NH
	β-C	41.17, CH_2	a.<br>b	1.64, m<br>1.61, m	Leu²-CO, Leu²-α, Leu²-γ	Leu³-NH
	γ-C	25.87, CH		1.64, m	Leu²-α, Leu²-β, Leu²-δ, Leu²-δ′	Leu³-NH
	δ-C	22.98, CH_3		0.97, d (4.13)	Leu²-β, Leu²-γ, Leu²-δ′	-
	δ′-C	23.28, CH_3		0.95, d (3.79)	Leu²-β, Leu²-γ, Leu²-δ	-
D-Leu³	NH	-		8.26, d (7.18)	Leu²-CO, Leu³-α	Leu²-α, Leu²-β, Leu²-γ
	C=O	175.52, C		-	-	-
	α-C	53.44, CH		4.45, ddd (4.31, 7.18, 12.09)	Leu³-CO, Leu³-β, Leu³-γ	Val⁴-NH
	β-C	41.44, CH_2	a.<br>b	1.61, m<br>1.64, m	Leu³-CO, Leu³-α, Leu³-γ	Val⁴-NH
	γ-C	25.70, CH		1.64, m	Leu³-α, Leu³-β	Val⁴-NH
	δ-C	22.07, CH_3		0.91, d (4.31)	Leu³-β, Leu³-γ, Leu³-δ′	-
	δ′-C	23.26, CH_3		0.97, d (4.31)	Leu³-β, Leu³-γ, Leu³-δ	-
L-Val⁴	NH	-		8.22, d (7.32)	Val⁴-α, Val⁴-β, Leu³-CO	Leu³-α, Leu³-β, Leu³-γ
	C=O	173.35 C		-	-	-
	α-C	61.26, CH		4.09, dd (5.54, 7.32)	Val⁴-CO, Val⁴-β, Val⁴-γ, Val⁴-γ′	Asp⁵-NH
	β-C	30.89, CH		2.20, ddd (5.54, 7.32, 13.92)	Val⁴-α, Val⁴-γ, Val⁴-γ′	Asp⁵-NH
	γ-C	19.62, CH_3		0.97, d (5.54)	Val⁴-β, Val⁴-γ′	Asp⁵-NH
	γ′-C	18.31, CH_3		0.92, d (5.54)	Val⁴-β, Val⁴-γ	Asp⁵-NH
L-Asp⁵	NH	-		8.28, d (7.46)	Val⁴-CO, Asp⁵-α	Val⁴-α, Val⁴-β, Val⁴-γ, Val⁴-γ′, Leu⁶-NH
	C=O	173.11, C		-	-	-
	α-C	52.11, CH		4.70, dd (7.46, 13.76)	Asp⁵-CO, Asp⁵-β, Asp⁵-γ-C=O	Leu⁶-NH
	β-C	37.64, CH_2		2.84, d (7.46)	Asp⁵-CO, Asp⁵-α, Asp⁵-γ-C=O	Leu⁶-NH
	γ-C=O	175.07, CO²		-	-	-
D-Leu⁶	NH	-		7.85, d (8.32)	Asp⁵-CO	Asp⁵-α, Asp⁵-NH, Ile⁶-NH
	C=O	173.60, C		-	-	-
	α-C	52.91, CH		4.51, ddd (5.21, 8.32, 14.15)	Leu⁶-CO, Leu⁶-β,	Ile⁷-NH
	β-C	41.49, CH_2	a.<br>b	1.70, m<br>1.60, m	Leu⁶-CO, Leu⁶-α, Leu⁶-γ	Ile⁷-NH
	γ-C	25.86, CH		1.64, m	Leu⁶-α, Leu⁶-β	Ile⁷-NH
	δ-C	22.07, CH_3		0.91, d (1.68)	Leu⁶-β, Leu³-δ	-
	δ′-C	22.65, CH_3		0.94, d (1.89)	Leu⁶-β, Leu⁶-δ′	-

Unit	Pos.	δC, Mult.		δH (*J* in Hz)	HMBC	ROESY [a]
L-Ile[7]	NH	-		8.07, d (8.47)	Leu[6]-CO	Leu[6]-NH, Leu[6]-α, Leu[6]-β, Leu[6]-γ
	C=O	172.13, C		-	-	-
	α-C	58.30, CH		4.36, dd (5.29, 8.47)	Ile[7]-CO, Ile[7]-β, Ile[7]-γ, Ile[7]-δ	-
	β-C	38.24, CH		1.93, q (8.47)	Ile[7]-β, Ile[7]-γ, Ile[7]-δ, Ile[7]-δ′	-
	γ-C	25.99, CH_2	a.<br>b	1.41, m<br>1.20, m	Ile[7]-β, Ile[7]-δ, Ile[7]-δ′	-
	δ-C	15.93, CH_3		0.92, d (3.66)	Ile[7]-α, Ile[7]-β, Ile[7]-γ	-
	δ′-C	11.77, CH_3		0.89, t (3.12)	Ile[7]-β, Ile[7]-γ	-
Fatty acid	C=O	172.55, C		-	-	-
	2	41.96, CH_2	a.<br>b	2.62, dd (5.02, 12.97)<br>2.43, dd (7.46, 12.97)	FA-3, FA-4, FA-CO	Gln[1]-NH
	3	73.83, CH		5.15 ddd (5.02, 7.46, 12.97)	FA-4, FA-CO	Gln[1]-NH
	4	35.14, CH_2	a.<br>b	1.77, m<br>1.65, m	FA-2-a, FA-2-b, FA-3<br>FA-3, FA-5-9	-<br>-
ai	5	26.32, CH_2*		1.35[b], m	FA-5-9, FA-10, FA-11	-
ai	6	27.98, CH_2*		1.29[b], m	FA-5-9, FA-10, FA-11	-
ai	7	30.24, CH_2*		1.29[b], m	FA-5-9, FA-10, FA-11	-
ai	8	30.53, CH_2*		1.29[b], m	FA-5-9, FA-10, FA-11	-
ai	9	37.67, CH_2	a.<br>b	1.09, m<br>1.29, m	FA-5-9, FA-10, FA-11	-
ai	10	35.54, CH		1.29[b], m	FA-11, FA-5-9	-
ai	11	30.53, CH_2	a.<br>b	1.29[b], m<br>1.13[b], m	FA-10, FA-12, FA-13	-
ai	12	19.50, CH_3		0.85, t (4.54)	FA-10	-
ai	13	11.63, CH_3		0.86, d (5.32)	FA-9, FA-10, FA-11	-
i	5	26.33, CH_2*		1.35[b], m	FA-5-9, FA-10, FA-11	-
i	6	28.36, CH_2*		1.29[b], m	FA-5-9, FA-10, FA-11	-
i	7	30.28, CH_2*		1.29[b], m	FA-5-9, FA-10, FA-11	-
i	8	30.78, CH_2*		1.29[b], m	FA-5-9, FA-10, FA-11	-
i	9	26.32, CH_2		1.29[b], m	FA-10, FA-12, FA-13	-
i	10	40.13, CH_2		1.17[b], m	FA-11, FA-12, FA-13	-
i	11	29.02, CH		1.51, ept (6.65)	FA-10, FA-12, FA-13	-
i	12	22.91, CH_3		0.87, d (6.65)	FA-9, FA-11, FA-13	-
i	13	22.91, CH_3		0.87, d (6.65)	FA-9, FA-11, FA-12	-

[a] Sequential ROEs [b] Overlapped signals prevent determination of constant couplings * May be interchanged.

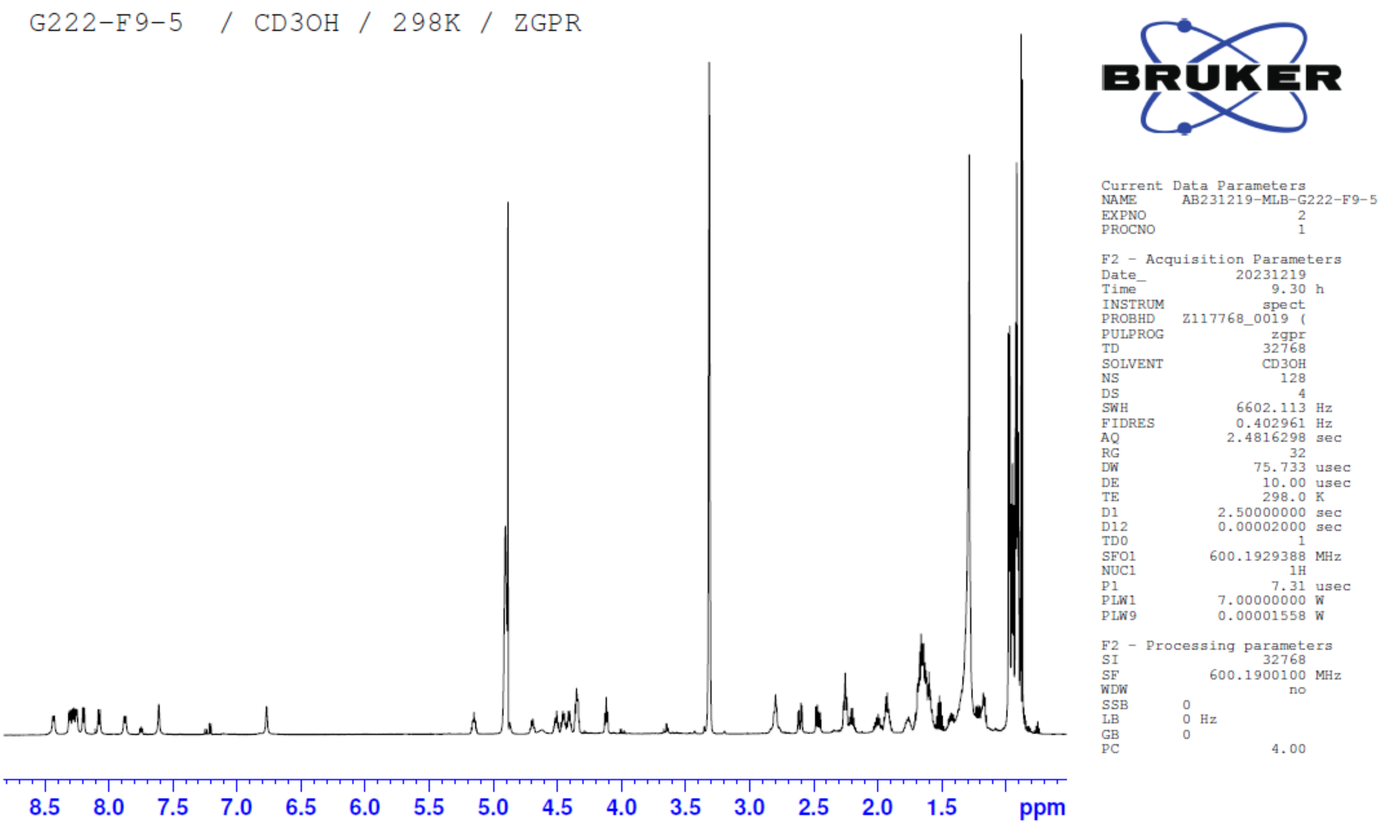

Figure S28: ¹H-NMR spectrum of Lichenysin (**5a** and **5b**) (*m/z* 1021.6896 [M + H]⁺) (600 MHz, CD_3OH).

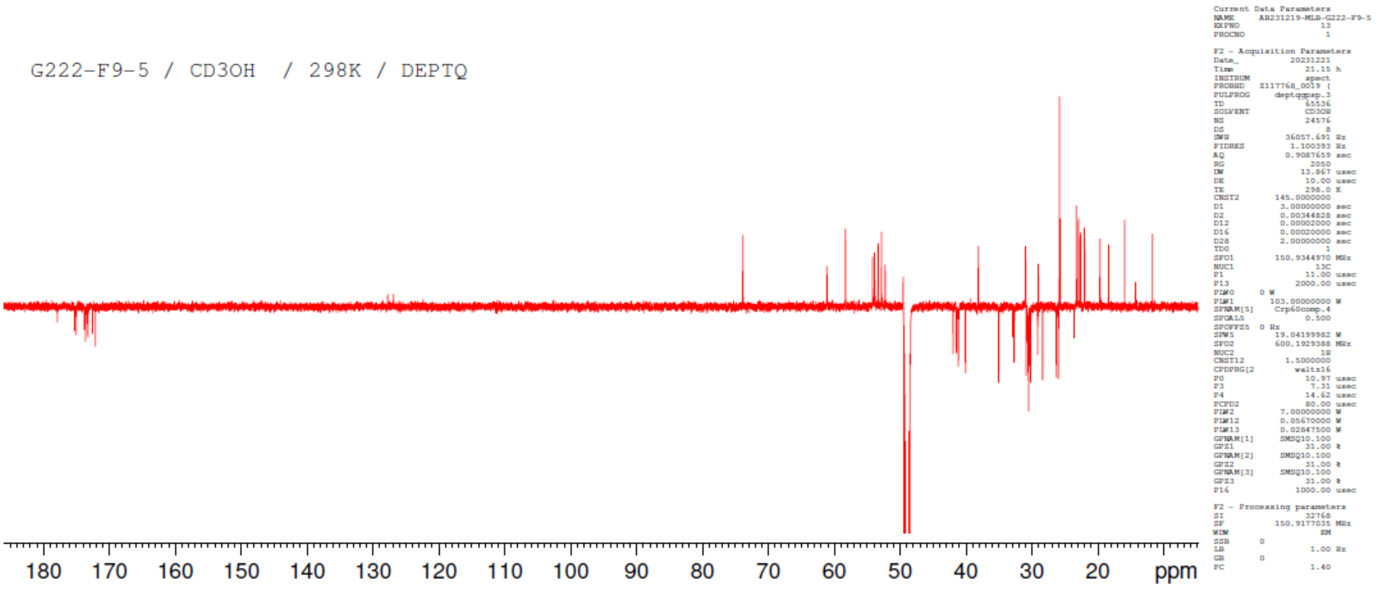

Figure S29: DEPTQ spectrum of Lichenysin (**5a** and **5b**) (*m/z* 1021.6896 [M + H]⁺) (600 MHz, CD_3OH).

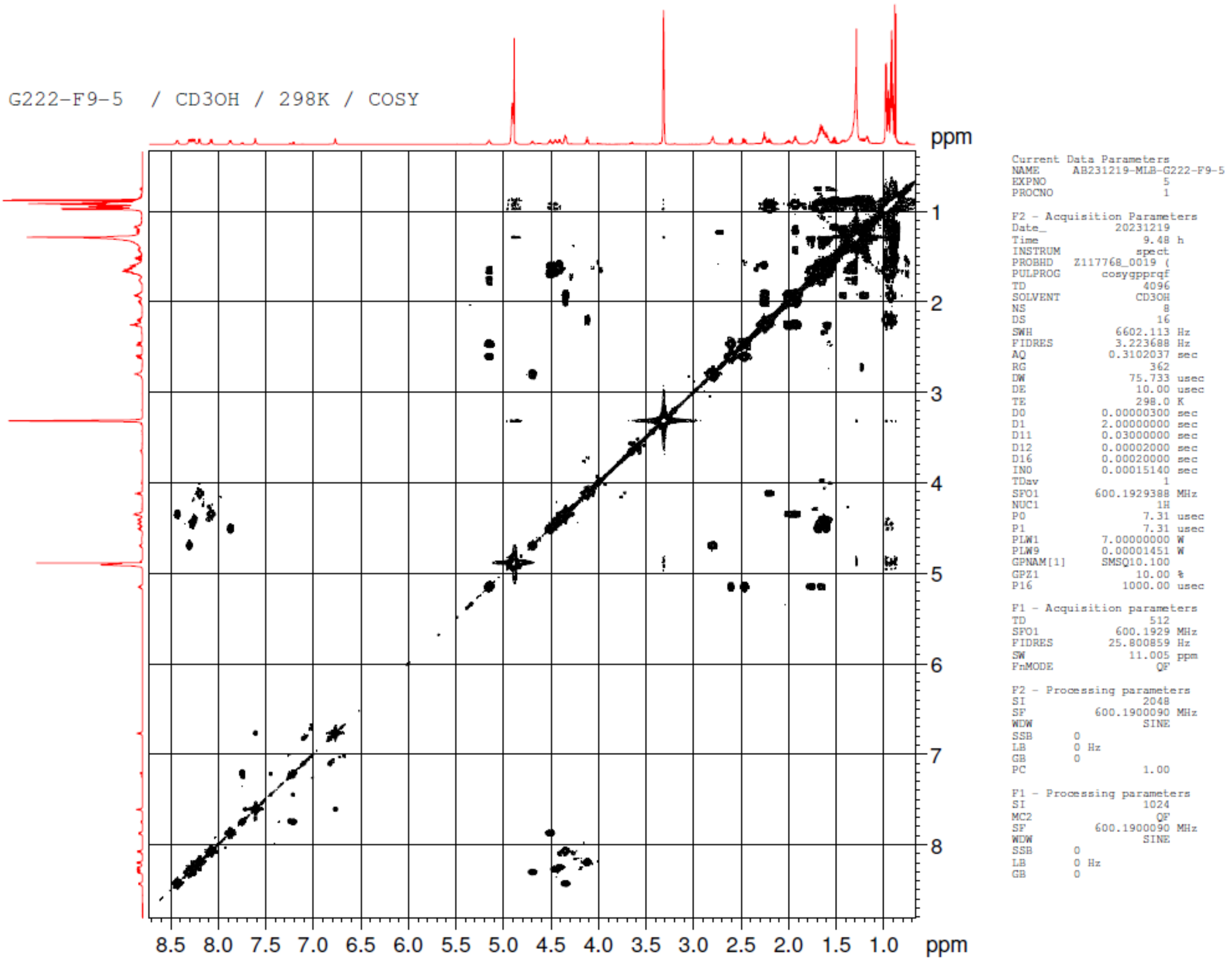

Figure S30: COSY-NMR spectrum of Lichenysin (**5a** and **5b**) (*m/z* 1021.6896 [M + H]+) (600 MHz, CD_3OH).

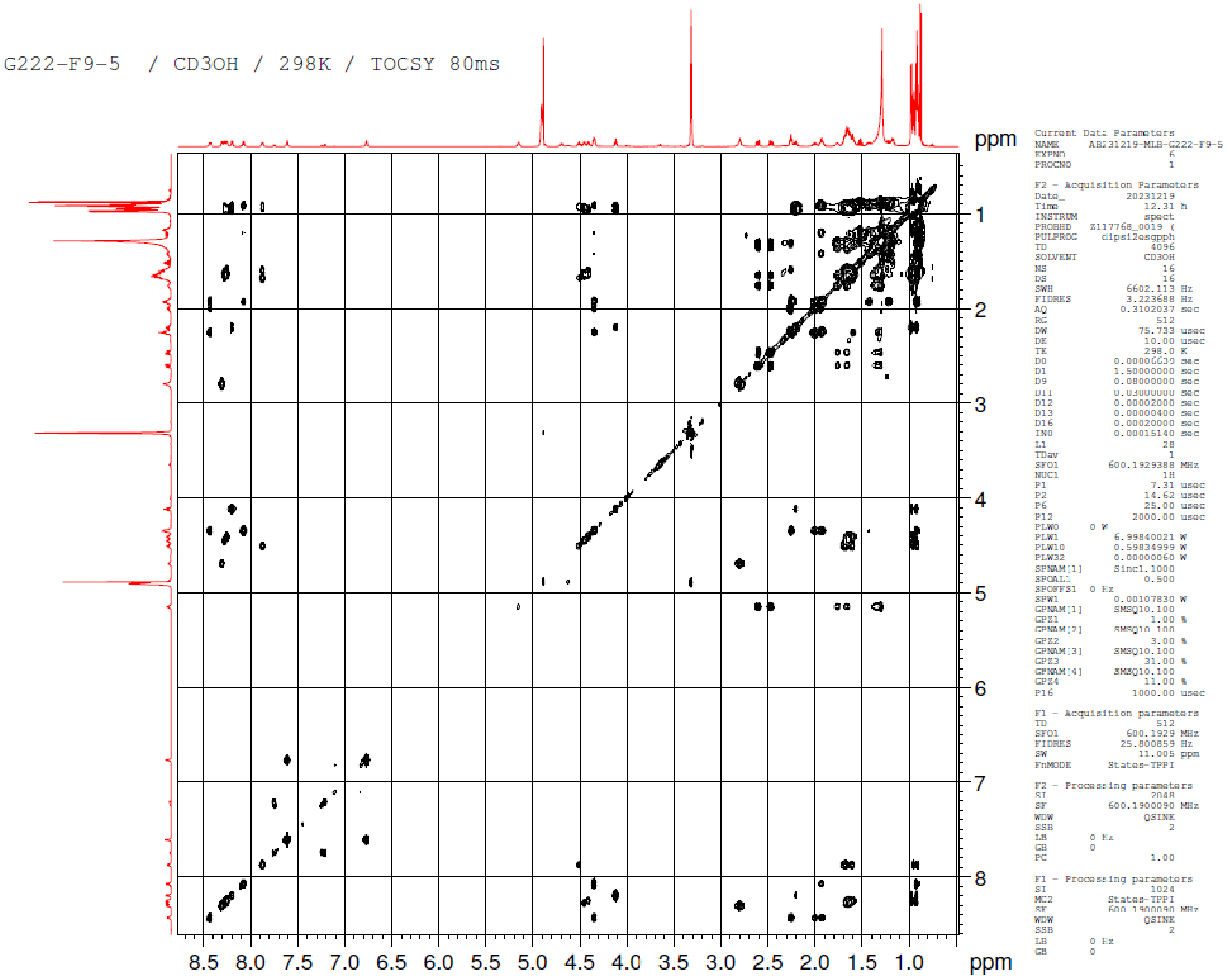

Figure S31: TOCSY-NMR spectrum of Lichenysin (**5a** and **5b**) (*m*/*z* 1021.6896 [M + H]$^+$) (600 MHz, CD_3OH).

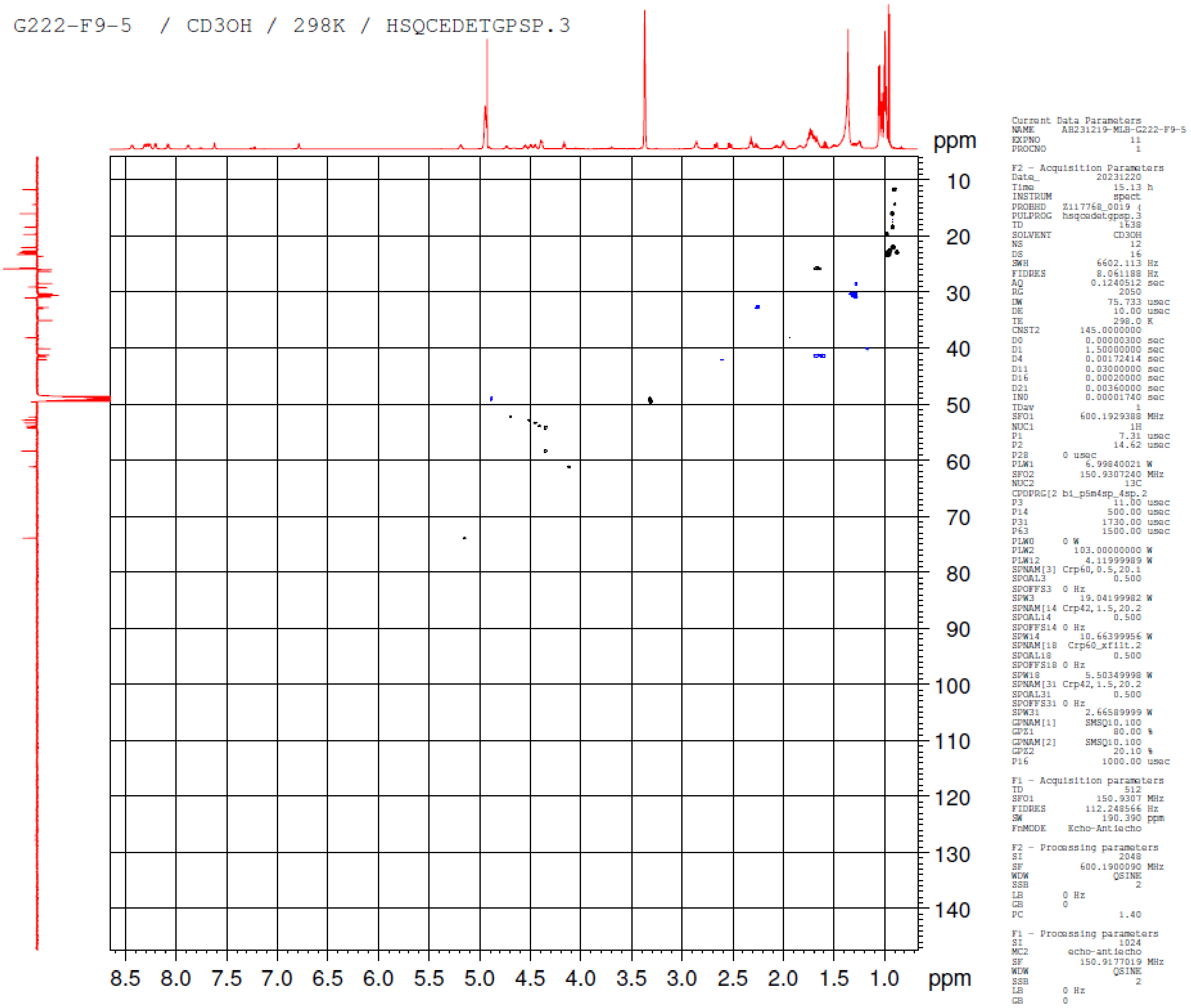

Figure S32: HSQC-NMR spectrum of Lichenysin (**5a** and **5b**) (*m*/*z* 1021.6896 $[M + H]^+$) (600 MHz, CD_3OH).

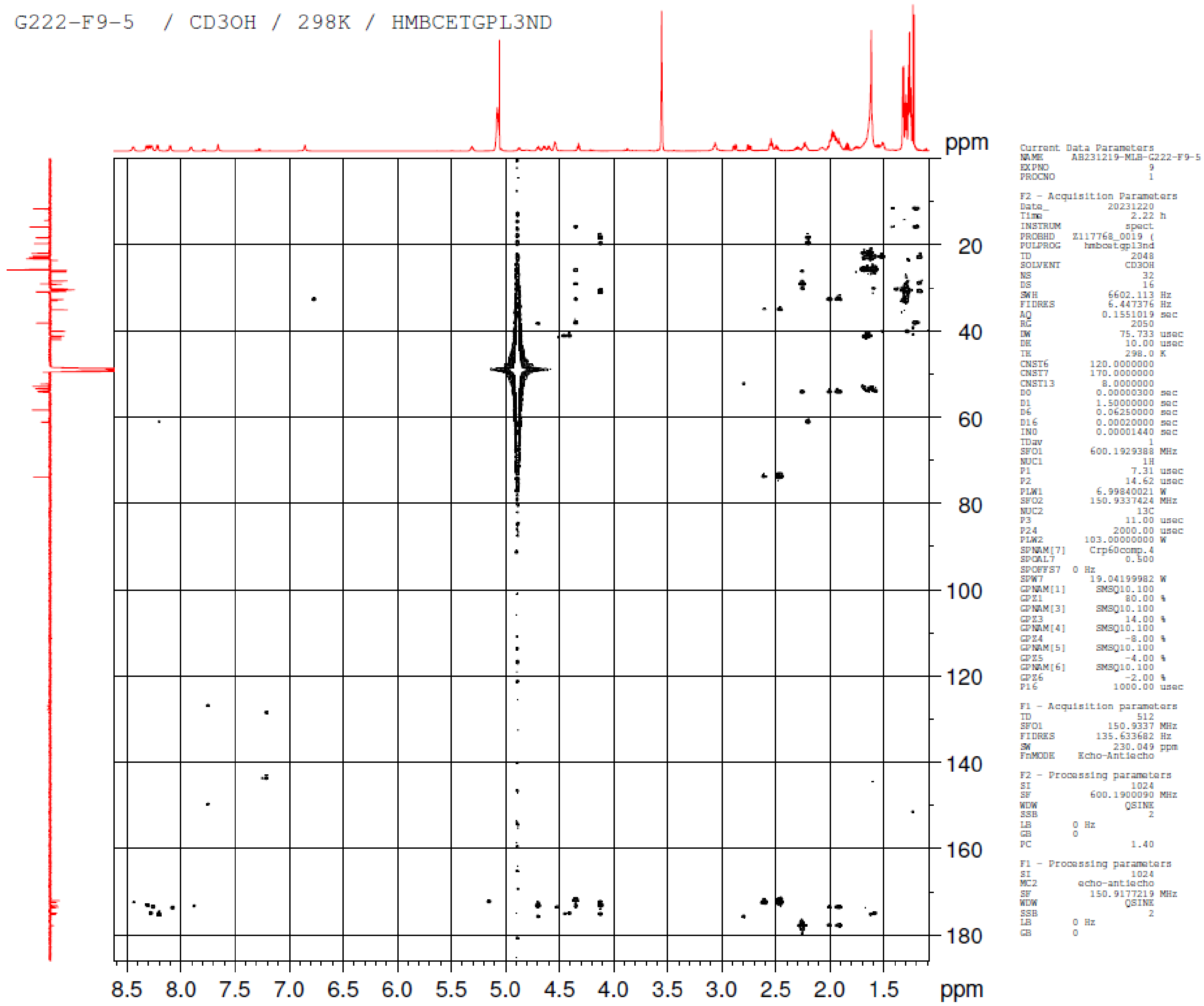

Figure S33: HMBC-NMR spectrum of Lichenysin (**5a** and **5b**) (*m*/*z* 1021.6896 [M + H]+) (600 MHz, CD_3OH).

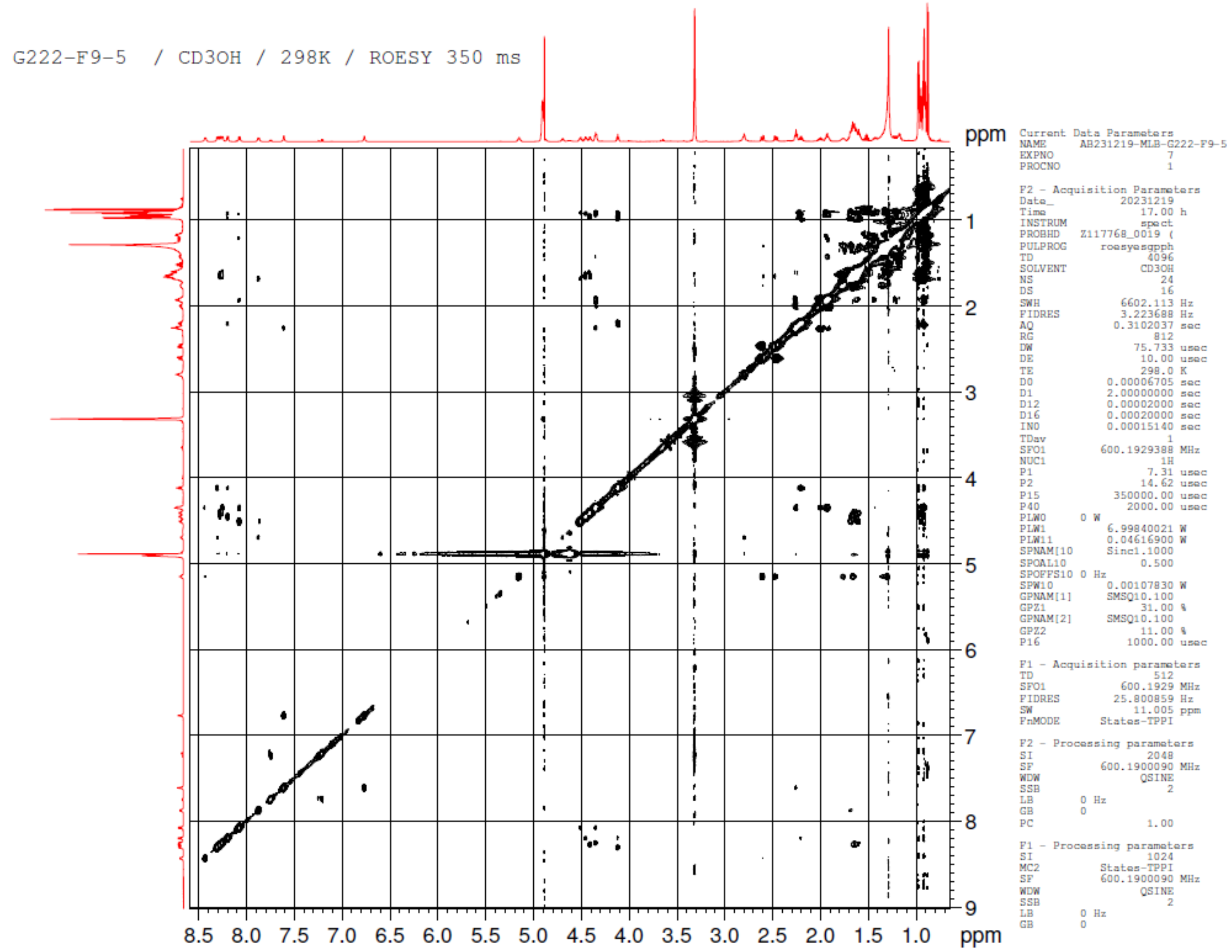

Figure S34: ROESY-NMR spectrum of Lichenysin (**5a** and **5b**) (*m*/*z* 1021.6896 [M + H]$^+$) (600 MHz, CD_3OH).

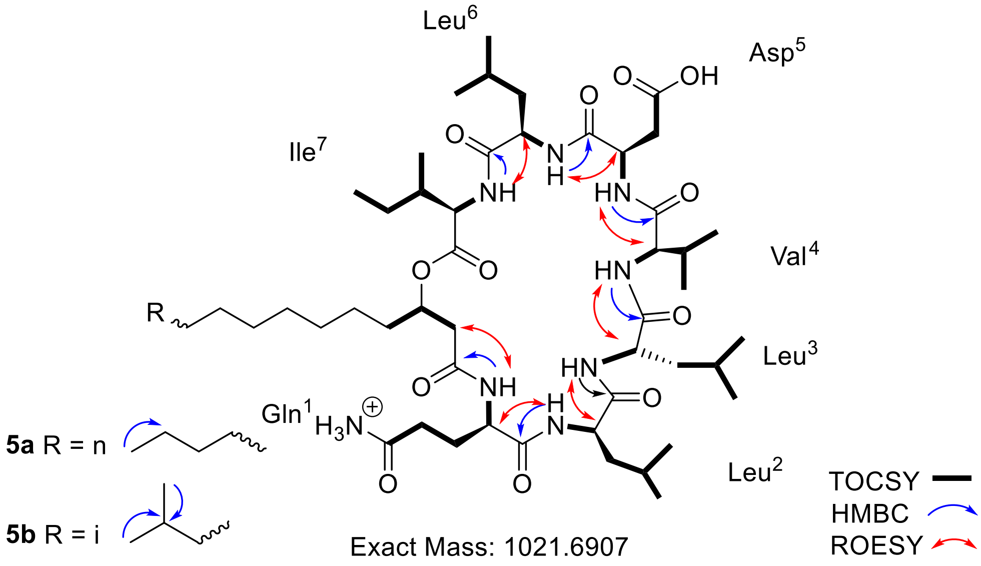

Figure S35: Structure of Lichenysins (**5a** and **5b**) (*m*/*z* 1021.6896 [M + H]$^+$).

Table S17. ^{1}H, ^{13}C, HMBC, and ROESY NMR data of Lichenysin (**5a** and **5b**) (m/z 1021.6896 [M + H]$^+$) (600 MHz, CD_3OH) isoform: *iso* and *n* (0.5:0.5).

Unit	Pos.	δC, Mult.		δH (*J* in Hz)	HMBC	ROESY[a]
L-Gln[1]	NH	-		8.43, d (7.72)	FA-CO	FA-2-a, FA-2-b, FA-3
	C=O	173.63, C		-	-	-
	α-C	54.19, CH		4.34, ddd (4.72, 7.72, 10.13)	Gln[1]-CO, FA-CO	Leu[2]-NH
	β-C	29.13, CH_2	a.<br>b.	1.98, m<br>1.91, m	Gln[1]-CO, Gln[1]-α, Gln[1]- γ, Gln[1]-δ-C=O	Leu[2]-NH
	γ-C	32.72, CH_2		2.25, t (7.72)	Gln[1]-α, Gln[1]-β, Gln[1]-δ-C=O	Leu[2]-NH
	δ-C=O	177.89, CO^2		-	-	-
	ε-NH_2	-	a.<br>b.	7.61, brs<br>6.80 brs	Gln[1]-δ-C=O, Gln[1]-γ	-
L-Leu[2]	NH	-		8.24, d (5.82)	Leu[2]-α, Leu[2]-β,Gln[1]-CO	Gln[1]-α, Gln[1]-β, Gln[1]-γ
	C=O	175.01, C		-	-	-
	α-C	53.90, CH		4.41, ddd (2.26, 5.82, 7.62)	Leu[2]-CO, Leu[2]-β, Leu[2]-γ, Gln[1]-CO	Leu[3]-NH
	β-C	41.18, CH_2	a.<br>b	1.63 m<br>1.59 m	Leu[2]-CO, Leu[2]-α, Leu[2]-γ	Leu[3]-NH
	γ-C	25.87, CH		1.64, m	Leu[2]-α, Leu[2]-β, Leu[2]-δ, Leu[2]-δ′	Leu[3]-NH
	δ-C	23.27, CH_3		0.95, d (2.26)	Leu[2]-γ, Leu[2]-δ′	-
	δ′-C	23.27, CH_3		0.97, d (2.26)	Leu[2]-γ, Leu[2]-δ	-
D-Leu[3]	NH	-		8.27, d (7.31)	Leu[2]-CO, Leu[3]-α	Leu[2]-α, Leu[2]-β, Leu[2]-γ
	C=O	175.23, C		-	-	-
	α-C	53.36, CH		4.45, ddd (5.08, 7.31, 11.85)	Leu[3]-CO, Leu[3]-β, Leu[3]-γ	Val[4]-NH
	β-C	41.24, CH_2	a.<br>b.	1.63, m<br>1.61, m	Leu[3]-CO, Leu[3]-α, Leu[3]-γ	Val[4]-NH
	γ-C	25.72, CH		1.64, m	Leu[3]-α, Leu[3]-β	Val[4]-NH
	δ-C	23.00, CH_3		0.97, d (1.69)	Leu[3]-γ, Leu[3]-δ′	-
	δ′-C	22.05, CH_3		0.91, d (1.69)	Leu[3]-γ, Leu[3]-δ	-
L-Val[4]	NH	-		8.19, d (7.72)	Val[4]-α, Leu[3]-CO	Leu[3]-α, Leu[3]-β, Leu[3]-γ
	C=O	173.28, C		-	-	-
	α-C	61.17, CH		4.14, dd (7.72, 13.83)	Val[4]-CO, Val[4]-β, Val[4]-γ, Val[4]-γ′	Asp[5]-NH
	β-C	31.03, CH		2.20, ddd (3.85, 6.93, 11.80)	Val[4]-α, Val[4]-γ, Val[4]-γ′	Asp[5]-NH
	γ-C	19.69, CH_3		0.97, d (1.89)	Val[4]-α, Val[4]-β, Val[4]-γ′	Asp[5]-NH
	γ′-C	18.41, CH_3		0.92, d (1.89)	Val[4]-α, Val[4]-β, Val[4]-γ	Asp[5]-NH
L-Asp[5]	NH	-		8.31, d (7.53)	Val[4]-CO	Val[4]-α, Val[4]-β, Val[4]-γ, Val[4]-γ′, Leu[6]-NH
	C=O	173.41, C		-	-	-
	α-C	52.30, CH		4.69, dd (7.53, 13.75)	Asp[5]-CO, Asp[5]-β, Asp[5]-γ-C=O	Leu[6]-NH

	β-C	38.31, CH$_2$		2.81, dd (7.53, 13.75)	Asp^5-CO, Asp^5-α, Asp^5-γ-C=O	Leu^6-NH
	γ-C=O	175.82, CO^2		-	-	-
D-Leu^6	NH	-		7.88, d (8.69)	Asp^5-CO	Asp^5-α, Asp^5-NH, Ile^6-NH
	C=O	173.80, C		-	-	-
	α-C	52.89, CH		4.51 ddd (5.12, 8.69, 13.32)	Leu^6-CO, Leu^6-β,	Ile^7-NH
	β-C	41.50, CH$_2$	a.<br>b	1.68, m<br>1.61, m	Leu^6-CO, Leu^6-α, Leu^6-γ	Ile^7-NH
	γ-C	25.86, CH		1.64, m	Leu^6-α, Leu^6-β	Ile^7-NH
	δ-C	21.98, CH$_3$		0.91, d (1.68)	Leu^6-β, Leu^3-δ	-
	δ′-C	22.65, CH$_3$		0.94, d (1.89)	Leu^6-β, Leu^6-δ′	-
L-Ile^7	NH	-		8.07, d (8.53)	Leu^6-CO	Leu^6-NH, Leu^6-α, Leu^6-β, Leu^6-γ
	C=O	172.13, C		-	-	-
	α-C	58.35, CH		4.35, ddd (4.31, 8.53, 11.47)	Ile^7-CO, Ile^7-β, Ile^7-γ, Ile^7-δ	-
	β-C	38.13, CH		1.92, d (8.53)	Ile^7-CO	-
	γ-C	25.99, CH$_2$	a.<br>b	1.43, m<br>1.20, m	Ile^7-β, Ile^7-δ, Ile^7-δ′	-
	δ-C	15.96, CH$_3$		0.92, d (4.31)	Ile^7-α, Ile^7-β	-
	δ′-C	11.72, CH$_3$		0.90, t (2.33)	Ile^7-β	-
Fatty acid	C=O	172.55, C		-	-	-
	2	41.99, CH$_2$	a.<br>b	2.61, dd<br>2.46, dd	FA-3, FA-4, FA-CO	Gln^1-NH
	3	73.83, CH		5.15 ddd (5.24, 6.89, 12.81)	FA-4, FA-CO	Gln^1-NH
	4	35.11, CH$_2$	a.<br>b	1.75, m<br>1.65, m	FA-2-a, FA-2-b, FA-3<br>FA-5-9, FA-10, FA-11	-<br>-
	5	30.22, CH$_2$		1.35^b, m	FA-5-9, FA-10, FA-11	-
	6	30.34, CH$_2$		1.29^b, m	FA-5-9, FA-10, FA-11	-
	7	30.52, CH$_2$		1.29^b, m	FA-5-9, FA-10, FA-11	-
	8	30.54, CH$_2$		1.29^b, m	FA-5-9, FA-10, FA-11	-
	9	30.60, CH$_2$		1.29^b, m	FA-5-9, FA-10, FA-11	-
i	10	26.35, CH$_2$		1.29^b, m	FA-11, FA-5-9	-
i	11	40.02, CH$_2$		1.16, m	FA-12	-
i	12	29.06, CH		1.51, m	FA-11	-
i	13	22.92, CH$_3$		0.87, d (6.60)	FA-11, FA-12, FA-14	-
i	14	22.92, CH$_3$		0.87, d (6.60)	FA-11, FA-12, FA-13	-
n	10	30.65, CH$_2$		1.29^b, m	FA-11, FA-5-9	-
n	11	30.90, CH$_2$		1.29^b, m	FA-12	-
n	12	29.06, CH$_2$		1.29^b, m	FA-11	-
n	13	23.59, CH$_2$		1.29^b, m	FA-11, FA-12, FA-14	-
n	14	14.32, CH$_3$		0.90, t (6.60)	FA-11, FA-12, FA-13	-

^{a} Sequential ROEs. ^{b} Overlapped signals prevent determination of constant couplings.

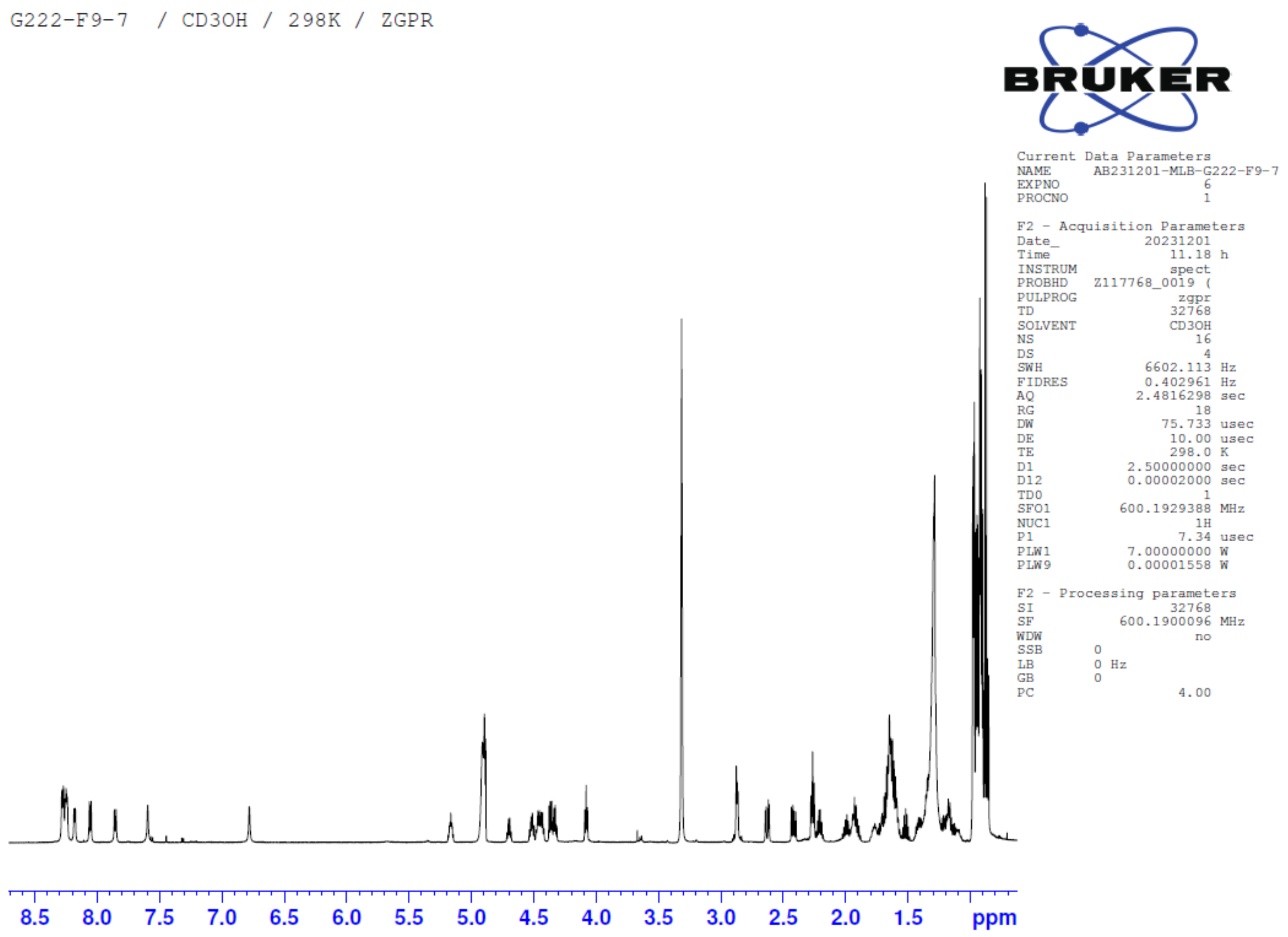

Figure S36: ^{1}H-NMR spectrum of Lichenysin (**6-7**) (*m/z* 1035.7063 [M + H]+) (600 MHz, CD_3OH).

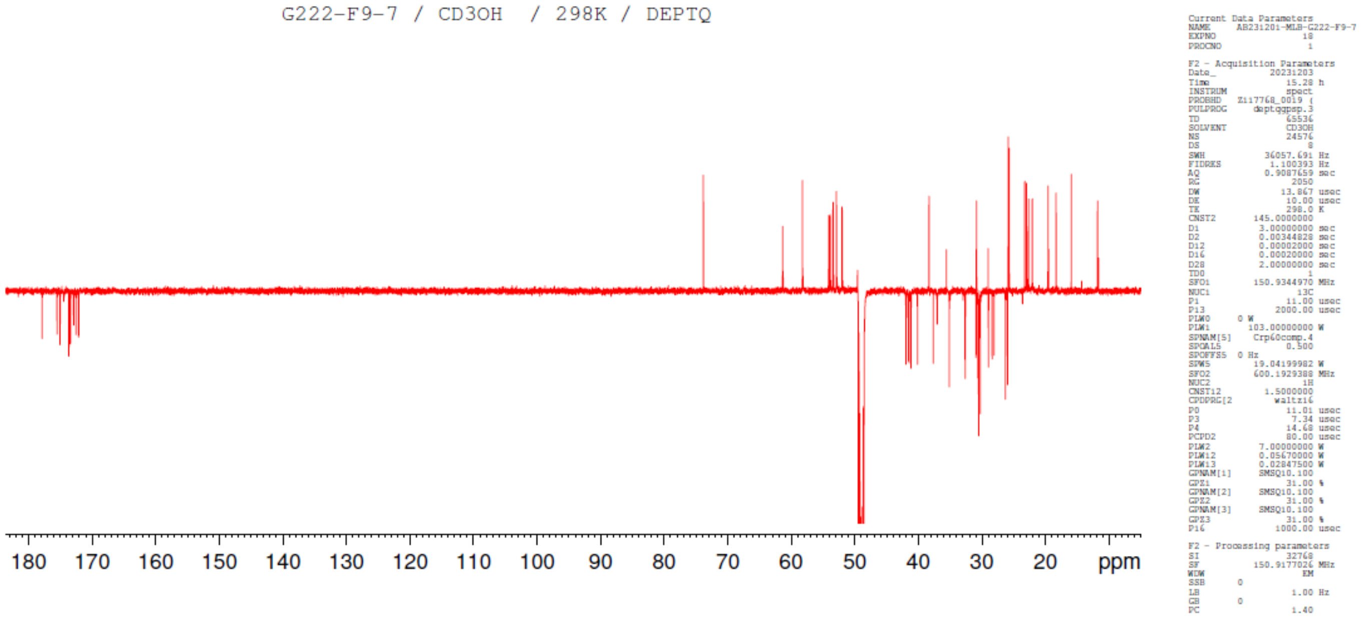

Figure S37: DEPTQ spectrum of Lichenysin (**6-7**) (*m/z* 1035.7063 [M + H]+) (600 MHz, CD_3OH).

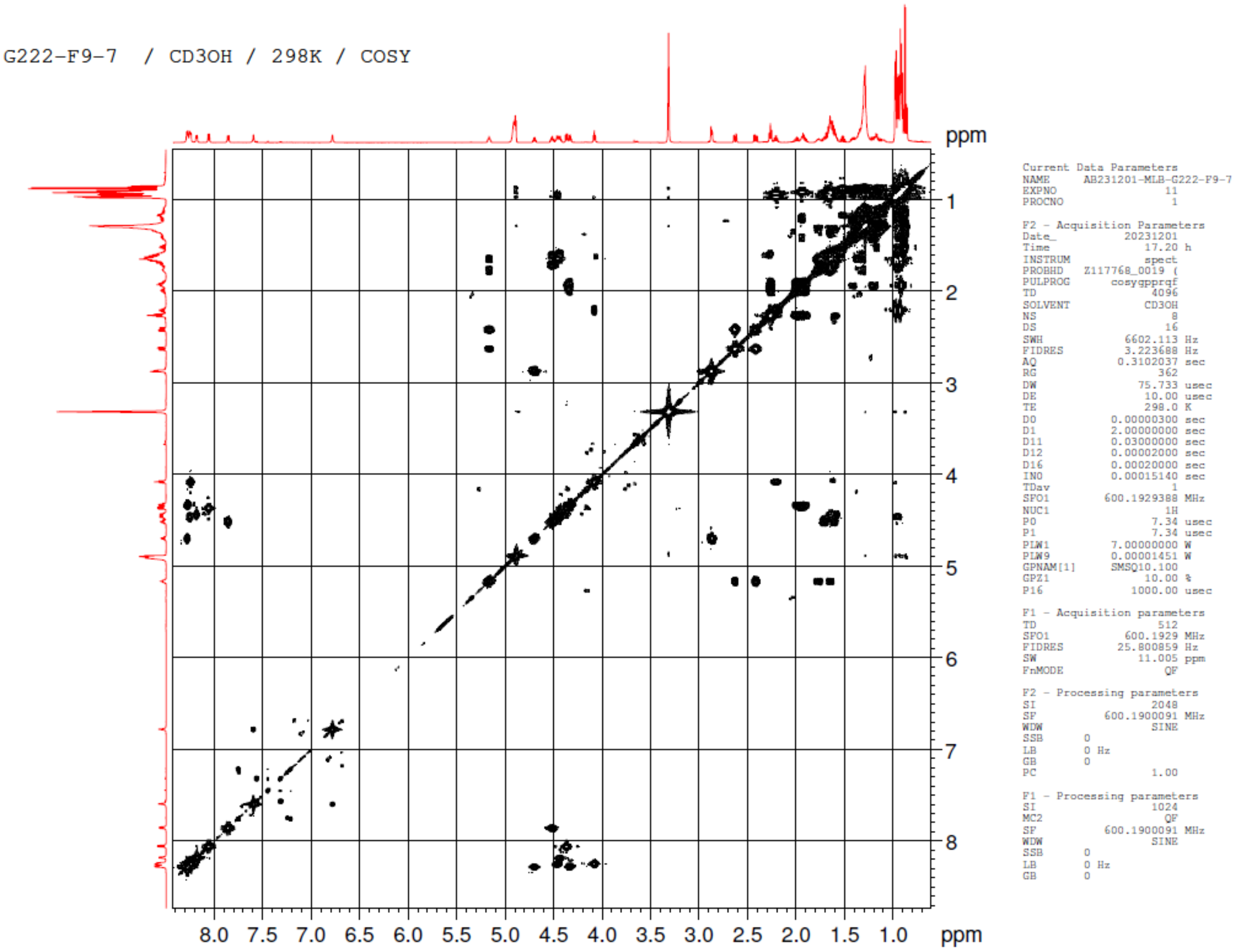

Figure S38: COSY-NMR spectrum of Lichenysin (**6-7**) (*m/z* 1035.7063 [M + H]$^+$) (600 MHz, CD_3OH).

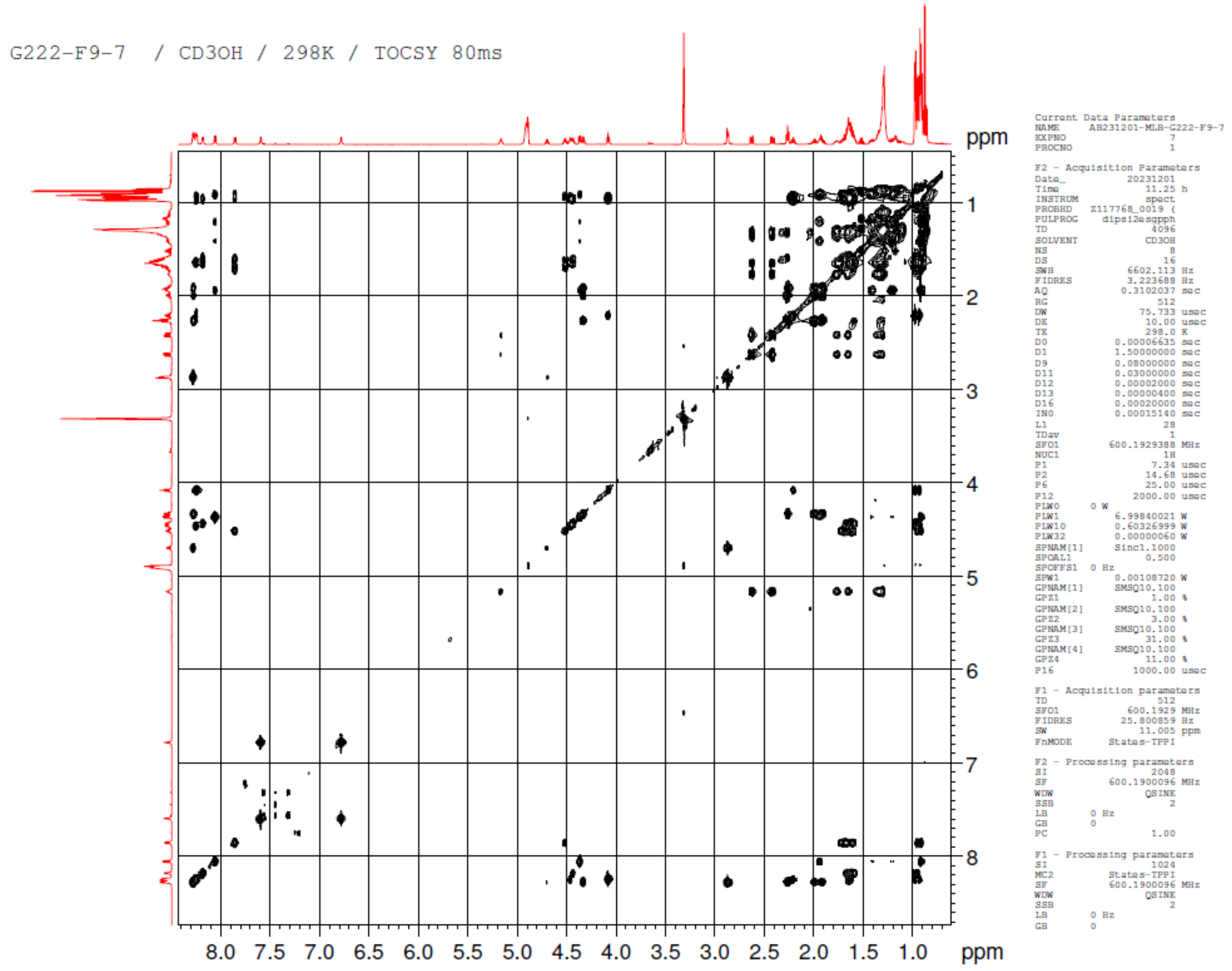

Figure S39: TOCSY-NMR spectrum of Lichenysin (**6-7**) (*m*/*z* 1035.7063 [M + H]+) (600 MHz, CD_3OH).

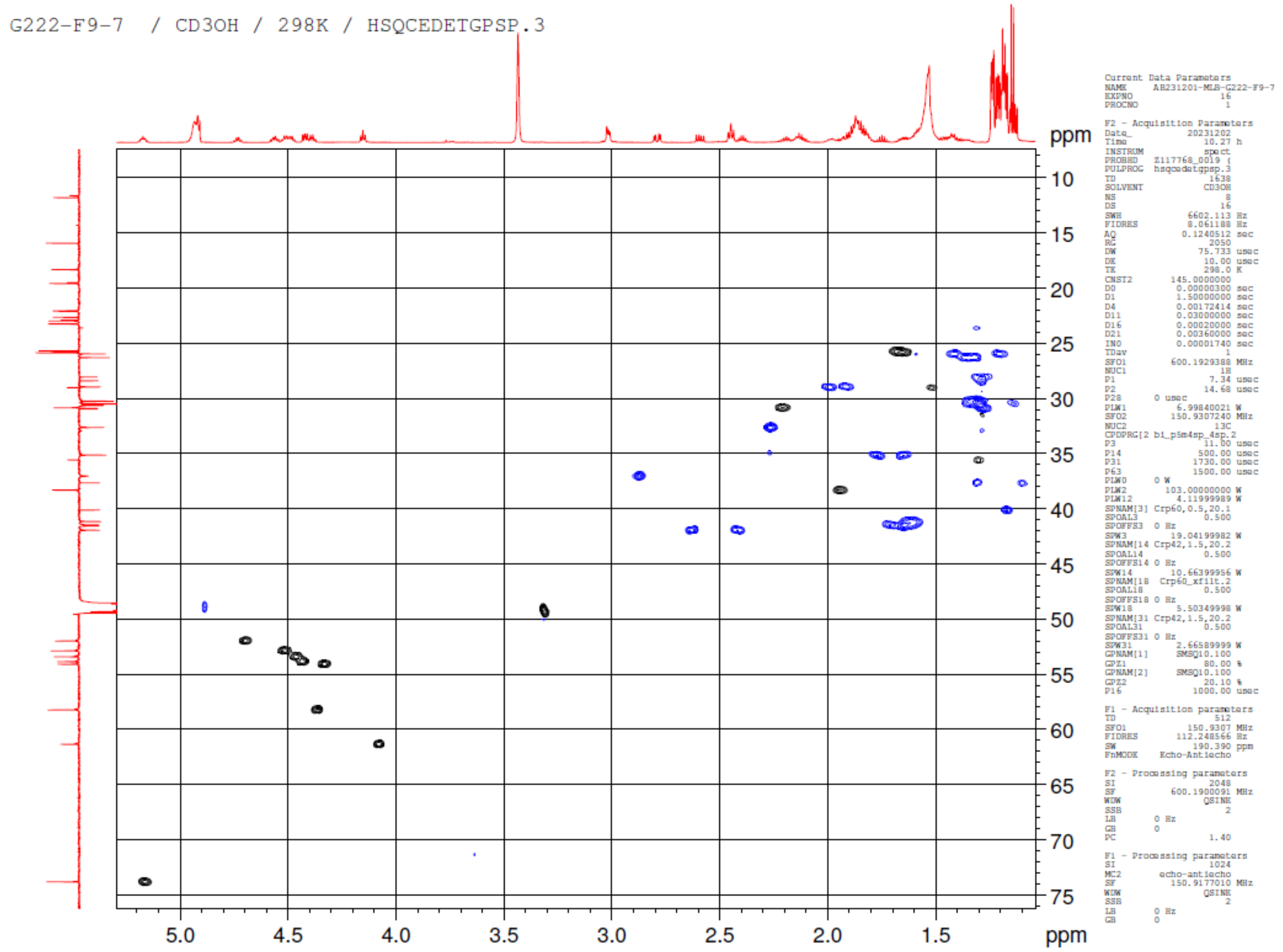

Figure S40: HSQC-NMR spectrum of Lichenysin (**6-7**) (*m*/*z* 1035.7063 [M + H]$^+$) (600 MHz, CD_3OH).

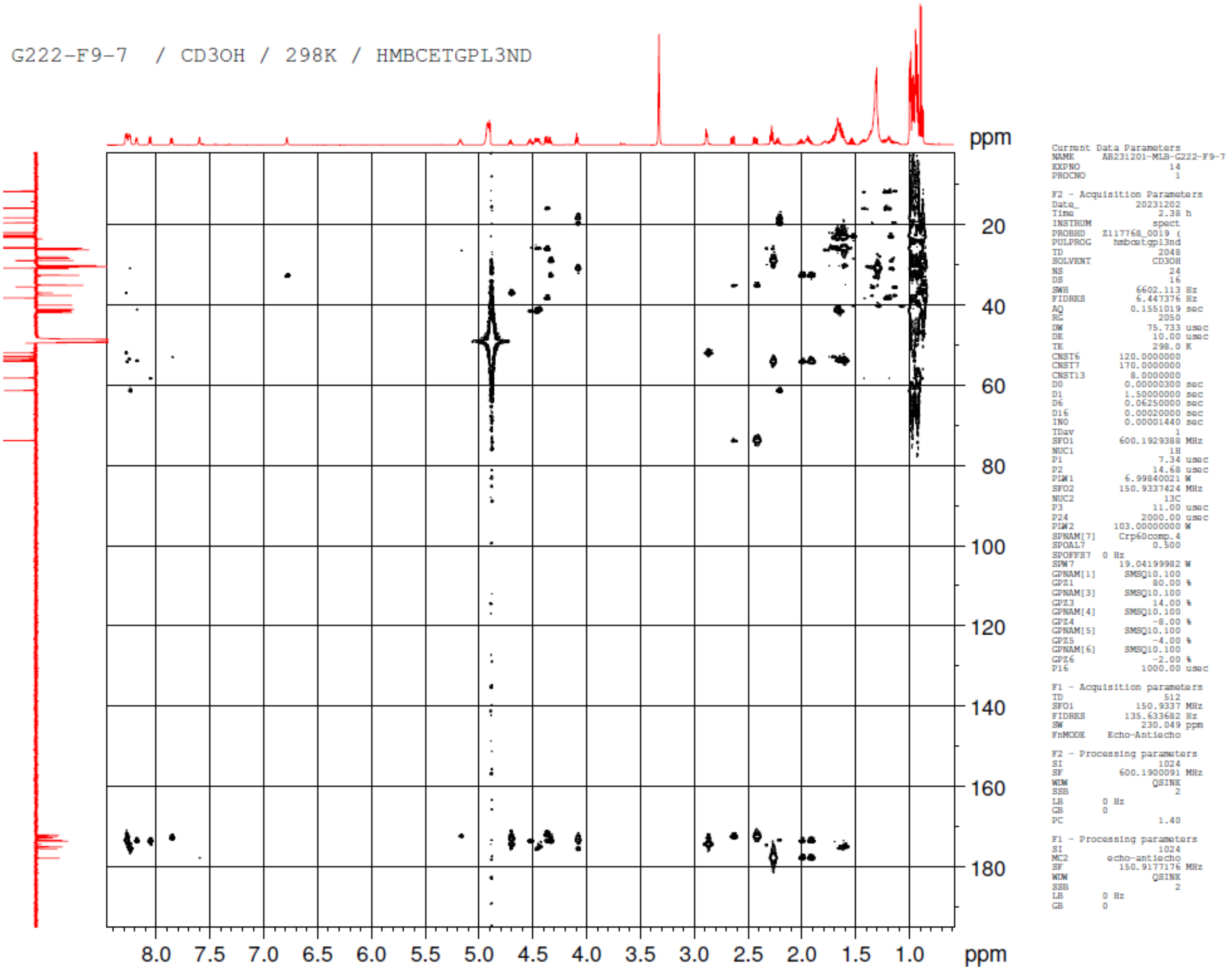

Figure S41: HMBC-NMR spectrum of Lichenysin (**6-7**) (*m*/*z* 1035.7063 [M + H]+) (600 MHz, CD_3OH).

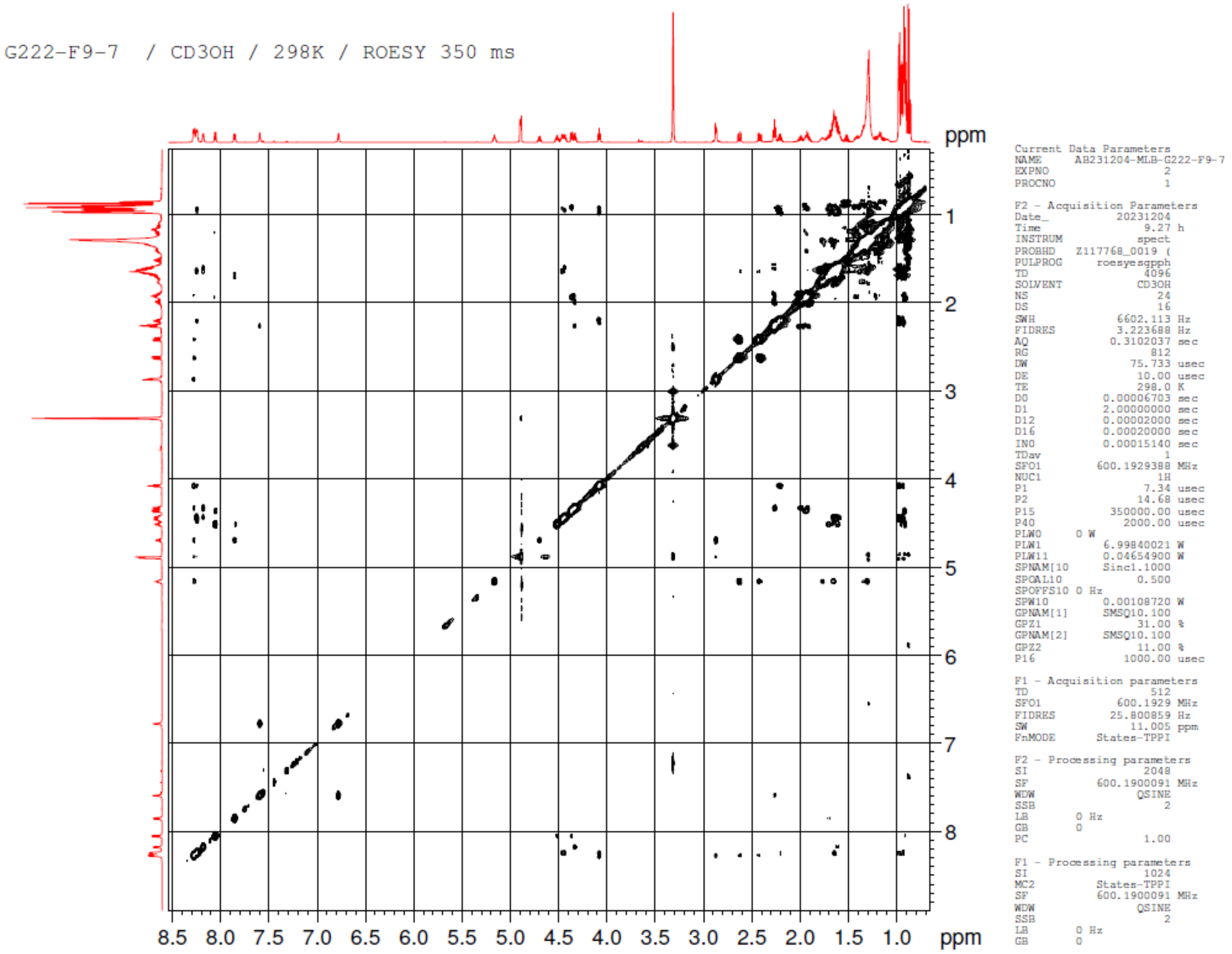

Figure S42: ROESY-NMR spectrum of Lichenysin (**6-7**) (*m*/*z* 1035.7063 [M + H]⁺) (600 MHz, CD_3OH).

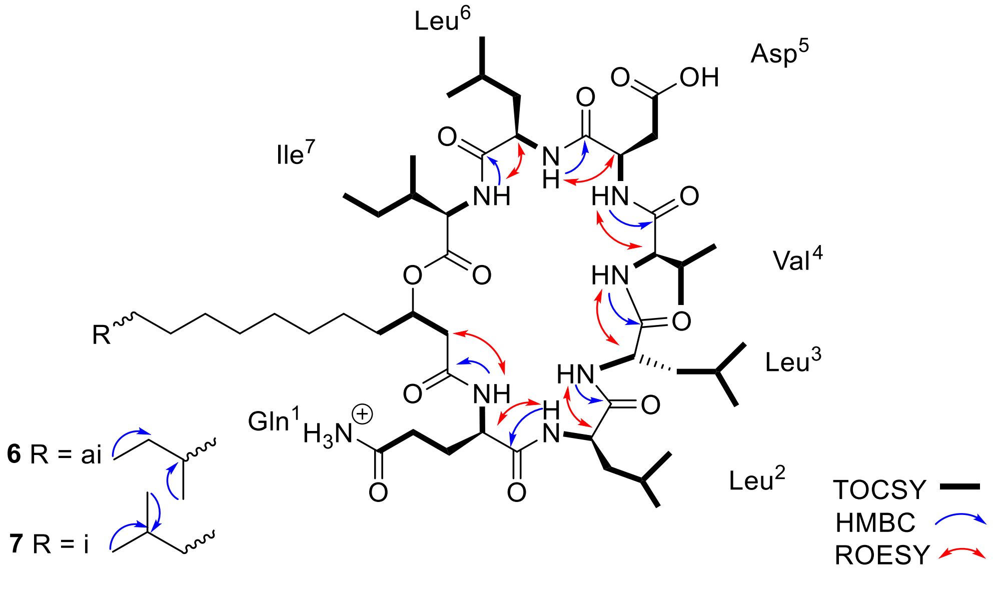

Figure S43: Structure of Lichenysins (**6-7**) (*m*/*z* 1035.7063 [M + H]⁺).

Table S18. ^{1}H, ^{13}C, HMBC, and ROESY NMR data of Lichenysin (**6**-**7**) (*m*/*z* 1035.7064 [M + H]$^{+}$) (600 MHz, CD_3OH) isoform: *ante-iso* and *iso* (0.2:0.8).

Unit	Pos.	δC, Mult.		δH (*J* in Hz)	HMBC	ROESY [a]
L-Gln¹	NH	-		8.26, d (7.56)	Gln¹-α, FA-CO,	FA-2-a, FA-2-b, FA-3
	C=O	173.62, C		-	-	-
	α-C	54.12, CH		4.33, ddd (7.56, 11.13, 13.23)	Gln¹-CO, Gln¹-β, Gln¹- γ	Leu²-NH
	β-C	28.96, CH_2	a.<br>b.	1.98, m<br>1.91, m	Gln¹-CO, Gln¹-α, Gln¹-γ, Gln¹-δ-C=O	Leu²-NH
	γ-C	32.63, CH_2		2.26, t (7.56)	Gln¹-α, Gln¹-β, Gln¹-δ-C=O	Leu²-NH
	δ-C=O	177.89, CO²		-	-	-
	ε-NH_2	-	a.<br>b.	7.59, brs<br>6.77 brs	Gln¹-δ-C=O, Gln¹- γ	-
L-Leu²	NH	-		8.17, d (6.35)	Gln¹-CO	Gln¹-α, Gln¹-β, Gln¹-γ
	C=O	175.09, C		-	-	-
	α-C	53.90, CH		4.43, ddd (5.09, 6.35, 14.25)	Leu²-CO, Leu²-β, Leu²-γ	Leu³-NH
	β-C	41.17, CH_2	a.<br>b	1.64, m<br>1.61, m	Leu²-CO, Leu²-α, Leu²-γ	Leu³-NH
	γ-C	25.87, CH		1.64, m	Leu²-α, Leu²-β, Leu²-δ, Leu²-δ′	Leu³-NH
	δ-C	22.98, CH_3		0.97, d (4.88)	Leu²-β, Leu²-γ, Leu²-δ′	-
	δ′-C	23.28, CH_3		0.95, d (4.88)	Leu²-β, Leu²-γ, Leu²-δ	-
D-Leu³	NH	-		8.25, d (6.63)	Leu²-CO, Leu³-α	Leu²-α, Leu²-β, Leu²-γ
	C=O	175.52, C		-	-	-
	α-C	53.44, CH		4.45, ddd (5.94, 6.63, 14.25)	Leu³-CO, Leu³-β, Leu³-γ	Val⁴-NH
	β-C	41.56, CH_2	a.<br>b	1.61, m<br>1.64, m	Leu³-CO, Leu³-α, Leu³-γ	Val⁴-NH
	γ-C	25.70, CH		1.64, m	Leu³-α, Leu³-β	Val⁴-NH
	δ-C	22.07, CH_3		0.91, d (3.95)	Leu³-β, Leu³-γ, Leu³-δ′	-
	δ′-C	23.26, CH_3		0.97, d (4.88)	Leu³-β, Leu³-γ, Leu³-δ	-
L-Val⁴	NH	-		8.24, d (6.70)	Val⁴-α, Val⁴-β, Leu³-CO	Leu³-α, Leu³-β, Leu³-γ
	C=O	173.37 C		-	-	-
	α-C	61.35, CH		4.09, dd (5.26, 6.70)	Val⁴-CO, Val⁴-β, Val⁴-γ, Val⁴-γ′	Asp⁵-NH
	β-C	30.93, CH		2.20, ddd (5.26, 6.70, 13.20)	Val⁴-α, Val⁴-γ, Val⁴-γ′	Asp⁵-NH
	γ-C	19.62, CH_3		0.97, d (4.88)	Val⁴-β, Val⁴-γ′	Asp⁵-NH
	γ′-C	18.31, CH_3		0.92, d (3.95)	Val⁴-β, Val⁴-γ	Asp⁵-NH
L-Asp⁵	NH	-		8.28, d (7.43)	Val⁴-CO, Asp⁵-α	Val⁴-α, Val⁴-β, Val⁴-γ, Val⁴-γ′, Leu⁶-NH
	C=O	172.99, C		-	-	-
	α-C	52.01, CH		4.70, dd (7.43, 12.55)	Asp⁵-CO, Asp⁵-β, Asp⁵-γ-C=O	Leu⁶-NH
	β-C	37.10, CH_2		2.84, dd (3.19, 7.43)	Asp⁵-CO, Asp⁵-α, Asp⁵-γ-C=O	Leu⁶-NH
	γ-C=O	174.49, CO²		-	-	-
D-Leu⁶	NH	-		7.85, d (8.63)	Asp⁵-CO	Asp⁵-α, Asp⁵-NH, Ile⁶-NH
	C=O	173.60, C		-	-	-
	α-C	52.91, CH		4.51, ddd (5.77, 8.63, 14.59)	Leu⁶-CO, Leu⁶-β,	Ile⁷-NH
	β-C	41.45, CH_2	a.<br>b	1.70, m<br>1.60, m	Leu⁶-CO, Leu⁶-α, Leu⁶-γ	Ile⁷-NH
	γ-C	25.86, CH		1.64, m	Leu⁶-α, Leu⁶-β	Ile⁷-NH
	δ-C	22.07, CH_3		0.91, d (3.95)	Leu⁶-β, Leu³-δ	-
	δ′-C	22.65, CH_3		0.94, d (3.58)	Leu⁶-β, Leu⁶-δ′	-

Unit	Pos.	δ_C, Mult.		δ_H (*J* in Hz)	HMBC	ROESY [a]
L-Ile^{7}	NH	-		8.05, d (8.27)	Leu^{6}-CO	Leu^{6}-NH, Leu^{6}-α, Leu^{6}-β, Leu^{6}-γ
	C=O	172.12, C		-	-	-
	α-C	58.21, CH		4.36, dd (5.60, 8.27)	Ile^{7}-CO, Ile^{7}-β, Ile^{7}-γ, Ile^{7}-δ	-
	β-C	38.37, CH		1.93, q (8.27)	Ile^{7}-β, Ile^{7}-γ, Ile^{7}-δ, Ile^{7}-δ′	-
	γ-C	25.99, CH$_2$	a.<br>b	1.41, m<br>1.20, m	Ile^{7}-β, Ile^{7}-δ, Ile^{7}-δ′	-
	δ-C	15.96, CH$_3$		0.92, d (4.31)	Ile^{7}-α, Ile^{7}-β, Ile^{7}-γ	-
	δ′-C	11.79, CH$_3$		0.89, t (2.33)	Ile^{7}-β, Ile^{7}-γ	-
Fatty acid	C=O	172.55, C		-	-	-
	2	41.96, CH$_2$	a.<br>b	2.62, dd (4,58, 12.21)<br>2.43, dd (7,46, 14.31)	FA-3, FA-4, FA-CO	Gln^{1}-NH
	3	73.83, CH		5.15 ddd (4.58, 7.46, 12.21)	FA-4, FA-CO	Gln^{1}-NH
	4	35.14, CH$_2$	a.<br>b	1.77, m<br>1.65, m	FA-2-a, FA-2-b, FA-3<br>FA-3, FA-5-11	-<br>-
ai	5	26.32, CH$_2$*		1.35^{b}, m	FA-5-11, FA-12, FA-13	-
ai	6	28.07, CH$_2$*		1.29^{b}, m	FA-5-11, FA-12, FA-13	-
ai	7	30.24, CH$_2$*		1.29^{b}, m	FA-5-11, FA-12, FA-13	-
ai	8	30.51, CH$_2$*		1.29^{b}, m	FA-5-11, FA-12, FA-13	-
ai	9	30.52, CH$_2$*		1.29^{b}, m	FA-5-11, FA-12, FA-13	-
ai	10	30.65, CH$_2$*		1.29^{b}, m	FA-5-11, FA-12, FA-13	-
ai	11	37.67, CH$_2$	a.<br>b	1.09, m<br>1.29, m	FA-5-11, FA-12, FA-13	-
ai	12	35.54, CH$_2$		1.29^{b}, m	FA-13, FA-5-11	-
ai	13	30.53, CH	a.<br>b	1.29^{b}, m<br>1.13, m	FA-12, FA-14, FA-15	-
ai	14	19.50, CH$_3$		0.85, d (4.84)	FA-12	-
ai	15	11.63, CH$_3$		0.86, t (6.15)	FA-11, FA-12, FA-13	-
i	5	26.33, CH$_2$*		1.35^{b}, m	FA-5-11, FA-12, FA-13	-
i	6	28.42, CH$_2$*		1.29^{b}, m	FA-5-11, FA-12, FA-13	-
i	7	30.40, CH$_2$*		1.29^{b}, m	FA-5-11, FA-12, FA-13	-
i	8	30.51, CH$_2$*		1.29^{b}, m	FA-5-11, FA-12, FA-13	-
i	9	30.53, CH$_2$		1.29^{b}, m	FA-5-11, FA-12, FA-13	-
i	10	30.65, CH$_2$*		1.29^{b}, m	FA-5-11, FA-12, FA-13	-
i	11	26.32, CH$_2$		1.29^{b}, m	FA-12, FA-14, FA-15	-
i	12	40.13, CH$_2$		1.17, m	FA-11, FA-14, FA-15	-
i	13	29.02, CH$_2$		1.51, m	FA-12, FA-14, FA-15	-
i	14	22.91, CH$_3$		0.87, d (6.55)	FA-11, FA-13, FA-15	-
i	15	22.91, CH$_3$		0.87, d (6.55)	FA-11, FA-13, FA-14	-

[a] Sequential ROEs [b] Overlapped signals prevent determination of constant couplings * May be interchanged.

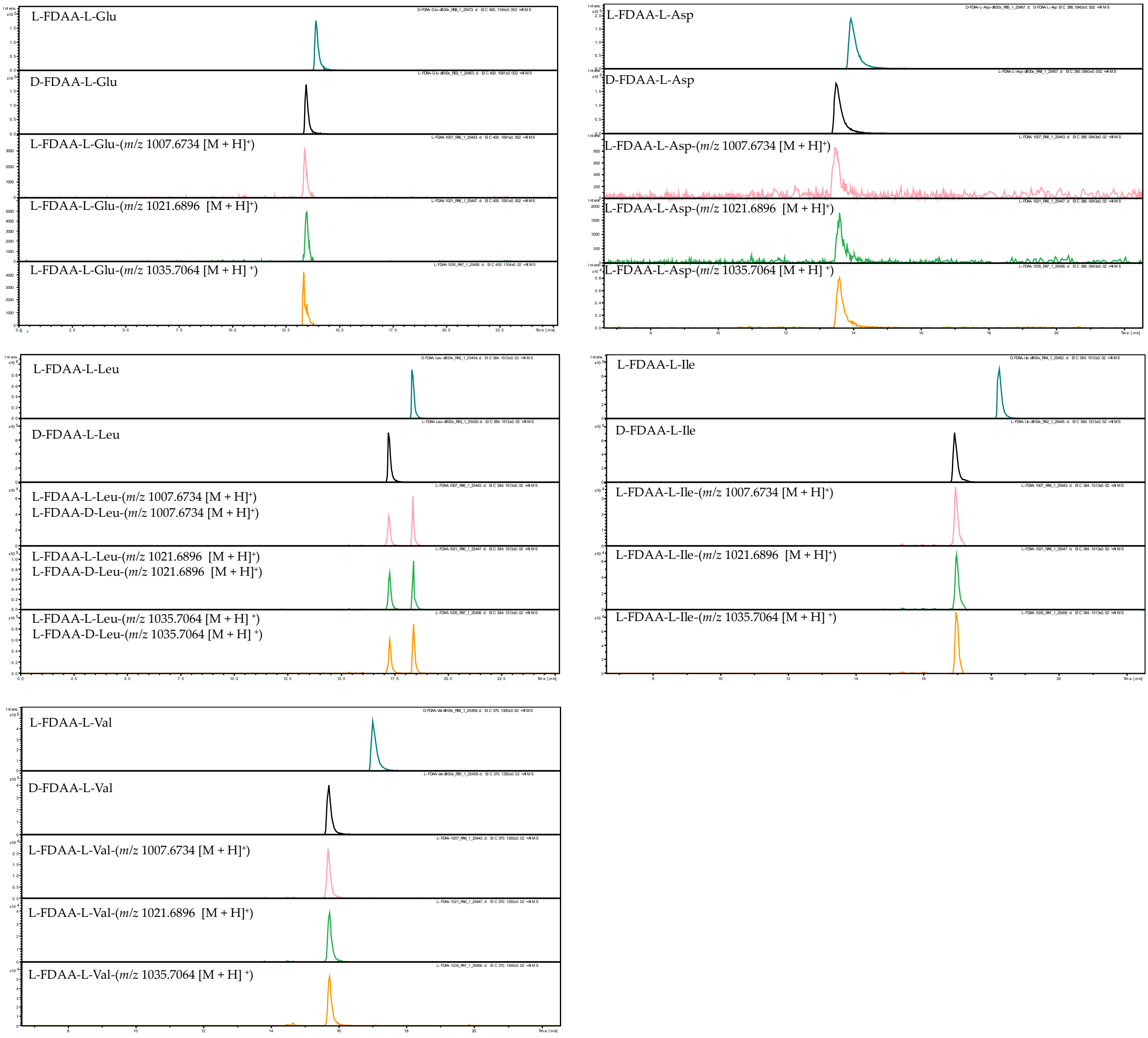

Figure S44: Marfey's analysis in positive ion mode high-resolution ESI mass spectrum

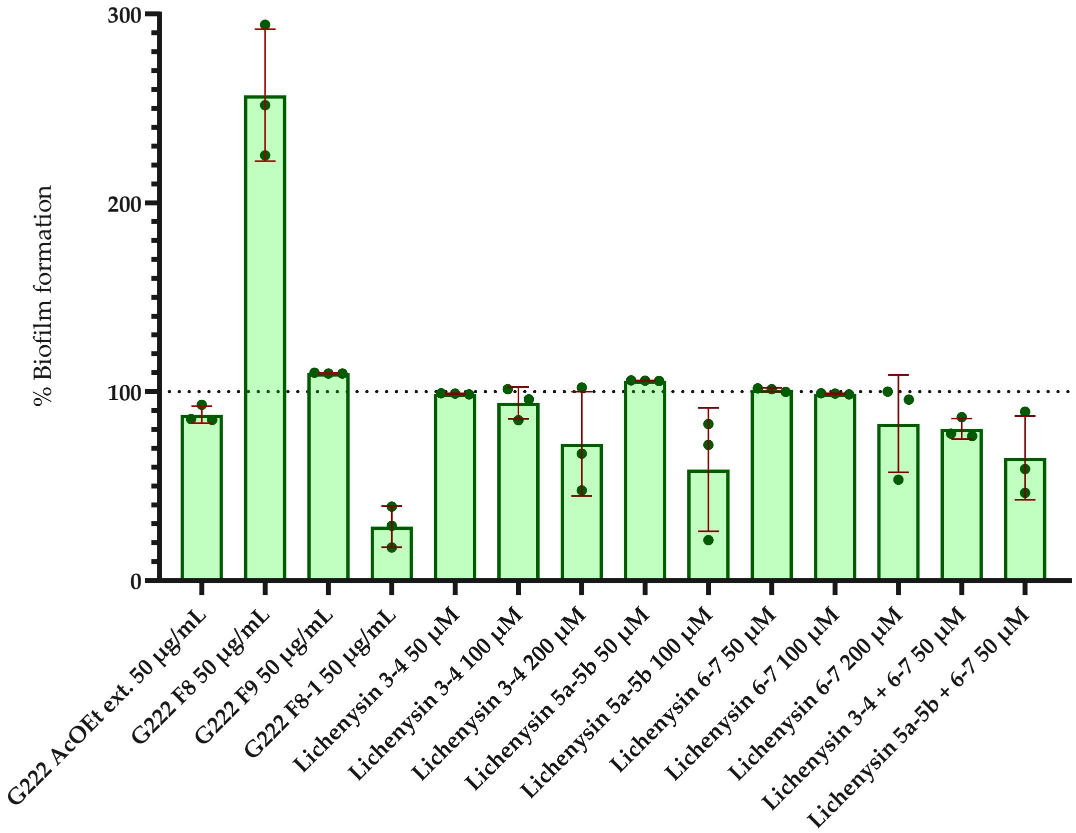

Figure S45: Biofilm-forming activity against *Pseudomonas aeruginosa* MUC-N1 (n = 3).

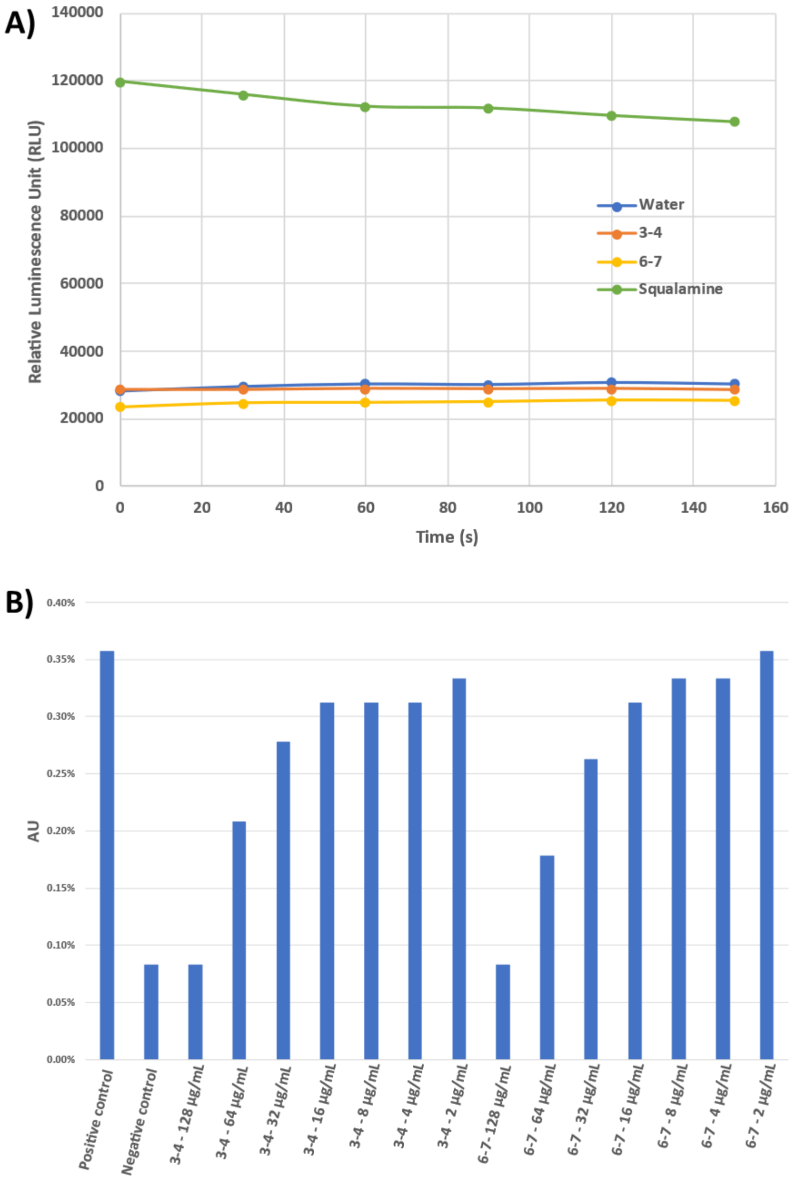

Figure S46: A) ATP release in *S. aureus* MRSA exhibited by compounds (**3**-**4**) and (**6**-**7**) as determined using ATP efflux assay. Squalamine (100 µg.mL^{-1}) was the positive control and water was the negative control. Compounds were tested at a final concentration of 100 µg.mL^{-1}, and results are reported as relative luminescence unit. B) Bacterial growth inhibition exhibited by (**3**-**4**) and (**6**-**7**) against *S. aureus* MRSA (CF-Marseille) with different concentrations. Positive control was bacteria only and negative control was media only.
